# Glucose controls lipolysis through Golgi PtdIns4P-mediated regulation of ATGL

**DOI:** 10.1038/s41556-024-01386-y

**Published:** 2024-04-01

**Authors:** Lianggong Ding, Florian Huwyler, Fen Long, Wu Yang, Jonas Binz, Kendra Wernlé, Matthias Pfister, Manuel Klug, Miroslav Balaz, Barbara Ukropcova, Jozef Ukropec, Chunyan Wu, Tongtong Wang, Min Gao, Pierre-Alain Clavien, Philipp Dutkowski, Mark W. Tibbitt, Christian Wolfrum

**Affiliations:** 1 Institute of Food, Nutrition and Health, ETH Zürich, Schwerzenbach, Switzerland; 2 Macromolecular Engineering Laboratory, Institute of Energy and Process Engineering, ETH Zürich, Zurich, Switzerland; 3 Interdisciplinary Research Center on Biology and Chemistry, Shanghai Institute of Organic Chemistry, Chinese Academy of Sciences, Shanghai, China; 4 Department of Surgery and Transplantation, University of Zurich, Zurich, Switzerland; 5 Biomedical Research Center, Slovak Academy of Sciences, Bratislava, Slovakia; 6 Department of Pharmacy, the Sixth Affiliated Hospital, Sun Yat-sen University, Guangzhou, China; 7 Wyss Zurich Translational Center, ETH Zurich and University of Zurich, Zurich, Switzerland

**Keywords:** Golgi, Metabolic syndrome

## Abstract

Metabolic crosstalk of the major nutrients glucose, amino acids and fatty acids (FAs) ensures systemic metabolic homeostasis. The coordination between the supply of glucose and FAs to meet various physiological demands is especially important as improper nutrient levels lead to metabolic disorders, such as diabetes and metabolic dysfunction-associated steatohepatitis (MASH). In response to the oscillations in blood glucose levels, lipolysis is thought to be mainly regulated hormonally to control FA liberation from lipid droplets by insulin, catecholamine and glucagon. However, whether general cell-intrinsic mechanisms exist to directly modulate lipolysis via glucose sensing remains largely unknown. Here we report the identification of such an intrinsic mechanism, which involves Golgi PtdIns4P-mediated regulation of adipose triglyceride lipase (ATGL)-driven lipolysis via intracellular glucose sensing. Mechanistically, depletion of intracellular glucose results in lower Golgi PtdIns4P levels, and thus reduced assembly of the E3 ligase complex CUL7^FBXW8^ in the Golgi apparatus. Decreased levels of the E3 ligase complex lead to reduced polyubiquitylation of ATGL in the Golgi and enhancement of ATGL-driven lipolysis. This cell-intrinsic mechanism regulates both the pool of intracellular FAs and their extracellular release to meet physiological demands during fasting and glucose deprivation. Moreover, genetic and pharmacological manipulation of the Golgi PtdIns4P–CUL7^FBXW8^–ATGL axis in mouse models of simple hepatic steatosis and MASH, as well as during ex vivo perfusion of a human steatotic liver graft leads to the amelioration of steatosis, suggesting that this pathway might be a promising target for metabolic dysfunction-associated steatotic liver disease and possibly MASH.

## Abstract (web summary)

Ding et al. find a mechanism coordinating fatty acid and glucose supply. Glucose-driven Golgi phosphatidylinositol 4-phosphate levels impact the assembly of E3 ligase complex CUL7–FBXW8, controlling adipose triglyceride lipase levels and lipolysis.

## Main

The catabolism of glucose, amino acids and fatty acids (FAs) is a central function of almost all cell types to maintain energy requirements for their various cellular activities. FAs have the highest energy density and most are esterified into triglycerides (TGs) and stored in lipid droplets (LDs) to avoid toxicity^1^. During starvation, LDs are mobilized to liberate FAs for satisfying cellular energy demands via lipolysis or autophagic degradation of LDs (that is, lipophagy)^1–3^. Lipolysis is executed sequentially by adipose triglyceride lipase (ATGL), hormone-sensitive lipase (HSL) and monoacylglycerol lipase (MGL), while lipophagy is dependent on lysosomal acid lipase^1,4,5^. Although LDs can form in almost any cell types, adipose tissue has the highest LD abundance. In the presence of an adequate glucose supply, insulin signalling increases lipogenesis while repressing lipolysis to promote lipid storage^1,6,7^. Conversely, hormonal or sympathetic-neuronal stimulation activates lipolysis to supply FAs^1,8^.

HSL is activated by phosphorylation through catecholamine signalling activated protein kinase A (PKA)^9^. Insulin suppresses this process via phosphodiesterase 3B (PDE3B)-mediated cAMP degradation^10,11^. Nevertheless, loss of HSL leads to mild phenotypes in both humans and mice^1,5,12^. For example, liver-specific depletion of HSL does not impact hepatic TG levels and loss of HSL in adipose tissue leads to an age-dependent hepatic steatosis^13^. In contrast, *Atgl* loss of function in mice or humans leads to substantial LD accumulation^14,15^. Liver-specific *Atgl* knockout mice exhibit hepatic LD accumulation, whereas adipose tissue-specific *Atgl* knockout mice display substantially reduced lipid levels in the liver^16,17^. ATGL function is modulated at multiple levels to meet complex physiological demands. Transcriptionally, insulin signalling inhibits *ATGL* messenger RNA synthesis^6^. At the post-translational level, catecholamine and glucagon signalling promote ATGL enzymatic activity through PKA- and CAMKII-mediated phosphorylation of ATGL at Ser406 in adipocytes and hepatocytes^18,19^. In addition, ATGL abundance can be regulated by proteasome-mediated degradation after polyubiquitination via different E3 ligases, such as COP1 and PEX2 (refs. ^20,21^).

The Golgi apparatus incorporates integral proteins spanning the membrane or recruits cytosolic proteins via interaction with Golgi-enriched lipids, such as PtdIns4P^22^. Owing to its negatively charged property, PtdIns4P can interact with proteins containing positively charged regions, such as NLRP3 and STUB1, thereby enabling Golgi PtdIns4P to regulate inflammation and protein degradation, respectively^23,24^. The Golgi PtdIns4P pool is mainly generated by the lipid kinases PI4KB^22^. Conversely, the lipid phosphatase SACM1L dephosphorylates PtdIns4P^22^. SACM1L activity is modulated via altered distribution in the ER and Golgi, and enrichment of SACM1L in the latter compartment is promoted by glucose or serum deprivation, leading to a reduction of PtdIns4P levels in the Golgi^25,26^.

So far, hormone signalling is still the best-documented mechanism to coordinate glucose availability and systemic lipolysis. In this Article, we aimed to investigate cell-intrinsic mechanisms that balance the supply of glucose and FAs by regulation of lipolysis via intracellular glucose sensing. We identified a central role for PtdIns4P in the assembly of the E3 ligase complex CUL7^FBXW8^ in the Golgi, which in turn polyubiquitylates ATGL for proteasome-targeted degradation. Depletion of intracellular glucose is sensed by this pathway, leading to reduced assembly of the E3 ligase complex and increased ATGL-driven lipolysis.

## Results

### Glucose deprivation elevates ATGL-driven lipolysis

**Fig. 1:**
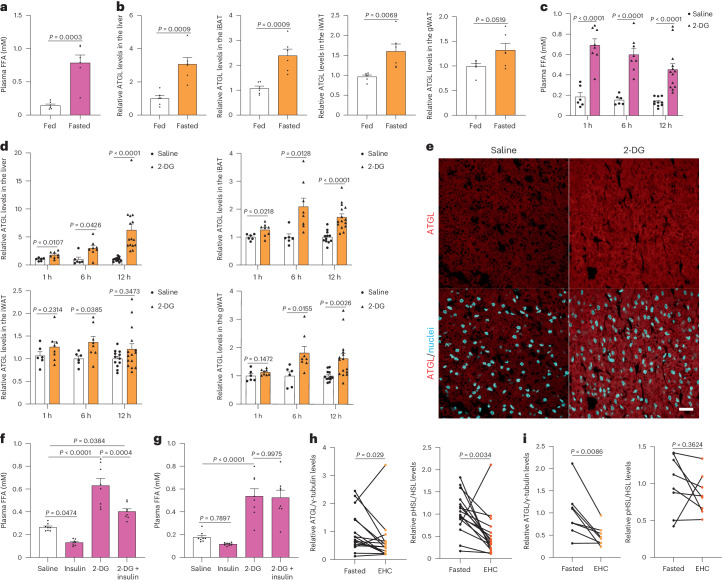
Glucose deprivation increases lipolysis via ATGL stabilization in the adipose depots and liver. **a**,**b**, Quantification of plasma FFA levels (**a**) and ATGL levels (**b**) in the liver and adipose depots after 24 h fasting (*n* = 6 mice per group). **c**, Plasma FFA levels in the mice 1 h, 6 h and 12 h after 2-DG (2 g kg^−1^ BW) administration (1 h and 6 h, *n* = 6 mice for saline and 8 mice for 2-DG group; 12 h, *n* = 10 mice for saline and 13 mice for 2-DG group). **d**, Quantification of ATGL levels in the liver and adipose depots collected 1 h, 6 h and 12 h after 2-DG administration (1 h and 6 h, *n* = 6 mice for saline and 8 mice for 2-DG group; 12 h, *n* = 12 mice for saline and 15 mice for 2-DG group). **e**, Representative immunofluorescence of ATGL in the iBAT collected 6 h after 2-DG administration. Scale bar, 20 μm. Representative images from three mice per group with similar results. **f**, Plasma FFA levels in the wild-type mice after 2-DG treatment for 5.5 h, followed by insulin (0.75 U kg^−1^ BW) administration for 30 min (*n* = 8 mice per group). **g**, Plasma FFA levels in the wild-type mice 6 h after co-administration of 2-DG and insulin (*n* = 8 mice per group). **h**,**i**, Quantification of ATGL and HSL^Ser660ph^ levels in the subcutaneous adipose tissue taken before and after EHC from patients with insulin sensitivity or resistance (*n* = 16 patients with insulin sensitivity in **h**; *n* = 8 patients with insulin resistance in **i**). Data are presented as mean ± s.e.m. and analysed using two-tailed unpaired *t*-test (**a**–**d** (1 h in liver, iBAT, 1 h and 6 h in iWAT, and 1 h and 6 h in gWAT)), two-tailed paired *t*-test (**i**), two-tailed Mann–Whitney test (**d** (6 h and 12 h in liver, 12 h in iWAT and 12 h in gWAT)), two-tailed Wilcoxon test (**h**) and one-way ANOVA method with Tukey correction (**f** and **g**). Source numerical data are available in . Source data

**Extended Data Fig. 1:**
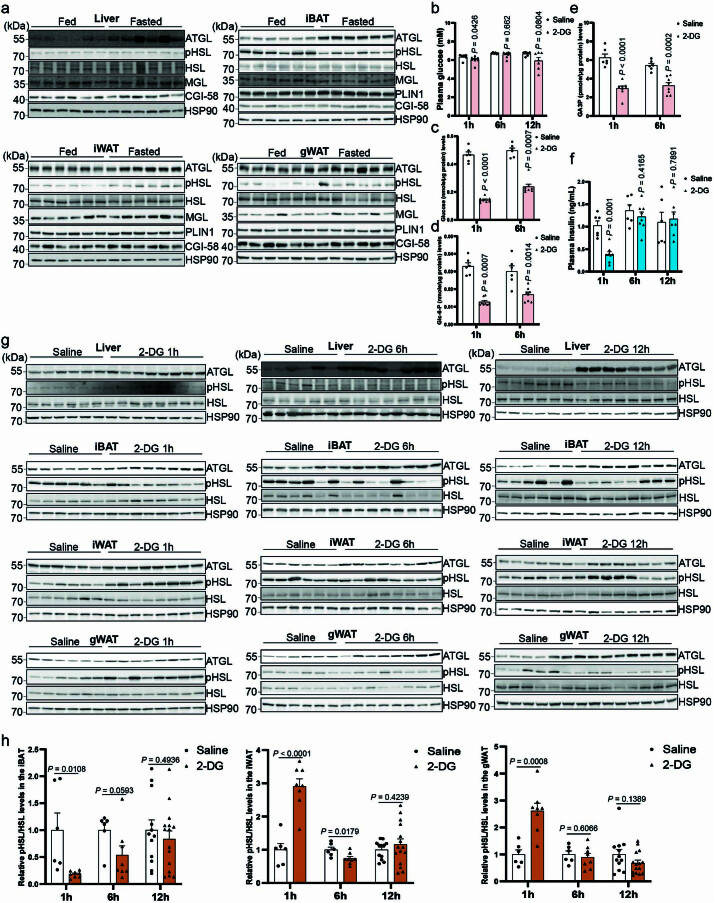
ATGL-driven lipolysis is increased in the liver and adipose depots from fasted and glucose-deprived mice. (**a**) Immunoblots of ATGL, HSL^Ser660ph^, HSL, MGL, PLIN1, CGI-58 and HSP90 in the liver and adipose depots from fed and fasted mice. (**b**) Plasma glucose levels in the wild-type mice 1 h, 6 h and 12 h after 2-DG (2 g/kg BW) administration (1 h and 6 h, n = 6 mice for saline and 8 mice for 2-DG group; 12 h, n = 6 mice for saline and 7 mice for 2-DG group). (**c–e**) Levels of glucose and glucose derivatives (glucose-6-p and GA3P) in the livers of wild-type mice collected 1 h and 6 h after 2-DG (2 g/kg BW) administration (n = 6 mice for saline and 8 mice for 2-DG group). (**f**) Plasma insulin levels in the wild-type mice 1 h, 6 h and 12 h after 2-DG (2 g/kg BW) administration (1 h and 6 h, n = 6 mice for saline and 8 mice for 2-DG group; 12 h, n = 6 mice for saline and 7 mice for 2-DG group). (**g**) Immunoblots of ATGL, HSL^Ser660ph^, HSL and HSP90 in the liver and adipose depots collected 1 h, 6 h and 12 h after 2-DG (2 g/kg BW) administration. (**h**) Quantification of HSL^Ser660ph^ levels in the adipose depots collected 1h, 6 h and 12 h after 2-DG (2 g/kg BW) administration (1 h and 6 h, n = 6 mice for saline and 8 mice for 2-DG group; 12 h, n = 12 mice for saline and 15 mice for 2-DG group). Results are shown as mean ± s.e.m. and analyzed using two-tailed unpaired *t*-test (b (12 h), c (1 h), d (6 h), e, f and h (1 h and 12 h in iBAT, iWAT and gWAT)) and two-tailed Mann-Whitney test (b (1 h and 6 h), c (6 h), d (1 h) and h (6 h in iBAT)). Source numerical data and unprocessed blots are available in source data. Source data

**Extended Data Fig. 2:**
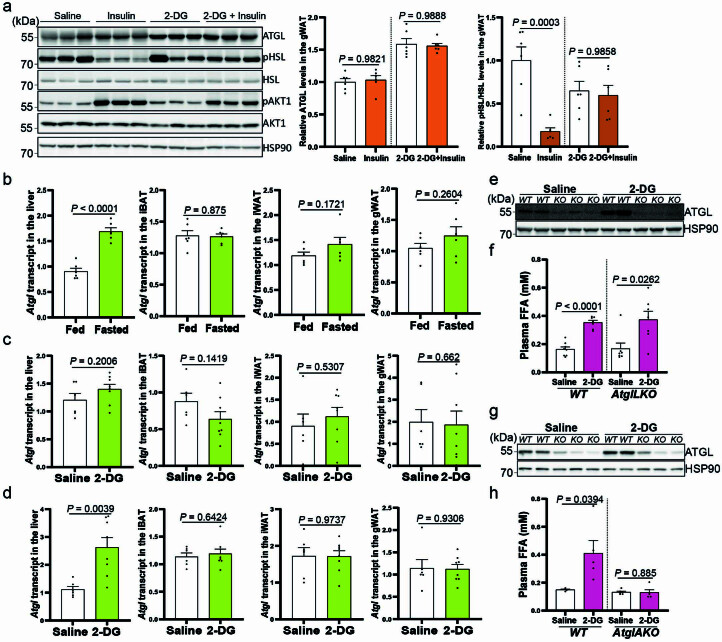
Glucose deprivation elevates systemic lipolysis via upregulating ATGL in the adipose depots independently of transcriptional changes. (**a**) Immunoblots of ATGL, HSL^Ser660ph^, HSL, AKT1, AKT1^Ser473ph^ and HSP90 in the gWAT from wild-type mice after 2-DG (2 g/kg BW) treatment for 5.5 h, followed by insulin (0.75 U/kg BW) administration for 30 minutes (n = 6 mice per group). (**b**) Quantification of *Atgl* transcript levels in the liver and adipose depots from fasted mice via qPCR (n = 6 mice per group). (**c**) Quantification of *Atgl* transcript levels in the liver and adipose depots collected 1 h after 2-DG (2 g/kg BW) administration (n = 6 mice for saline and 8 mice for 2-DG group). (**d**) Quantification of *Atgl* transcript levels in the liver and adipose depots collected 6 h after 2-DG (2 g/kg BW) administration (n = 6 mice for saline and 8 mice for 2-DG group). (**e–f**) Immunoblot of ATGL in the liver and determination of plasma FFA levels in liver-specific *Atgl* knockout mice (*AtglLKO*) 6 h after 2-DG (2 g/kg BW) administration (*WT*: n = 8 mice for saline and 7 mice for 2-DG group; *AtglLKO* mice: n = 7 mice per group). (**g–h**) Immunoblot of ATGL in the iBAT and determination of plasma FFA levels in adipose tissue-specific *Atgl* knockout mice (*AtglAKO*) 6 h after 2-DG (2 g/kg BW) administration (*WT*: n = 4 mice for saline and 5 mice for 2-DG group; *AtglAKO*: n = 6 mice for saline and 5 mice for 2-DG group). Results are shown as mean ± s.e.m. and analyzed using two-tailed unpaired *t*-test (b, c (liver, iBAT and iWAT), d, f (*WT*) and h), two-tailed Mann-Whitney test (c (gWAT) and f (*AtglLKO*)) and one-way ANOVA method with Tukey correction (a). Source numerical data and unprocessed blots are available in source data. Source data

Previous work demonstrated that intracellular FAs modulate ATGL-driven lipolysis^20^. To elucidate whether other cell-autonomous mechanisms modulate lipolysis in response to nutrient availability, we fasted mice for 24 h, which led to an increase in plasma free fatty acids (FFAs) (Fig. 1a). Next, we analysed the levels of lipolytic proteins, including ATGL, phosphorylated HSL, HSL, MGL, perilipin 1 (PLIN1) and comparative gene identification-58 (CGI-58), in the liver and adipose depots from fasted mice. Interestingly, only ATGL levels were significantly increased in the interscapular brown adipose tissue (iBAT), inguinal white adipose tissue (iWAT) and liver (Fig. 1b and Extended Data Fig. 1a), while both iWAT and gonadal white adipose tissue (gWAT) displayed upregulated pHSL levels (Extended Data Fig. 1a). To understand the time course of HSL phosphorylation and ATGL upregulation in response to long-term glucose deprivation, mice were treated with 2-deoxy-d-glucose (2-DG) to mimic glucose deprivation, in vivo (Extended Data Fig. 1b–e). Like fasting, glucose deprivation significantly induced lipolysis rapidly after 2-DG treatment (Fig. 1c), suggesting the molecular pathways involved may not rely on transcriptional regulation. To analyse the hormonal axis, we first determined levels of plasma insulin and observed reduction of insulin levels only 1 h after 2-DG administration (Extended Data Fig. 1f). Thus, we next quantified the main players involved in lipolysis in both liver and adipose depots at different timepoints. Intriguingly, upregulation of HSL phosphorylation was observed in WAT 1 h after 2-DG administration, while ATGL protein levels were increased in the liver and iBAT after 1 h and showed further accumulation in the liver, iBAT, iWAT and gWAT in response to longer glucose deprivation (Fig. 1d,e and Extended Data Fig. 1g,h), suggesting that ATGL regulation maintains higher lipolysis levels throughout glucose deprivation via a yet unknown mechanism. Hence, we tested the immediate effect of insulin on long-term glucose-deprived mice 6 h after 2-DG treatment. Unexpectedly, lipolysis levels were only partially suppressed and levels of pHSL and ATGL remained unchanged in the background of glucose deprivation 30 min after insulin treatment (Fig. 1f and Extended Data Fig. 2a). Moreover, the anti-lipolytic effect of insulin was totally absent during long-term glucose deprivation (Fig. 1g). These observations led us to postulate the existence of cell intrinsic mechanisms of ATGL-driven lipolysis regulation via sensing intracellular glucose availability, independently of hormone signalling. To assess human relevance, we obtained subcutaneous fat from patients collected before and after euglycaemic hyperinsulinaemic clamp (EHC), corresponding to the low and high circulated glucose states, respectively. Higher ATGL levels were observed in subcutaneous adipose tissues before EHC in patients with either insulin sensitivity or insulin resistance (Fig. 1h,i), suggesting that a similar ATGL regulation occurs in humans.

We examined *Atgl* transcript levels in liver and adipose depots from fasted or glucose-deprived mice and found that *Atgl* transcript levels were unchanged, further indicating that the regulation is not dependent on transcriptional events (Extended Data Fig. 2b–d). In contrast, long-term fasting and glucose deprivation promoted *Atgl* transcript levels in the liver (Extended Data Fig. 2b–d), possibly due to hepatic PPARα activation^8^. As adipose tissue is known to contribute to FA release^8^, we examined whether ATGL upregulation in the adipose tissue caused elevated plasma FFA levels during glucose deprivation. Therefore, we took advantage of liver- and adipose tissue-specific *Atgl* knockout mice (*AtglLKO* and *AtglAKO* mice) and found that only adipocytic ATGL was essential for increased plasma FFA levels in response to glucose deprivation (Extended Data Fig. 2e–h). Taken together, we demonstrate the existence of a cell-intrinsic mechanism of ATGL-driven lipolysis via sensing glucose availability, independently of insulin signalling.

### Glucose deprivation stabilizes ATGL in the Golgi apparatus

**Fig. 2:**
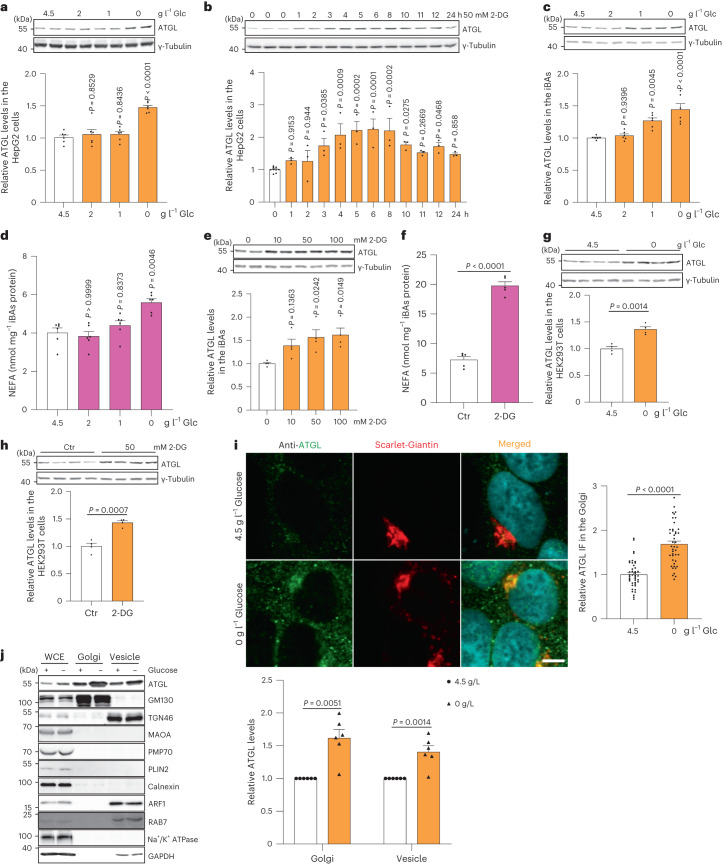
Glucose deprivation modulates lipolysis via stabilizing ATGL in the Golgi. **a**, Immunoblot of ATGL in HepG2 cells cultured at different glucose (Glc) concentrations for 6 h (*n* = 6 independent experiments per group). **b**, Immunoblot of ATGL in HepG2 cells treated with 50 mM 2-DG and collected at indicated timepoints (*n* = 9 or 3 independent experiments for control or 2-DG groups). **c**, Immunoblot of ATGL in iBAs cultured at different glucose concentrations for 24 h (*n* = 6 independent experiments per group). **d**, Levels of NEFAs in the starvation medium released by iBAs in the basal state 24 h after treatment with medium at different glucose concentrations (*n* = 6 independent experiments per group). **e**, Immunoblot of ATGL in iBAs treated with 2-DG at indicated concentrations for 24 h (*n* = 4 independent experiments per group). **f**, Levels of NEFAs in the starvation medium released by iBAs in the basal state 24 h after treatment with control vehicle (Ctr) and 100 mM 2-DG (*n* = 5 independent experiments per group). **g**, Immunoblot of ATGL in HEK293T cells 6 h after glucose withdrawal (*n* = 4 independent experiments per group). **h**, Immunoblot of ATGL in HEK293T cells 12 h after treatment with Ctr and 50 mM 2-DG (*n* = 4 independent experiments per group). **i**, Immunofluorescence of ATGL in HEK293-AAV cells 6 h after treatment with glucose free medium (*n* = 43 cells per group). Scale bar, 5 μm. **j**, Immunoblot of ATGL in the WCE, Golgi and vesicle fractions extracted from HEK293T cells 6 h after glucose withdrawal (*n* = 6 independent experiments per group). Results are shown as mean ± s.e.m. and analysed using two-tailed unpaired *t*-test (**f**–**i**), two-tailed paired *t*-test (**j**), one-way ANOVA method with Dunnett correction for multiple comparisons between control and other groups (**a**–**c** and **e**) and Kruskal–Wallis test with Dunn’s correction for multiple comparisons between control and other groups (**d**). Source numerical data and unprocessed blots are available in . Source data

**Extended Data Fig. 3:**
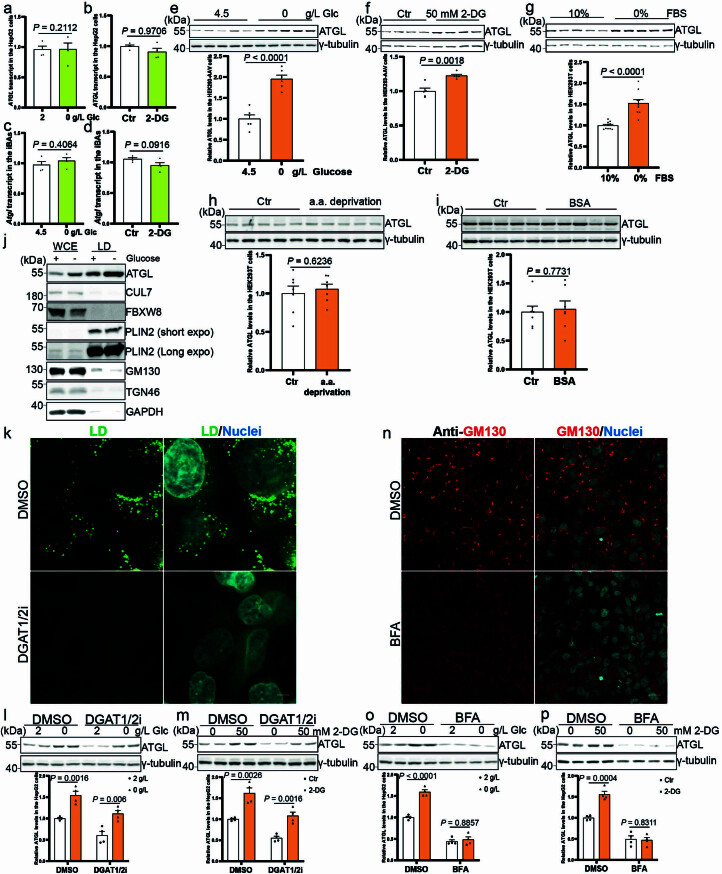
ATGL protein is stabilized in various cell types treated with glucose or serum deprivation. (**a-b**) Quantification of *ATGL* transcript levels in the HepG2 cells 6 h after glucose withdrawal or 50 mM 2-DG treatment (n = 4 independent experiments per group). (**c**, **d**) Quantification of *Atgl* transcript levels in the iBAs 24 h after glucose withdrawal and 100 mM 2-DG treatment (n = 4 independent experiments per group). (**e**) Immunoblot of ATGL in the HEK293-AAV cells 6 h after glucose withdrawal (n = 6 independent experiments per group). (**f**) Immunoblot of ATGL in the HEK293-AAV cells 12 h after treatment with 50 mM 2-DG (n = 5 independent experiments per group). (**g**) Immunoblot of ATGL in the HEK293T cells 6 h after serum withdrawal (n = 10 independent experiments per group). (**h**, **i**) Immunoblot of ATGL in the HEK293T cells 6 h after treatment with amino acid or FAs free medium (n = 7 independent experiments per group). (**j**) Immunoblot of ATGL in the WCE and LD fraction extracted from HepG2 cells 6 h after glucose withdrawal. Three times repeated independently with similar results. (**k**) Representative images of LDs in the HepG2 cells 48h after treatment with DGAT1/2 inhibitors and stained by BODIPY. Scale bar, 5 μm. Two times repeated independently with similar results. (**l, m**) Immunoblot of ATGL in the HepG2 cells 6 h after glucose withdrawal or 2-DG treatment in the absence of LDs (n = 4 independent experiments per group). (**n**) Representative images of Golgi in the HepG2 cells treated with BFA and immunostained with anti-GM130. Scale bar, 20 μm. Two times repeated independently with similar results. (**o**, **p**) Immunoblot of ATGL in the HepG2 cells 6 h after glucose withdrawal or 2-DG treatment in the absence of Golgi (n = 4 independent experiments per group). Results are shown as mean ± s.e.m. and analyzed using two-tailed unpaired *t*-test (a to i, l, m, o (DMSO) and p) and two-tailed Mann-Whitney test (o (BFA)). Source numerical data and unprocessed blots are available in source data. Source data

As ATGL was predominantly regulated at the protein levels, we focused on the mechanism of ATGL stabilization in response to glucose availability. In agreement with the in vivo data, we observed that both glucose withdrawal and 2-DG treatment strongly induced ATGL protein abundance without changing transcript levels in HepG2 cells (Fig. 2a,b and Extended Data Fig. 3a,b). This finding was first corroborated in immortalized brown adipocytes (iBAs) (Fig. 2c–f and Extended Data Fig. 3c,d). In addition, we observed upregulation of ATGL protein in HEK293T and HEK293-AAV cells upon glucose deprivation (Fig. 2g,h and Extended Data Fig. 3e,f). Moreover, when we removed serum, amino acids as well as FAs from the culture medium we observed that only serum deprivation promoted the accumulation of ATGL protein (Extended Data Fig. 3g–i).

After being synthesized in the ER, ATGL is transported to the Golgi, where it translocates to the LD surface via elusive mechanisms^27^. Inhibition of this process strongly reduces intracellular ATGL proteins levels, highlighting the importance of the Golgi–LD axis in modulating ATGL abundance and function^27,28^. Therefore, we focused on the LDs and Golgi as potential sites for the regulation of ATGL protein levels. First, we isolated LDs from HepG2 cells and observed that ATGL levels increased in the LDs fraction upon glucose deprivation (Extended Data Fig. 3j). Next, we treated HepG2 cells with inhibitors of DGAT1 and DGAT2, the key enzymes for the esterification of FAs, to deplete LDs (Extended Data Fig. 3k). Intriguingly, even in the absence of LDs, glucose deprivation could still increase endogenous ATGL levels (Extended Data Fig. 3l,m). Conversely, we did not observe any changes in intracellular ATGL levels upon glucose deprivation after treatment with Brefeldin A (BFA), a fungal toxin which blocks Golgi formation (Extended Data Fig. 3n–p). Therefore, we quantified ATGL levels in the Golgi by immunostaining and fractionation and observed significantly elevated protein levels in the Golgi and vesicle fractions in response to glucose deprivation (Fig. 2i,j). These data demonstrate that ATGL is primarily regulated in the Golgi and subsequently translocated to its functional site on the LD surface for lipolysis modulation.

### Reduction of Golgi PtdIns4P levels promotes ATGL stability

**Fig. 3:**
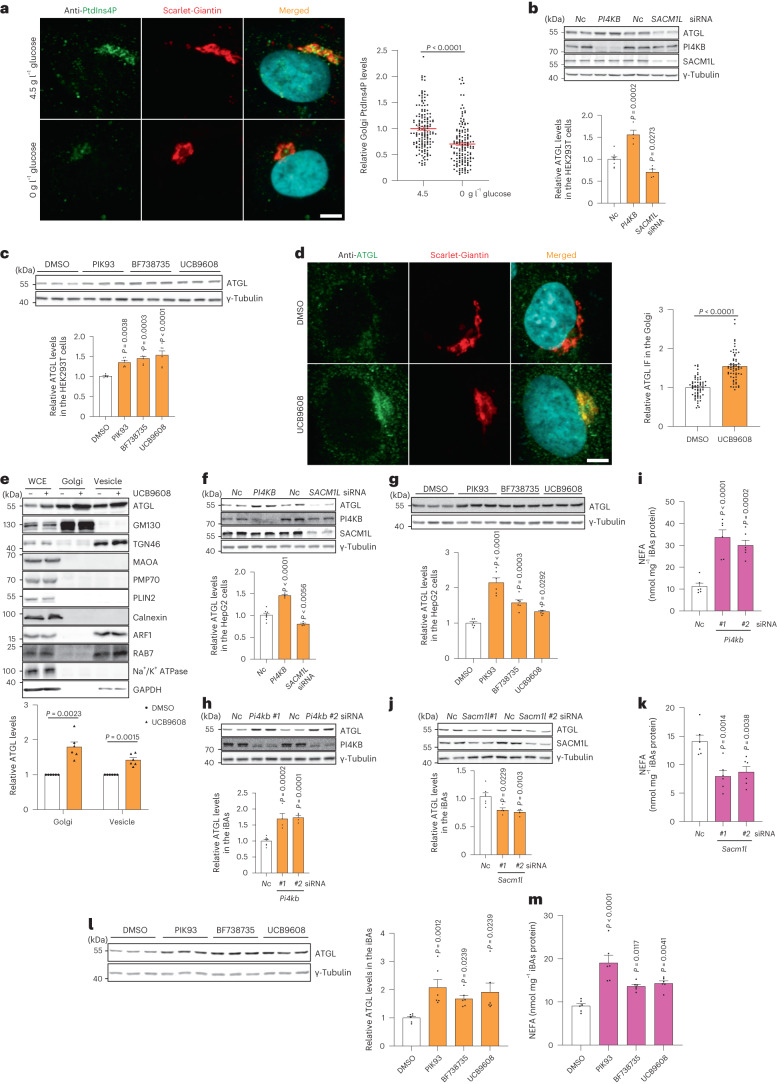
Golgi PtdIns4P regulates ATGL protein stability in the Golgi apparatus. **a**, Immunofluorescence of PtdIns4P in HEK293-AAV cells after glucose deprivation (*n* = 150 cells per group). Scale bar, 5 μm. **b**, Immunoblot of ATGL in HEK293T cells 72 h after *PI4KB* and *SACM1L* knockdown (*n* = 8 or 4 independent experiments for control or other groups). **c**, Immunoblot of ATGL in HEK293T cells 24 h after treatment with 5 μM PIK93, 10 μM BF738735 and 10 μM UCB9608 (*n* = 6 independent experiments per group). **d**, Immunofluorescence of ATGL in HEK293-AAV cells 24 h after UCB9608 treatment (*n* = 61 cells per group). Scale bar, 5 μm. **e**, Immunoblot of ATGL in the different fractions from HEK293T cells 6 h after UCB9608 treatment (*n* = 6 independent experiments per group). **f**, Immunoblot of ATGL in HepG2 cells 72 h after *PI4KB* and *SACM1L* knockdown (*n* = 8 or 4 independent experiments for control or other groups). **g**, Immunoblot of ATGL in HepG2 cells 24 h after treatment with PIK93, BF738735 and UCB9608 (*n* = 6 independent experiments per group). **h**,**i**, Immunoblot of ATGL and determination of basal lipolysis 72 h after knocking down *Pi4kb* in the iBAs (*n* = 8 or 4 independent experiments for control or other groups in **h**; *n* = 6 independent experiments per group in **i**). **j**,**k**, Immunoblot of ATGL and determination of basal lipolysis 72 h after knocking down *Sacm1l* in the iBAs (*n* = 6 or 4 independent experiments for control or other groups in **j**; *n* = 6 independent experiments per group in **k**). **l**,**m**, Immunoblot of ATGL (**l**) and determination of basal lipolysis (**m**) 24 h after treating iBAs with 1 μM PIK93, 1 μM BF738735 and 1 μM UCB9608 (*n* = 6 independent experiments per group). Data are presented as mean ± s.e.m. and analysed using two-tailed paired *t*-test (**e**), two-tailed Mann–Whitney test (**a** and **d**), one-way ANOVA method with Dunnett correction for multiple comparisons between control and other groups (**b**, **c**, **f**–**k** and **m**) and Kruskal–Wallis test with Dunn’s correction for multiple comparisons between control and other groups (**l**). Source numerical data and unprocessed blots are available in . Source data

**Extended Data Fig. 4:**
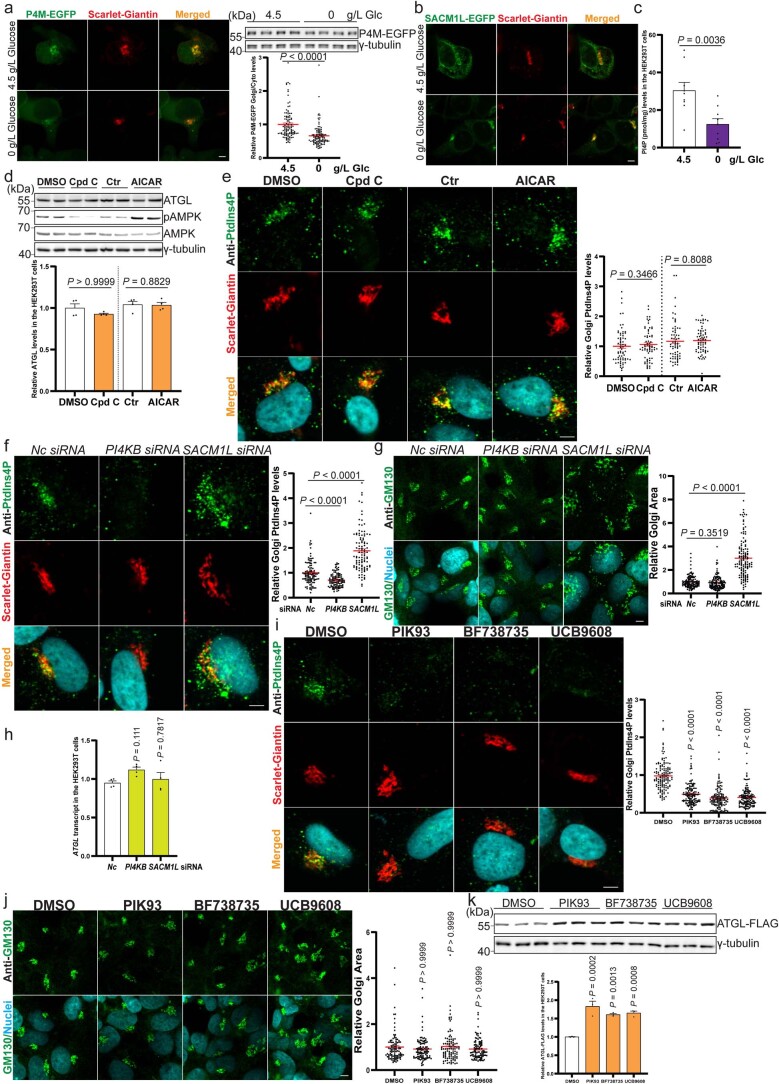
Golgi PtdIns4P regulates ATGL protein stability in the Golgi apparatus. (**a**) Representative images of P4M-EGFP signal in the HEK293-AAV cells 12 h after glucose deprivation (n = 107 cells per group). Scale bar, 5 μm. (**b**) Representative images of SACM1L-EGFP signal in the HEK293-AAV cells after glucose deprivation. Scale bar, 5 μm. Three times repeated independently with similar results. (**c**) Determination of total PtdIns4P levels in the HEK293T cells 24 h after glucose deprivation (n = 9 independent experiments per group). (**d**) Immunoblot of ATGL and pAMPK in the HEK293T cells after treatment with AMPK inhibitor Compound C (Cpd C) and activator AICAR (n = 4 independent experiments per group). (**e**) PtdIns4P quantification via immunostaining in the HEK293-AAV cells after treatment with Cpd C and AICAR (n = 67 cells per group). Scale bar, 5 μm. (**f–g**) Quantification of PtdIns4P levels and Golgi area via immunostaining in the HEK293-AAV cells 72 h after *PI4KB* and *SACM1L* knockdown (n = 103 cells per group in f; n = 130 cells per group in g). Scale bar, 5 μm. (**h**) Quantification of *ATGL* transcript in the HEK293T cells 72 h after *PI4KB* and *SACM1L* knockdown (n = 4 independent experiments per group). (**i–j**) Quantification of PtdIns4P levels and Golgi area via immunostaining in the HEK293-AAV cells after 24 h treatment with 5 μM PIK93, 10 μM BF738735 and 10 μM UCB9608 (n = 136 cells per group in i; n = 100 cells per group in j). Scale bar, 5 μm. (**k**) Immunoblot of ectopically expressed ATGL in the HEK293T cells 24 h after treatment with 5 μM PIK93, 10 μM BF738735 and 10 μM UCB9608 (n = 3 independent experiments per group). Results are shown as mean ± s.e.m. and analyzed using two-tailed unpaired *t*-test (c and d (Ctr and AICAR)), two-tailed Mann-Whitney test (a, d (DMSO and Cpd C) and e), one-way ANOVA method with Dunnett correction for multiple comparisons between control and other groups (h and k) and Kruskal-Wallis test with Dunn’s correction for multiple comparisons between control and other groups (f, g, i and j). Source numerical data and unprocessed blots are available in source data. Source data

**Extended Data Fig. 5:**
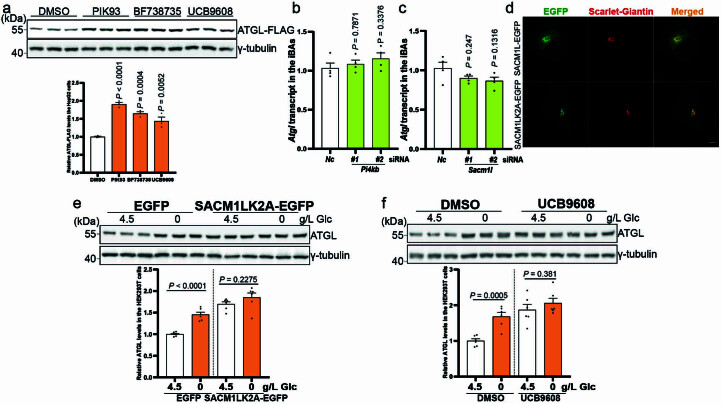
Glucose deprivation elevates ATGL protein in the Golgi via PtdIns4P regulation. (**a**) Immunoblot of ectopically expressed ATGL in the HepG2 cells 24 h after treatment with 5 μM PIK93, 10 μM BF738735 and 10 μM UCB9608 (n = 3 independent experiments per group). (**b**) Quantification of *Atgl* transcript levels in the iBAs 72 h after *Pi4kb* knockdown (n = 4 independent experiments per group). (**c**) Quantification of *Atgl* transcript levels in the iBAs 72 h after *Sacm1l* knockdown (n = 4 independent experiments per group). (**d**) Representative images of SACM1L-EGFP and SACM1LK2A-EGFP in the HEK293-AAV cells. Scale bar, 5 μm. Two times repeated independently with similar results. (**e**) Immunoblot of ATGL in the HEK293T cells 6 h after treatment with glucose free medium under the background of SACM1LK2A-EGFP overexpression (n = 6 independent experiments per group). (**f**) Immunoblot of ATGL in the HEK293T cells 6 h after glucose deprivation under the background of UCB9608 treatment (n = 6 independent experiments per group). Results are shown as mean ± s.e.m. and analyzed using two-tailed unpaired *t*-test (e and f) and one-way ANOVA method with Dunnett correction for multiple comparisons between control and other groups (a to c). Source numerical data and unprocessed blots are available in source data. Source data

Both glucose and serum deprivation have been reported to reduce Golgi PtdIns4P levels in various cell types, which is mainly mediated by promoting the retention of SACM1L in the Golgi after transfer from the endoplasmic reticulum (ER)^25,26^. Therefore, we hypothesized that reduction of Golgi PtdIns4P levels during nutrient shortage might stabilize ATGL and, in turn, promote lipolysis. Firstly, we examined Golgi PtdIns4P levels via a P4M–EGFP sensor and a PtdIns4P antibody^29^. We observed a reduction of Golgi PtdIns4P levels in conjunction with enrichment of SACM1L in the Golgi after glucose withdrawal (Fig. 3a and Extended Data Fig. 4a–c). The glucose deprivation reduced Golgi PtdIns4P levels independently of AMPK (Extended Data Fig. 4d,e). To study the effects of Golgi PtdIns4P on lipolysis, we genetically manipulated Golgi PtdIns4P levels via small interfering RNA (siRNA)-mediated knockdown of *PI4KB* and *SACM1L*^22^ (Extended Data Fig. 4f,g). Interestingly, we observed that Golgi PtdIns4P reduction led to elevated ATGL levels, whereas Golgi PtdIns4P accumulation decreased ATGL protein abundance (Fig. 3b and Extended Data Fig. 4h). Moreover, we used three PI4KB inhibitors to block Golgi PtdIns4P generation, and all three compounds elevated both endogenous and ectopically expressed ATGL protein levels (Fig. 3c and Extended Data Fig. 4i–k). As with glucose deprivation, ATGL protein accumulated in the Golgi when synthesis of Golgi PtdIns4P was blocked (Fig. 3d,e), indicating that Golgi PtdIns4P levels are crucial in determining the stability of ATGL. As glucose deprivation promoted ATGL-mediated lipolysis in different cell types, we examined the impact of Golgi PtdIns4P levels on this process in hepatocytes and adipocytes. We confirmed that the reduction of Golgi PtdIns4P increased both endogenous and ectopically expressed ATGL while upregulation had the opposite effect in hepatocytes (Fig. 3f,g and Extended Data Fig. 5a). Likewise, genetic depletion and pharmacological inhibition of PI4KB promoted ATGL stability and lipolysis whereas SACM1L depletion suppressed this process in adipocytes (Fig. 3h–m and Extended Data Fig. 5b,c).

Considering the relationship between glucose deprivation and Golgi PtdIns4P, as well as their roles in ATGL stabilization, we next studied whether glucose deprivation could regulate ATGL degradation via Golgi PtdIns4P. We first analysed ATGL regulation in response to glucose deprivation in the presence of SACM1LK2A overexpression (Extended Data Fig. 5d), a Golgi resident mutant of SACM1L to reduce Golgi PtdIns4P levels^26^. Furthermore, we depleted PtdIns4P in the Golgi by UCB9608-mediated inhibition of PI4KB. The loss of ATGL regulation after SACM1LK2A overexpression and UCB9608 treatment suggests that reduction of Golgi PtdIns4P levels is not only required for glucose deprivation induced ATGL upregulation, but also sufficient to increase ATGL levels even under high-glucose conditions (Extended Data Fig. 5e,f). Collectively, we could show that glucose availability regulates the distribution of SACM1L between ER and Golgi and modulates Golgi PtdIns4P levels, which in turn governs lipolysis by regulating ATGL stability in the Golgi.

### CUL7^FBXW8^ regulates ATGL in the Golgi apparatus

**Extended Data Fig. 6:**
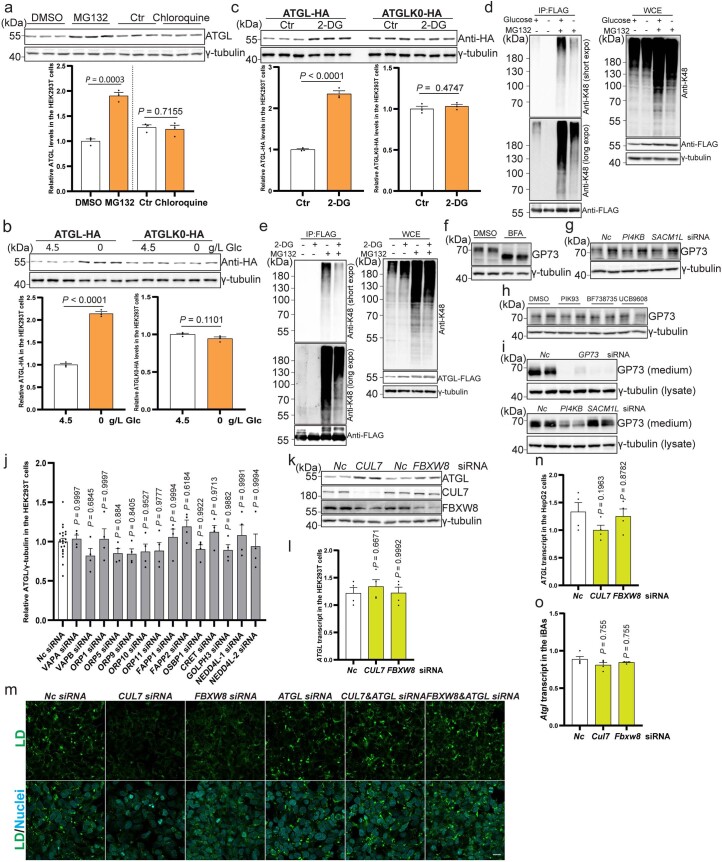
Glucose deprivation decreases ATGL poly-ubiquitylation via Golgi resident E3 ligase complex CUL7FBXW8. (**a**) Immunoblot of ATGL in the HEK293T cells 12 h after treatment with 10 μM MG132 and 100 μM Chloroquine (n = 3 independent experiments per group). (**b**, **c**) Immunoblot of ATGL-HA and ATGLK0-HA in the HEK293T cells after glucose withdrawal or 50 mM 2-DG treatment (n = 3 independent experiments per group). (**d**, **e**) K48-linkage polyubiquitylation pattern detection of HEK293T cells transfected by ATGL–FLAG and treated with glucose-free medium or 2-DG, followed by IP to enrich ATGL–FLAG. Three times repeated independently with similar results. (**f**, **i**) Immunoblot of GP73 in the WCE or secreted GP73 in the medium of HEK293-AAV cells after treatment with 5 μg/mL BFA, 5 μM PIK93, 10 μM BF738735, 10 μM UCB9608 or different siRNAs. Two times repeated independently with similar results. (**j**) Quantification of ATGL protein levels of HEK293T WCE in a small-scale siRNA screen via immunoblot (n = 22 or 4 independent experiments for control or other groups). (**k**) Immunoblot of ATGL in the HEK293T cells 72 h after *CUL7* and *FBXW8* knockdown. Four times repeated independently with similar results. (**l**) Quantification of *ATGL* transcript levels in the HEK293T cells 72 h after *CUL7* and *FBXW8* knockdown (n = 4 independent experiments per group). (**m**) Representative images of LDs stained via BODIPY in the HEK293-AAV cells 72 h after siRNA knockdown in the presence of 200 μM oleic acid. Scale bar, 20 μm. Three times repeated independently with similar results. (**n**) Quantification of *ATGL* transcript levels in the HepG2 cells 72 h after *CUL7* and *FBXW8* knockdown (n = 4 independent experiments per group). (**o**) Quantification of *Atgl* transcript levels in the iBAs 72 h after *Cul7* and *Fbxw8* knockdown (n = 4 independent experiments per group). Results are shown as mean ± s.e.m. and analyzed using two-tailed unpaired *t*-test (a to c), one-way ANOVA method with Dunnett correction for multiple comparisons between control and other groups (j, l and n) and Kruskal-Wallis test with Dunn’s correction for multiple comparisons between control and other groups (o). Source numerical data and unprocessed blots are available in source data. Source data

Although ATGL is regulated in the Golgi, it remains unclear how Golgi PtdIns4P levels could modulate ATGL stability. Given that proteasome-mediated degradation and autophagy are the main pathways to degrade intracellular proteins, we blocked both processes by MG132 and chloroquine treatment, respectively^30^. Interestingly, only MG132 treatment led to the cellular accumulation of ATGL protein (Extended Data Fig. 6a), indicating that ATGL degradation is executed by the proteasome. K48-linked polyubiquitylation of substrates acts as the main signal for proteasome-mediated degradation^30^. We thus examined whether lysine ubiquitylation of ATGL is dispensable for its regulation upon glucose deprivation. Therefore, we constructed a lysine null ATGL mutant^20^, and analysed its accumulation after glucose-free medium and 2-DG treatments (Extended Data Fig. 6b,c). In contrast to wild-type ATGL, the lysine null ATGL mutant was unresponsive to either treatment (Extended Data Fig. 6b,c). Furthermore, K48-linked polyubiquitylation of ATGL was reduced upon glucose deprivation (Extended Data Fig. 6d,e). These observations suggest the presence of a Golgi resident E3 ligase to sense glucose availability and polyubiquitylate ATGL for proteasome-mediated degradation.

**Fig. 4:**
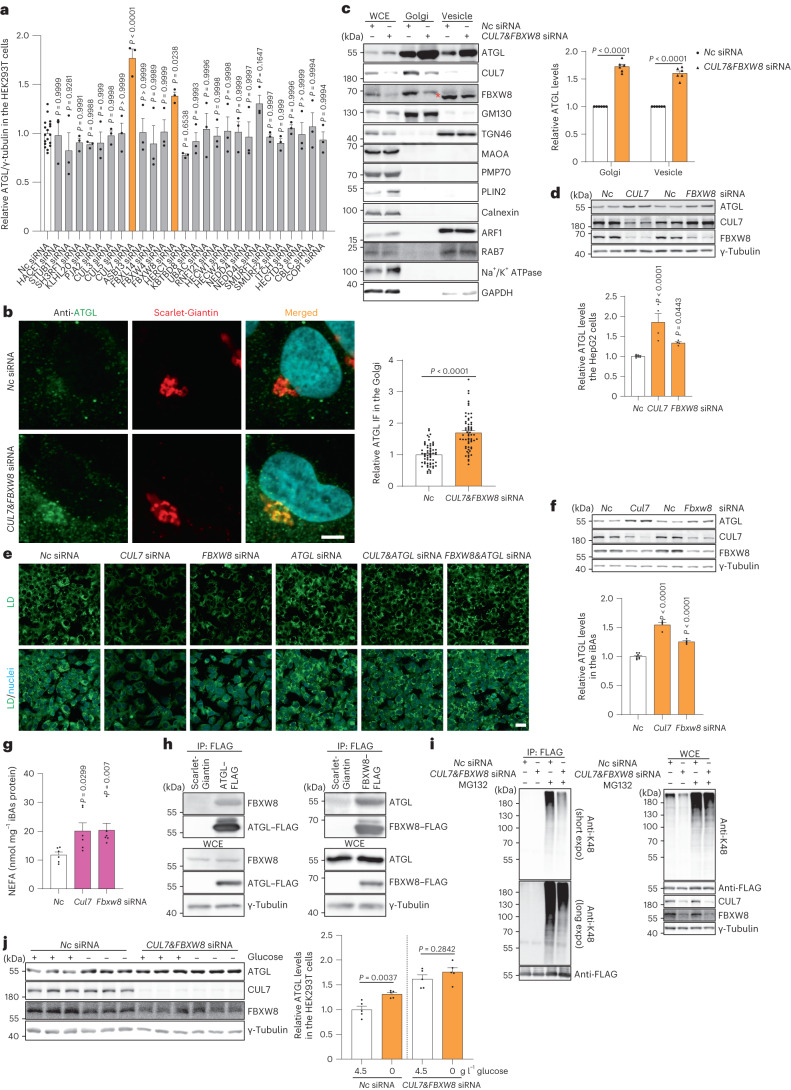
The Golgi resident E3 ligase complex CUL7^FBXW8^ polyubiquitylates ATGL for degradation. **a**, Quantification of ATGL protein levels in HEK293T WCE in a small-scale siRNA screen via immunoblot (*n* = 18 or 3 independent experiments for control or other groups). **b**, Immunofluorescence of ATGL in HEK293-AAV cells 72 h after *CUL7*&*FBXW8* knockdown (*n* = 61 cells per group). Scale bar, 5 μm. **c**, Immunoblot of ATGL in the WCE, Golgi and vesicle fractions extracted from HEK293T cells 72 h after *CUL7*&*FBXW8* knockdown (*n* = 6 independent experiments per group). Asterisk indicates a non-specific band. **d**, Immunoblot of ATGL in HepG2 cells 72 h after *CUL7* and *FBXW8* knockdown (*n* = 8 or 4 independent experiments for control or other groups). **e**, Representative images of LDs stained by BODIPY in HepG2 cells 72 h after siRNA knockdown. Scale bar, 20 μm. Three times repeated independently with similar results. **f**, Immunoblot of ATGL in iBAs 72 h after *Cul7* and *Fbxw8* knockdown (*n* = 8 or 4 independent experiments for control or other groups). **g**, Levels of NEFAs in the starvation medium released by iBAs in the basal state 72 h after knockdown of *Cul7* and *Fbxw8* (*n* = 6 independent experiments per group). **h**, Co-IP conducted in HEK293T WCE by FLAG antibody 48 h after expressing ATGL–FLAG or FBXW8–FLAG. Three times repeated independently with similar results. **i**, K48-linkage polyubiquitylation pattern detection in HEK293T cells transfected with ATGL–FLAG, followed by siRNA transfection for 72 h and IP to enrich ATGL–FLAG. Four times repeated independently with similar results. **j**, Immunoblot of ATGL in HEK293T cells 6 h after glucose withdrawal in the absence of CUL7^FBXW8^ (*n* = 5 independent experiments per group). Data are presented as mean ± s.e.m. and analysed using two-tailed unpaired *t*-test (**b** and **j**), two-tailed paired *t*-test (**c**), one-way ANOVA method with Dunnett correction for multiple comparisons between control and other groups (**a**, **d** and **f**) and Kruskal–Wallis test with Dunn’s correction for multiple comparisons between control and other groups (**g**). Source numerical data and unprocessed blots are available in . Source data

To identify the E3 ligase responsible for glucose deprivation-induced ATGL accumulation in the Golgi, we performed a small screen of E3 ligases, which are reportedly localized to the Golgi, and the well-known Golgi PtdIns4P effectors mediating the functions of PtdIns4P in Golgi organization, membrane trafficking and protein secretion (Extended Data Fig. 6f–i)^31–35^. Among them, we observed that only depletion of CUL7 and FBXW8, was able to significantly elevate ATGL levels in both the whole-cell extract (WCE) and the Golgi fraction (Fig. 4a–c and Extended Data Fig. 6j–l). Furthermore, depletion of either component substantially reduced LD levels, dependent on the presence of ATGL (Extended Data Fig. 6m). The scaffold protein CUL7 interacts with substrate receptor FBXW8 to form a functional E3 ligase complex^36,37^. Moreover, the presence of a positively charged polybasic region, a putative interactor of negatively charged PtdIns4P^23,24^, in the N-terminus of FBXW8 might allow the CUL7^FBXW8^ E3 ligase complex to sense glucose availability. Before testing this hypothesis, we first analysed the function of CUL7^FBXW8^ in hepatocytes and adipocytes. Depletion of CUL7^FBXW8^ resulted in higher ATGL protein levels, as well as an ATGL-dependent decline of LD abundance and lipolysis upregulation (Fig. 4d–g and Extended Data Fig. 6n,o). Therefore, we measured the physical interaction between FBXW8 and ATGL by co-immunoprecipitation (co-IP). We observed a direct interaction of FBXW8 and ATGL (Fig. 4h), as well as reduced K48-linked polyubiquitylation levels of ATGL, after CUL7^FBXW8^ depletion, which demonstrates that CUL7^FBXW8^ interacts with ATGL in the Golgi for polyubiquitylation and proteasome-mediated degradation (Fig. 4i). Lastly, we examined ATGL regulation upon glucose deprivation in the absence of CUL7^FBXW8^ and found that glucose sensing was abolished under this condition (Fig. 4j). Taken together, these data show that the Golgi resident E3 ligase complex CUL7^FBXW8^ is essential to regulate polyubiquitylation and degradation of ATGL in response to glucose deprivation.

### Golgi PtdIns4P Regulates CUL7^FBXW8^ function

**Fig. 5:**
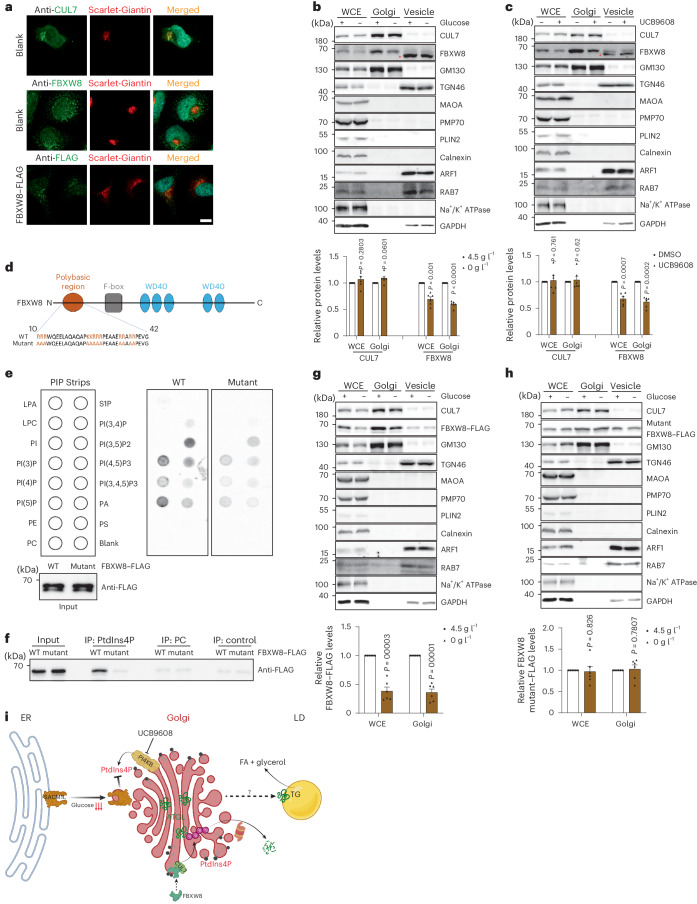
Recruitment of FBXW8 to the Golgi via interaction with PtdIns4P. **a**, Representative photomicrographs of the immunofluorescence of CUL7, FBXW8 and ectopically expressed FBXW8–FLAG in HEK293-AAV cells stained by the indicated antibodies. Scale bar, 5 μm. Repeated independently three times with similar results. **b**,**c**, Immunoblot of CUL7 and FBXW8 in the WCE, Golgi and vesicle fractions extracted from HEK293T cells after glucose withdrawal or UCB9608 treatment (*n* = 6 independent experiments per group). Asterisk indicates a non-specific band. **d**, Schematic illustration of the domains of human FBXW8. **e**, Blotting analysis of PIP strips incubated with recombinant proteins FBXW8–FLAG and FBXW8 mutant–FLAG. Three times repeated independently with similar results. **f**, Immunoblot of recombinant protein FBXW8–FLAG and FBXW8 mutant–FLAG after pull-down by PtdIns4P agarose beads. Three times repeated independently with similar results. **g**,**h**, Immunoblot of FBXW8–FLAG and FBXW8 mutant–FLAG in the WCE, Golgi and vesicle fractions extracted from HEK293T cells after glucose withdrawal (*n* = 6 independent experiments per group). **i**, Schematic diagram illustrating the working model of the mechanism by which glucose availability is coupled to lipolysis via Golgi PtdIns4P. Results are shown as mean ± s.e.m. and analysed using two-tailed paired *t*-test (**b**, **c**, **g** and **h**). Source numerical data and unprocessed blots are available in . Source data

**Extended Data Fig. 7:**
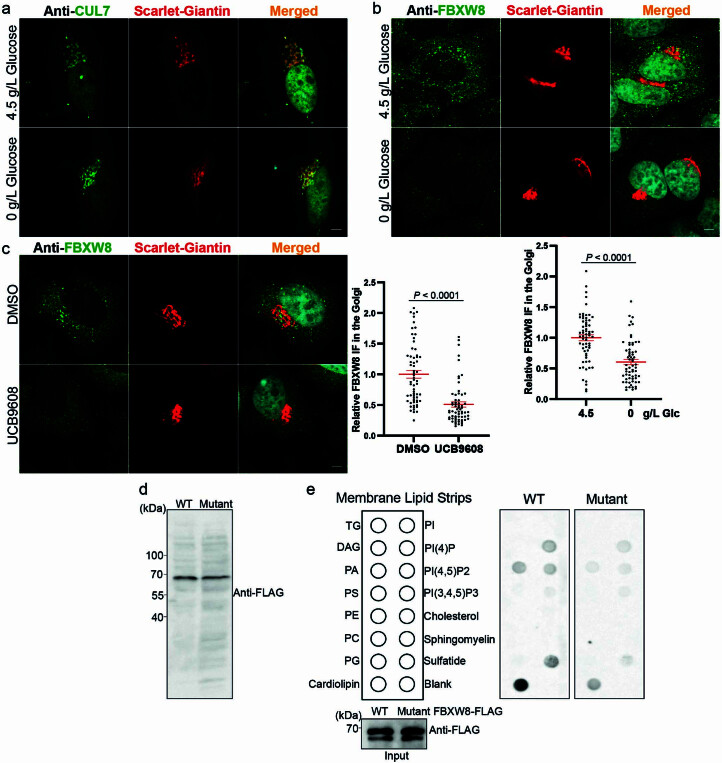
Recruitment of FBXW8 to the Golgi via interaction with PtdIns4P. (**a**) Representative immunofluorescent images of endogenous CUL7 in the HEK293-AAV cells after treatment with glucose free medium. Scale bar, 5 μm. Three times repeated independently with similar results. (**b**) Representative immunofluorescent images of endogenous FBXW8 in the HEK293-AAV cells after treatment with glucose free medium (n = 64 cells per group). Scale bar, 5 μm. (**c**) Representative immunofluorescent images of endogenous FBXW8 in the HEK293-AAV cells after treatment with UCB9608 (n = 64 cells per group). Scale bar, 5 μm. (**d**) Immunoblot of ectopically expressed protein in the HEK293T cells stably expressing FBXW8-FLAG and FBXW8 mutant-FLAG. Two times repeated independently with similar results. (**e**) Blotting analysis of Membrane Lipid Strips incubated with purified recombinant protein FBXW8-FLAG and FBXW8 mutant-FLAG. Three times repeated independently with similar results. Results are shown as mean ± s.e.m. and analyzed using two-tailed unpaired *t*-test (b) and two-tailed Mann-Whitney test (c). Source numerical data and unprocessed blots are available in source data. Source data

CUL7 and FBXW8 were previously shown to localize to the Golgi of certain cell types^32,38^. To understand how CUL7^FBXW8^ senses glucose availability and, in turn, regulates ATGL degradation, we next examined the cellular localization of CUL7 and FBXW8 by immunostaining. In line with previous reports and our fractionation result, endogenous CUL7 was highly enriched in the Golgi, instead of vesicles (Figs. 4c and 5a). Unexpectedly, however, both endogenously and ectopically expressed FBXW8 exhibit a dispersed pattern, with a certain portion of localization in the Golgi (Figs. 4c and 5a). In contrast, it was barely detectable in the vesicle fraction (Fig. 4c). Considering the existence of the polybasic region in the N terminus of FBXW8, we hypothesized that Golgi PtdIns4P might be able to recruit FBXW8 to the Golgi where it would be assembled into a functional E3 ligase complex. Therefore, we examined CUL7 and FBXW8 levels in the Golgi upon glucose deprivation via fractionation and immunostaining. As expected, FBXW8 instead of CUL7 displayed reduced levels in the Golgi (Fig. 5b and Extended Data Fig. 7a,b). Next, we depleted Golgi PtdIns4P, which led to reduced FBXW8 distribution to the Golgi (Fig. 5c and Extended Data Fig. 7c), indicating that FBXW8 distribution is dependent on Golgi PtdIns4P. To demonstrate the interaction between the polybasic region of FBXW8 and PtdIns4P, we built a FLAG-tagged wild-type FBXW8 and an FBXW8 mutant in which all lysine and arginine residues in the polybasic region of FBXW8 were mutated to alanine (Fig. 5d and Extended Data Fig. 7d). We purified recombinant FBXW8 proteins and examined their binding capacity with various lipid species using a membrane lipid strip. Among the tested lipid species, PtdIns4P displayed a binding capacity to wild-type FBXW8 whereas the interaction was lower between PtdIns4P and the mutant FBXW8 (Extended Data Fig. 7e). These data were further confirmed using PIP strips and PtdIns4P-conjugated agarose beads (Fig. 5e,f).

After demonstrating the physical interaction between the polybasic region of FBXW8 and PtdIns4P, we next examined whether the recruitment of FBXW8 to the Golgi was affected by glucose availability. In agreement with its PtdIns4P interaction capacity, wild-type FBXW8 levels in the Golgi were reduced upon glucose deprivation, while mutant FBXW8 was not sensitive to glucose levels (Fig. 5g,h). In conclusion, glucose deprivation leads to lower levels of Golgi PtdIns4P, which interacts with the polybasic region of FBXW8 for its recruitment to the Golgi, where the CUL7^FBXW8^ E3 ligase complex is assembled to polyubiquitylate ATGL for proteasomal degradation (Fig. 5i).

### Golgi PtdIns4P regulates ATGL-driven lipolysis in mice

**Fig. 6:**
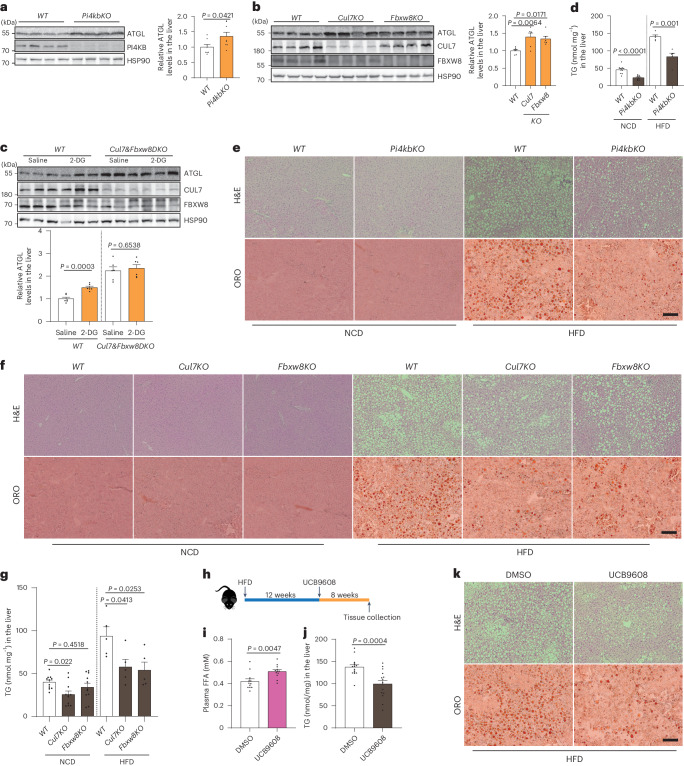
Regulation of lipolysis and MASLD development via manipulating Golgi PtdIns4P–CUL7^FBXW8^–ATGL axis. **a**, Immunoblot of ATGL protein in the liver from hepatocyte-specific *Pi4kb* knockout mice 2 weeks after AAV8 administration (*n* = 8 mice per group). **b**, Immunoblot of ATGL protein in the liver from hepatocyte-specific *Cul7* and *Fbxw8* knockout mice 2 weeks after AAV8 administration (*n* = 6 mice per group). **c**, Immunoblot of ATGL protein in the liver 1 h after 2-DG injection under the background of *Cul7* and *Fbxw8* double knockout in the hepatocytes (*n* = 6 mice per group). **d**, Determination of hepatic TG levels in NCD feeding mice 4 weeks after AAV8 administration or HFD feeding mice 8 weeks after AAV8 administration (*n* = 10 mice per NCD group and 5 mice per HFD group). **e**,**f**, H&E and ORO staining to the liver from hepatocyte-specific *Pi4kb* (**e**), *Cul7* and *Fbxw8* (**f**) knockout mice under NCD and HFD feeding conditions. Scale bar, 200 μm. Representative images from five mice per group with similar results. **g**, Determination of hepatic TG levels in hepatocyte-specific *Cul7* and *Fbxw8* knockout mice fed in NCD and HFD after AAV8 administration (*n* = 11 mice per NCD group and 5 mice per HFD group). **h**, Schematic illustration of experimental design to administer UCB9608 to wild-type mice. **i**, Plasma FFA levels in wild-type mice 8 weeks after administration of DMSO and UCB9608 (*n* = 12 mice per group). **j**, Hepatic TG levels in DMSO- and UCB9608-treated mice (*n* = 15 mice per group). **k**, H&E and ORO stainings to the liver from DMSO- and UCB9608-treated mice. Scale bar, 200 μm. Representative images from ten mice per group with similar results. Data are presented as mean ± s.e.m. and analysed using two-tailed unpaired *t*-test (**a**, **c**, **d** and **j**), two-tailed Mann–Whitney test (**i**) and one-way ANOVA method with Dunnett correction for multiple comparisons between control and other groups (**b** and **g**). Source numerical data and unprocessed blots are available in . Source data

**Extended Data Fig. 8:**
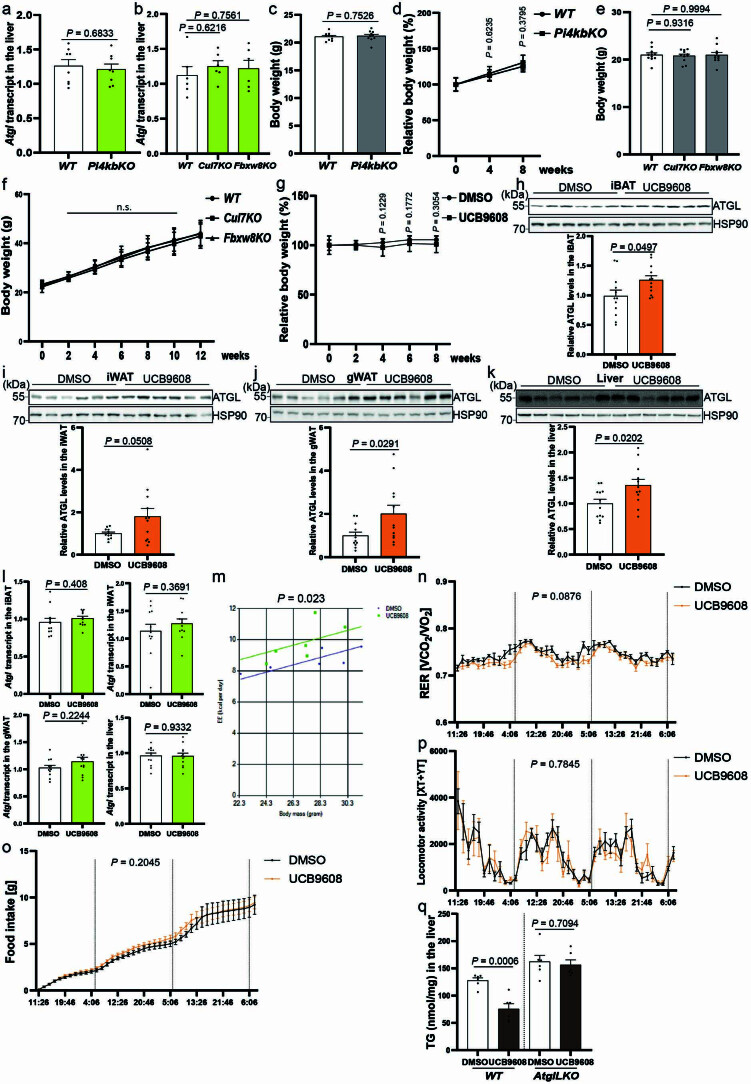
Regulation of ATGL-driven lipolysis and fatty liver development via manipulating Golgi PtdIns4P- CUL7FBXW8-ATGL axis. (**a**) Quantification of *Atgl* transcript levels in the liver from hepatocyte specific *Pi4kb* knockout mice (n = 8 mice per group). (**b**) Quantification of *Atgl* transcript in the liver from hepatocyte specific *Cul7* and *Fbxw8* knockout mice (n = 6 mice per group). (**c**) Body weight of hepatocyte specific *Pi4kb* knockout mice fed in NCD 4 weeks after AAV8 administration (n = 10 mice per group). (**d**) Body weight of hepatocyte specific *Pi4kb* knockout mice fed in HFD 8 weeks after AAV8 administration (n = 5 mice per group). (**e**) Body weight of hepatocyte specific *Cul7* and *Fbxw8* knockout mice fed in NCD 4 weeks after AAV8 administration (n = 11 mice per group). (**f**) Body weight of hepatocyte specific *Cul7* and *Fbxw8* knockout mice fed in HFD 12 weeks after AAV8 administration (n = 9 mice per group). (**g**) Body weight gain during the period of UCB9608 treatment (n = 10 mice per group). (**h–k**) Immunoblot of ATGL protein in the adipose depots and liver from DMSO- and UCB9608-treated mice (n = 12 mice per group). (**l**) Quantification of *Atgl* transcript levels in the adipose depots and liver from DMSO- and UCB9608-treated mice via qPCR (n = 12 mice per group). (**m–p**) Energy expenditure, Respiratory Exchange Ratio (RER), Food intake and Locomotor activity measurement of wild-type mice after 3 weeks administration of UCB9608 via HFD (n = 6 mice per group). (**q**) Hepatic TG levels in DMSO- and UCB9608-treated wild-type (*WT*) and liver specific *Atgl* knockout (*AtglLKO*) mice (n = 6 mice per group). Results are shown as mean ± s.e.m. (except d, f and g shown as mean ± s.d.) and analyzed using two-tailed unpaired *t*-test (a, c, d, g (4 and 6 weeks), h to l, and q), two-tailed Mann-Whitney test (g (8 weeks)), ANCOVA (m), one-way ANOVA method with Dunnett correction for multiple comparisons between control and other groups (b, e and f) and two-way ANOVA method (n-p). Source numerical data and unprocessed blots are available in source data. Source data

Our data identify Golgi PtdIns4P as the central player regulating ATGL-driven lipolysis, thus coupling glucose availability and intracellular FA liberation. Therefore, we focused on liver ATGL regulation as a key factor in hepatic lipolysis and TG accumulation, especially under conditions of MASLD pathogenesis^16,39^. To manipulate Golgi PtdIns4P levels in the liver, we administered *LSL-spCAS9* mice with guide RNAs (gRNAs) expressing AAV8 viruses to knock out *Pi4kb*, *Sacm1l*, *Cul7* or *Fbxw8* specifically in hepatocytes. This approach led to an efficient depletion of PI4KB protein levels in the liver 2 weeks after AAV8 administration (Fig. 6a). In the absence of PI4KB, ATGL protein levels were significantly upregulated (Fig. 6a and Extended Data Fig. 8a). Global depletion of *Sacm1l* has been reported to cause embryonic lethality^40^, and we found that liver-specific depletion of SACM1L was also lethal, which impedes any mechanistic investigation. Depletion of CUL7 led to a destabilization of FBXW8 in the liver but not vice versa (Fig. 6b). This is in contrast to previous reports demonstrating that these two factors can stabilize each other^36,37^. We observed increased ATGL levels after knockout of *Cul7* and *Fbxw8* in the liver (Fig. 6b and Extended Data Fig. 8b), indicating a consistent in vivo role for CUL7^FBXW8^. Next, we examined whether the E3 ligase complex CUL7^FBXW8^ is responsible for hepatic ATGL upregulation in response to glucose deprivation. Therefore, we administered 2-DG to *Cul7* and *Fbxw8* double knockout mice, which led to upregulation of hepatic ATGL levels of wild-type mice but not in the absence of CUL7^FBXW8^ (Fig. 6c), underscoring the essential role of CUL7^FBXW8^ in regulating glucose-deprivation-induced ATGL stabilization.

Upregulation of hepatic ATGL levels is reported to counteract TG deposition in the fatty liver^39^. Therefore, we examined the physiological and pathophysiological effects in MASLD model after genetic and pharmacological manipulation of the Golgi PtdIns4P–CUL7^FBXW8^–ATGL axis. Indeed, PI4KB depletion decreased hepatic TG levels (Fig. 6d,e and Extended Data Fig. 8c,d). Moreover, when PI4KB was ablated in the MASLD mouse model induced with 12-week high-fat diet (HFD), TG deposition in the fatty liver was significantly reduced 8 weeks after virus administration (Fig. 6d,e and Extended Data Fig. 8c,d). Similarly, we determined hepatic TG levels in the *Cul7* and *Fbxw8* knockout mice under normal chow diet (NCD) feeding conditions and found that only CUL7 ablation reduced hepatic TG levels (Fig. 6f,g and Extended Data Fig. 8e,f). In contrast, depletion of either factor was able to reduce hepatic TG accumulation in mice challenged with HFD for 12 weeks (Fig. 6f,g and Extended Data Fig. 8e,f). In addition to genetic manipulation, we examined the effect of the PI4KB inhibitor on lipolysis regulation and MASLD progression. Therefore, wild-type mice were fed on HFD for 12 weeks to induce hepatic steatosis, and subsequently treated with UCB9608 (ref. ^41^) (Fig. 6h). PI4KB inhibition significantly increased plasma FFA levels (Fig. 6i and Extended Data Fig. 8g), suggesting a consistent in vivo role of Golgi PtdIns4P in regulating ATGL-driven lipolysis in the adipose tissue. Therefore, we compared the ATGL protein levels in different adipose depots and livers from dimethyl sulfoxide (DMSO)- and UCB9608-treated mice, and we found the latter displayed significantly greater ATGL protein levels in the iBAT, gWAT and liver with a similar trend in the iWAT (Extended Data Fig. 8h–l). In agreement with the reported role of ATGL upregulation in the adipose tissue^42^, UCB9608 treatment led to higher energy expenditure, possibly due to elevated FA levels that are used as a substrate for β-oxidation (Extended Data Fig. 8m–p). The enhancement of ATGL-driven lipolysis in the adipose tissue might contribute to increased FA liberation and release, whereas elevation of hepatic ATGL levels could drive intrahepatic lipolysis and reduce hepatic TG levels^8,39^. Therefore, we determined hepatic TG levels in the livers of UCB9608-treated mice and observed substantially attenuated hepatic steatosis, dependent on the presence of liver ATGL (Fig. 6j,k and Extended Data Fig. 8q). In conclusion, we demonstrate that the Golgi PtdIns4P–CUL7^FBXW8^–ATGL axis regulates hepatic ATGL levels and LD clearance, which could provide a potential target to ameliorate hepatic steatosis.

### Reduction of Golgi PtdIns4P attenuates MASH progression

**Fig. 7:**
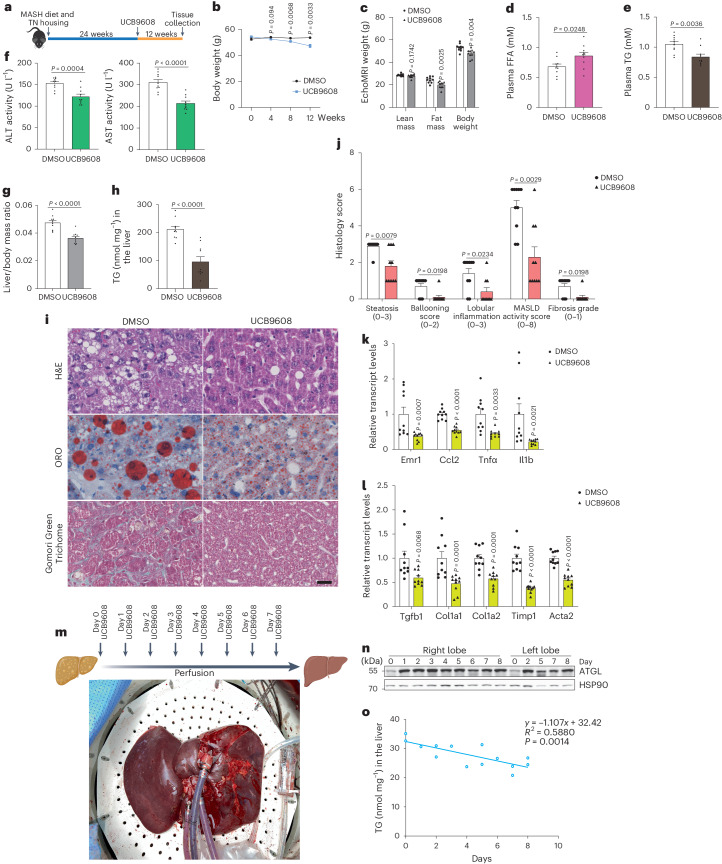
Amelioration of murine MASH progression and steatosis in human liver graft via blocking Golgi PtdIns4P generation. **a**, Schematic illustration of experiment design to administer UCB9608 to wild-type mice after induction of MASH via HFD, high-glucose/fructose-containing water and thermoneutral housing. **b**, BW gain during the period of UCB9608 treatment (*n* = 10 mice per group). **c**, EchoMRI analyses of lean mass, fat mass and BW to the MASH mice (*n* = 10 mice per group). **d**,**e**, Plasma FFA levels (**d**) and TG levels (**e**) 12 weeks after administration of UCB9608 (*n* = 10 mice per group). **f**, Alanine transaminase (ALT) and aspartate aminotransferase (AST) activity in the plasma from MASH mice after administration of UCB9608 (*n* = 10 mice per group). **g**, Liver mass/BW ratio of MASH mice after administration of UCB9608 (*n* = 10 mice per group). **h**, Determination of hepatic TG levels of MASH mice after administration of UCB9608 (*n* = 10 mice per group). **i**, Representative images of H&E staining, ORO staining and Gomori Green Trichome staining. Scale bar, 25 μm. Representative images from ten mice per group with similar results. **j**, Histology score of livers from MASH mice treated by DMSO and UCB9608 (*n* = 10 mice per group). **k**,**l**, Transcript levels of inflammatory markers (**k**) and fibrosis markers (**l**) in MASH livers as determined by qPCR (*n* = 10 mice per group). **m**, Schematic illustration of experiment design to administer UCB9608 to a human liver graft ex vivo during normothermic machine perfusion. **n**,**o**, Immunoblot of ATGL protein and quantification of TG levels in the human liver biopsies taken at different timepoints from both lobes. Three times repeated technically with similar results. Results are shown as mean ± s.e.m. and analysed using two-tailed unpaired *t*-test (**b**–**g** and **k** (Ccl2 and Tnfα) and l (Col1a2, Timp1 and Acta2)), two-tailed Mann–Whitney test (**h**, **j** and **k** (Emr1and Il1b) and **l** (Tgfb1 and Col1a1)) and two-tailed Pearson’s correlation (**o**). Source numerical data and unprocessed blots are available in . Source data

**Extended Data Fig. 9:**
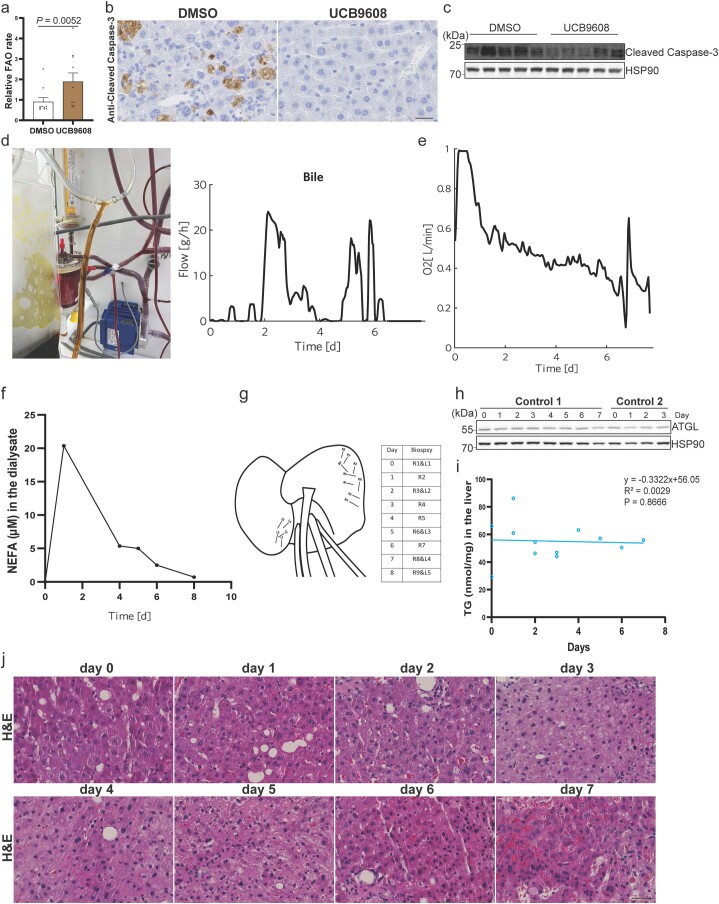
Amelioration of murine MASH progression and steatosis in human liver graft via blocking Golgi PtdIns4P generation. (**a**) Measurement of liver fatty acid oxidation using ^14^C-palmitate as the substrate (n = 10 mice per group). (**b**) Immunohistological analyses of liver apoptosis in the MASH mice via cleaved Caspase-3 antibody. Scale bar, 25 μm. Representative images from 10 mice per group with similar results. (**c**) Immunoblot of cleaved Caspase-3 in the adipose tissue from MASH mice. Two times repeated independently with similar results. (**d**) Bile acid excretion from the liver graft during 8 days of perfusion. (**e**) Oxygen consumption rate of the liver graft treated with UCB9608 over the course of perfusion. One liver graft used for perfusion. (**f**) Measurement of NEFA levels in the dialysate. Three times repeated technically with similar results. (**g**) Time points and locations of punch biopsies from the liver graft. (**h, i**) Immunoblot of ATGL protein and quantification of TG levels in the human liver biopsies taken at different time points from control grafts. Three times repeated technically with similar results. (**j**) H&E stainings of biopsies taken at different time points. Scale bar, 50 μm. One liver piece at every time point. Data were presented as mean ± s.e.m. and analyzed using two-tailed Mann-Whitney test (a) and two-tailed Pearson’s correlation (i). Source numerical data and unprocessed blots are available in source data. Source data

As we have demonstrated the potential applicability of the identified pathway for MASLD treatment, we further investigated whether targeting Golgi PtdIns4P production could attenuate MASH progression. To model MASH in the mouse liver^43^, wild-type mice were housed under thermoneutral conditions on HFD diet plus high glucose- and fructose-containing drinking water for 24 weeks and were afterwards treated with UCB9608 for another 12 weeks (Fig. 7a). Interestingly, we observed that UCB9608 treatment induced a significant loss of body weight (BW) and fat mass, in agreement with the enhanced lipolysis and energy expenditure induced by UCB9608 (Fig. 7b–d and Extended Data Fig. 8m). Hence, we further examined TG levels as well as markers of hepatic toxicity. The reduction of all parameters upon UCB9608 treatment indicated amelioration of hepatic steatosis and injury, leading us to perform pathohistological analyses (Fig. 7e,f). We observed that UCB9608 treatment reduced liver mass/BW ratio and hepatic TG levels, in conjunction with elevated FA oxidation (Fig. 7g,h and Extended Data Fig. 9a). Consistently, steatosis grade, liver injury, inflammation levels, fibrosis grade and MASLD activity score were significantly lower in the livers from UCB9608-treated mice, suggesting suppressed MASH progression, although it should be noted that the MASH induction in these murine livers was relatively mild compared to clinical MASH in human patients (Fig. 7i,j and Extended Data Fig. 9b,c). The mitigation of inflammation and fibrosis markers upon UCB9608 treatment was further corroborated by quantifying transcript levels (Fig. 7k,l).

The progression of MASH may lead to cirrhosis and possibly hepatocellular carcinoma, and both pathological situations require liver transplantation to circumvent liver failure. Liver grafts cannot be transplanted when they are steatotic, thus requiring potential strategies to defatten steatotic livers for clinical use. Given the ability of UCB9608 to ameliorate hepatic TG levels in mice, we tested the defattening effect of UCB9608 on a steatotic human liver during an ex vivo perfusion^44^. We utilized a human liver graft with 15% macrovesicular steatosis and perfused the liver with blood for 8 days. UCB9608 was administered daily to reach a target concentration of 10 μM (Fig. 7m). The graft displayed normal bile acid excretion and oxygen consumption, suggesting healthy status of the graft during the perfusion (Extended Data Fig. 9d,e). Interestingly, we detected non-esterified fatty acids (NEFAs) from dialysate during perfusion, suggesting an induction of lipolysis that resulted in the release of FAs (Extended Data Fig. 9f). To study the time course of liver defattening, we sampled biopsies from both lobes to determine ATGL and TG levels (Extended Data Fig. 9g). Consistent with the findings in mice, we observed substantially elevated ATGL levels after UCB9608 treatment in conjunction with reduced TG deposition while ATGL and hepatic TG levels remained unchanged in two control grafts, indicating a defattening effect mediated by ATGL-driven lipolysis upon UCB9608 administration (Fig. 7n,o and Extended Data Fig. 9h–j). Taken together, the discovery of Golgi PtdIns4P-mediated ATGL stabilization in response to glucose deprivation might be a promising therapeutic strategy to counteract MASH progression and might also serve as a potential therapeutic approach to repair steatotic liver grafts, ex vivo.

## Discussion

It is well known that the endocrine system plays important roles in glucose and FA handling during different nutritional states. Insulin promotes de novo lipogenesis to produce FAs using excess glucose as a carbon source, and at the same time it suppresses lipolysis to reduce FA release from lipid stores^6,7^. Conversely, glucagon or norepinephrine are secreted in response to starvation or cold exposure, to boost lipolysis through ATGL and HSL phosphorylation in hepatocytes and adipocytes^1,18^. Typically, such extracellular hormones rapidly initiate a systemic response to acute physiological alterations. However, hormone signalling usually does not act chronically due to corresponding receptor desensitization. This process is exemplified by classical β-arrestin-mediated desensitization of β1 adrenergic receptors upon ligand binding^45,46^, which might explain our results that elevated pHSL levels were reversed in the adipose tissue upon long-term glucose deprivation. This homeostatic feedback loop probably necessitated cells to develop intrinsic mechanisms to tune lipolysis to nutrient supplies as a complementary process to extracellular hormone-mediated regulation of lipid metabolism for the organism to meet complex physiological demands. The mechanism described here might reshape our understanding regarding the exact contribution of insulin signalling to lipolysis regulation as insulin could also modulate intracellular glucose levels to signal via the Golgi PtdIns4P–CUL7^FBXW8^–ATGL axis for lipolysis regulation. Reduction of HSL phosphorylation is believed to be the main mediator of the anti-lipolytic role of insulin^10,11^. Here we found that insulin does not suppress pHSL under conditions of glucose deprivation, suggesting that glucose itself is an important mediator of lipolytic function and possibly mediates the anti-lipolytic effect of insulin. This might be especially relevant in insulin resistance as we found that patients with type 2 diabetes display increased ATGL levels upon fasting. It should be noted that the lack of precise glucose uptake data during EHC makes it difficult to detangle the effects mediated either by insulin or glucose availability. Notably, insulin receptor substrate 1 (IRS1), a critical mediator of insulin signalling, is reported to be a substrate of CUL7^FBXW8^, supporting a crosstalk between insulin and Golgi PtdIns4P-mediated regulation of lipolysis^47^.

Although the electrostatic interaction between FBXW8 and negatively charged phospholipids is not specific to PtdIns4P as demonstrated by our in vitro assays, the recruitment of FBXW8 to the Golgi could be facilitated by enriched PtdIns4P levels in the Golgi as subcellular compartments have unique phospholipid distribution patterns^22^. Previous reports have shown that CUL7^FBXW8^ assembly promotes the stability of CUL7 and FBXW8 as loss of either factor strongly reduces the protein levels of the other^36,37^, and we found CUL7 depletion leads to destabilization of FBXW8. Consequently, FBXW8 levels in both Golgi and WCE are reduced since free FBXW8 is less stable in the cytosol. In contrast, we found that depletion of FBXW8 could only destabilize CUL7 in iBAs, suggesting that CUL7 is the major factor in stabilizing the E3 ligase complex. In line with this is our finding that CUL7 depletion has a stronger effect on ATGL upregulation. Although numerous E3 ligases have been reported to exist in the Golgi^32^, the sensitivity of the Golgi PtdIns4P–CUL7^FBXW8^–ATGL axis to glucose availability identifies the CUL7^FBXW8^ complex as the key E3 ligase that mediates glucose deprivation-induced metabolic rewiring through polyubiquitylation of other substrates in addition to IRS1 and ATGL, which will require further studies.

In contrast to adipocytes, most non-adipocytes possess fewer LDs, and thus upon glucose or serum deprivation they have to initiate autophagic degradation of non-essential cellular components to provide carbon sources to generate LDs in proximity to mitochondria^48^. The regulation of ATGL probably primes lipolysis activation in the starved cells for ATGL translocation from the Golgi to the LDs once the latter are generated. Although starvation elevates ATGL in the Golgi, vesicles and LDs, the regulation mainly takes place in the early Golgi, instead of the vesicles and LDs (Fig. 4c and Extended Data Fig. 3j). Moreover, whether and how Golgi derived vesicles and other types of intracellular vesicle mediate ATGL regulation as well as its translocation from Golgi to the LDs surface requires further investigations (Fig. 5i). In addition, abundant LDs can be artificially induced by oleic acid (OA)-containing medium in non-adipocytes. Under this condition, glucose or serum starvation can induce autophagic degradation of the LD coat proteins perilipin 2 and 3 (PLIN2/PLIN3), the clearance of which can empty the LD surface for ATGL localization and action^49^. We observed that PLIN1 was not altered in adipose depots after fasting, suggesting that the regulation of lipolysis in adipocytes and non-adipocytes are distinctive features despite the existence of a common regulatory pathway.

Hepatosteatosis is mainly due to elevated FAs delivered from the circulation, in addition to elevated hepatic de novo lipogenesis, decreased hepatic lipolysis and FA oxidation in the hepatocytes^50,51^. Inflammation is induced by secretion of inflammatory factors from the Kupffer cells while hepatic stellate cells are responsible for secretion of collagen that contributes to fibrosis^50^. Based on our findings and other reported studies, Golgi PtdIns4P might serve as a potent target to block MASH progression as Golgi PtdIns4P also plays pivotal roles in NLRP3 activation via direct recruitment to the Golgi and thus the inflammation in the MASH liver probably can be directly alleviated by lowering the PtdIns4P levels^23,52^. Further, procollagen, after being synthesized in the ER, traffics to the Golgi for secretion into the extracellular space. The formation of these vesicles might require the contribution of Golgi PtdIns4P to induce membrane curvature^22,53^. If so, targeting PI4KB might be a very promising strategy to directly attenuate steatosis, inflammation and fibrosis in MASH. More importantly, UCB9608 treatment reduced plasma TG levels in the mouse MASH model, unlike elevated TG secretion and hypertriglyceridaemia observed upon administration of an acetyl-CoA carboxylase inhibitor in mice and humans^54^. Considering that many PI4KB inhibitors have been developed to treat infectious diseases and cancer, such drugs could be repurposed to also treat MASH^22^. Interestingly, besides the defattening effect, UCB9608 is also known to exhibit immunosuppressive properties^41^. Considering immunosuppressive agents are necessary to prevent allograft rejection after organ transplantation, it will be a promising option to continue UCB9608 administration after engraftment if it is approved for clinical application.

In summary, our findings identify an intrinsic pathway that links glucose availability to lipid availability via glucose sensing by a Golgi PtdIns4P–ATGL–lipolysis pathway that leads to the upregulation of lipolysis and the increased release of FAs from LD stores upon glucose depletion. By such means, cells can fine-tune nutrient flux in addition to paracrine hormone-mediated regulation of nutrient availability.

## Methods

Human adipose tissues were obtained from EHC study, which was approved by the by the Local Ethics Committee (Bratislava, Slovakia) and conforms to the ethical guidelines of the 2000 Helsinki declaration. The discarded human liver grafts were from all Swiss transplant centres, which was approved by local authorities (Kantonale Ethik Kommission Zürich KEK Nr. 2017-000412). All animal experiments were approved by the Veterinary office of the Canton of Zurich or Institutional Animal Care and Use Committee of Sun Yat-sen University.

### EHC study

All study participants provided witnessed written informed consent before entering the study. Briefly, human insulin (Actrapid 100 IU ml^−1^, Novo Nordisk) was infused into the antecubital vein in a primed continuous fashion at the dose of 1 mU kg^−1^ min^−1^. Blood glucose was measured in the samples from the contralateral antecubital vein in 5 min intervals and maintained at euglycaemia (5.0 ± 0.25 mM) using variable infusion rate of 20% glucose. The whole-body insulin sensitivity (*M* value) was calculated from the steady-state plasma glucose infusion rate required to maintain euglycaemia and expressed per kg BW per minute, normalized to the steady-state insulinemia. Subcutaneous AT samples were taken by needle biopsy from abdominal region in the basal fasted state as well as during the steady state of EHC. Twenty-four middle-aged sedentary men (age 35.5 ± 1.5 years, body mass index 28.9 ± 1.1 kg m^−2^, adiposity 25.7 ± 1.6%) were assigned to insulin-sensitive (*n* = 16, *M* value 0.14 ± 0.02 mg per kg BW per minute per μU insulin per millilitre) and insulin-resistant (*n* = 8, *M* value 0.04 ± 0.01 mg per kg BW per minute per μU insulin per millilitre) subgroups according to their insulin sensitivity index.

### Human liver perfusion

The graft had an initial weight of 2,740 g (with gallbladder) before perfusion and was diagnosed with 15% macrovesicular steatosis by the pathological department of the University Hospital Zürich. The liver was procured with a total ischaemia time of 40 min after circulatory death of the donor. It was flushed with IGL-1 solution and perfused for 2 h with Belzer MPS at 8 °C. The infrahepatic vena cava was closed with a suture, and the hepatic artery, portal vein, suprahepatic vena cava and common bile duct were cannulated with steel cannulas. The graft was subsequently perfused with whole blood and a custom built normothermic perfusion machine^55^. Hepatic arterial pressure was controlled to 80/50 mmHg, allowing for an average flow of 0.25 l min^−1^. Epoprostenol was automatically infused to keep arterial flow stable. Oxygenation was automatically controlled with a setpoint partial pressure of oxygen equal to 12 kPa. Portal venous flow was controlled to 1 l min^−1^, resulting in a pressure of 2 mmHg on average. Further, oxygenation of portal blood was automatically regulated to achieve 65% saturation in the vena cava. Use of an electric pinch valve further allowed for pressure regulation in the vena cava, keeping a constant pressure of 0 mmHg. Glucose levels were automatically controlled by using a real-time glucose sensor (CCIT) and automated insulin infusions, keeping glucose levels at 7 mmol l^−1^ from the second day of perfusion onwards. A dialysis bypass was used to clear waste metabolites and control haematocrit levels by using a fixed dialysate influx rate of 200 ml h^−1^ and an automatically controlled outflux. After 8 days of perfusion, the liver weight decreased to 1,507 g (without gallbladder) and was sent for pathological examination. The liver graft finally displayed less than 5% macrovesicular steatosis determined by the pathological department of the University Hospital Zürich. Further, two control livers diagnosed with 30% and 50% macrovesicular steatosis grades were perfused for 69 h and 158 h, respectively. Both livers were perfused with the same perfusion device and perfusion protocol but did not receive a daily bolus of UCB9608.

### Mouse experiments

Both female and male mice were used in the experiments starting at the age of 8–10 weeks and they were housed two to five littermates per cage in ventilated cages at standard housing conditions (40% humidity, 22 °C, 12 h reversed light/dark cycle, dark phase starting at 7:00) with NCD (18% protein, 4.5% fibre, 4.5% fat, 6.3% ash, Provimi Kliba SA) and water. C57BL/6N male mice (Charles River Laboratories) were fasted for 24 h or injected with 2-DG (2 g kg^−1^ BW) for different durations. Female *ROSA26-LSL-spCas9* mice (#024857, JAX) were intravenously injected with various virus pools to express gRNA pools and CAS9 at a dose of 3 × 10^11^ genome copies per mouse. Two or four weeks later, livers were collected after 6 h fasting for protein and RNA extraction or TG determination, respectively. Also 4 weeks later, the liver-specific *Cul7&Fbxw8* double knockout mice were injected with 2-DG to induce 1 h glucose deprivation before dissection.

To study CUL7 and FBXW8 in MASLD model, male *ROSA26-LSL-spCas9* mice were injected with virus pools and fed a HFD (#3436, Provimi Kliba SA) for 12 weeks, followed by dissection after 6 h fasting. To study PI4KB in MASLD model, male *ROSA26-LSL-spCas9* mice were fed in HFD for 12 weeks before induction of PI4KB depletion. Mice were fed a HFD for another 8 weeks and dissected after 6 h fasting. Likewise, male C57BL/6N wild-type mice and *AtglLKO* were challenged with HFD for 12 weeks before administration of UCB9608 mixed in HFD (5 mg kg^−1^) for another 8 weeks. Then the mice were dissected after 6 h fasting.

*Atgl* floxed mice (#024278, JAX) were crossed with *Albumin-Cre* (#035593, JAX) and *Adip-CreERT2* strains to obtain *Alb-Atgl*^*fl/fl*^ and *AdipcreERT2-Atgl*^*fl/fl*^ mice^56^, which deplete ATGL protein in the liver and adipocytes (induced by tamoxifen), respectively. Male mice from both strains were injected with 2-DG to induce glucose deprivation for 6 h before dissection.

### 2-DG and insulin administration

Wild type C57BL/6N mice were intraperitoneally injected with saline or 2-DG for 5.5 h treatment, followed by insulin treatment (Actrapid, human insulin at dose of 0.75 U kg^−1^ BW) for 30 min. The mice were then dissected to study the acute anti-lipolytic effect of insulin under the background of glucose deprivation. To study the long-term anti-lipolytic effect of insulin during glucose deprivation, mice were co-administered with 2-DG and insulin for 6 h treatment before dissection.

### UCB9608 administration in murine MASH model

To model MASH in murine liver, male C57BL/6N mice were challenged with HFD for 24 weeks in conjunction with 18.9 g l^−1^ glucose&23.1 g l^−1^ fructose-containing drinking water under thermoneutral housing condition (29 °C) before the administration of UCB9608 via diet (15 mg kg^−1^) for 12 weeks. Then the mice were analysed in EchoMRI for body composition measurement before 6 h fasting, followed by dissection.

### Indirect calorimetry

C57BL/6N male mice were fed on HFD mixing with UCB9608 (5 mg kg^−1^) for 3 weeks before indirect calorimetry measurements were performed with Phenomaster (TSE systems) according to the manufacturer’s instructions. The energy expenditure between both groups was analysed via the analysis of covariance method (https://www.mmpc.org/shared/regression.aspx)^57^.

### Cell culture

iBAs were cultured as described before^58^. Briefly, iBAs were cultured on collagen-coated plates in Dulbecco’s modified Eagle medium (DMEM, high glucose) with 10% foetal bovine serum (FBS) plus 1% Pen/Strep. Once pre-adipocytes reached confluence, adipogenesis was induced by differentiation medium containing 3-Isobutyl-1-methylxanthine (500 μM), dexamethansone (1 μM), insulin (20 nM), T3 (1 nM) and indomethacin (125 μM) for 2 days. Afterwards, iBAs were refreshed with maintenance medium containing insulin (20 nM), T3 (1 nM). Differentiated iBAs were re-seeded at day 5, and all the experiments were routinely performed at day 9. Lipofectamine RNAiMAX reagent-mediated siRNA (100 nM) delivery was performed to knock down target genes for 72 h in iBAs after one day recovery from replating. To deprive glucose, we either added 2-DG or refreshed cells with DMEM containing different glucose concentrations prepared from the DMEM (Gibco, A1443001) with the same concentration of insulin and T3 as the maintenance medium.

HEK293T (ab255449, Abcam) and HEK293-AAV cells (AAV-100, Cell Biolabs) were cultured in DMEM (high glucose) with 10% FBS and 1% Pen/Strep. HEK293-AAV cells were used for imaging, whereas HEK293T cells were adopted for the rest of the work. We used Lipofectamine 2000 to transiently overexpress EGFP–P4M, EGFP–SACM1L, EGFP–SACM1LK2A, ATGL–FLAG, ATGL–HA, ATGLK0–HA and FBXW8–FLAG in the HEK cells for diverse experimental designs. To image LDs, HEK293-AAV cells were induced by 200 μM OA (O1008 from Sigma) conjugated to bovine serum albumin (BSA, A6003, Sigma) for 24 h before 4% paraformaldehyde (PFA) fixation. Afterwards, cells were stained by BODIPY 493/503 and Hoechst 33342. In other conditions, HEK cells were not treated with OA-containing medium.

HepG2 cells (HB-8065, ATCC) were cultured in RPMI medium with 10% FBS and 1% Pen/Strep on collagen-coated plates. Lipofectamine RNAiMAX reagent-mediated siRNAs (100 nM) were delivered to cells at the suspended state for 48 h. It is not necessary to induce LDs via OA addition in the HepG2 cells before LD imaging. We treated HepG2 cells with 10 μM PF-04620110& PF-06424439 for 48 h or with 5 μg ml^−1^ BFA (B5936, Sigma) for 6 h to deplete LDs and intact Golgi in the HepG2 cells, respectively, before glucose deprivation.

All the cell lines used in this work were regularly tested negative for mycoplasma contamination. All the siRNAs used in these cell lines are listed in Supplementary Table 1.

### RNA extraction and qPCR

Total RNA was extracted via TRIzol reagent (Invitrogen) according to the manufacturer’s instructions. One microgram of total RNA was used to convert into complementary DNA library by using the High-Capacity cDNA Reverse Transcription Kit (Applied Biosystems, 4368813). Quantitative polymerase chain reaction (qPCR) was performed using KAPA SYBR FAST Universal kit (Roche, KK4618) on ViiA7 (Applied Biosystems), and the relative transcript levels were normalized to *36B4* expression via the ΔΔCt method. Primer sequences are listed in Supplementary Table 2.

### Molecular cloning

Human ATGL–FLAG and ATGLK0–FLAG expression constructs were generated as described previously^20^. Based on these vectors, we designed primers to replace FLAG with HA tag and insert them into pEGFP-N1-FLAG vector (Addgene, 60360) to obtain ATGL–HA and ATGLK0–HA expression constructs. Human SACM1L and SACM1LK2A coding sequence (CDS) were cloned from mCherry-FKBP-SAC1 (Addgene, 108127) and inserted into pEGFP-C1-FLAG vector (Addgene, 46956) to have EGFP tag in the N-terminus. Human FBXW8–FLAG CDS was cloned from HEK293T cDNA library and inserted into pLenti-CMV-MCS-BSD vector to obtain pLenti-FBXW8–FLAG construct. Human FBXW8 mutant CDS with substitution of lysines and arginines into alanines in the N terminus was ordered from Vectorbuilder, and the CDS was then inserted into pLenti-CMV-MCS-BSD vector to obtain pLenti-FBXW8 mutant–FLAG construct. Scarlet-Giantin CDS was cloned from plasmid pmScarlet-Giantin-C1 (Addgene, 85048) and inserted into pLenti-CMV-MCS-BSD vector to obtain pLenti-Scarlet-Giantin construct. Sequences to transcribe six different gRNAs targeting individual gene were ordered from Microsynth AG and inserted into AAV-U6-TBG-Cre vectors so that AAV-U6-Pi4kb gRNA-TBG-Cre, AAV-U6-Sacm1l gRNA-TBG-Cre, AAV-U6-Cul7 gRNA-TBG-Cre and AAV-U6-Fbxw8 gRNA-TBG-Cre were constructed. All the plasmids for cell culture work were extracted by NucleoBond Xtra-Midi kit (Macherey-Nagel, 740410.100).

### Virus packaging and stable cell line construction

For lentivirus packaging, the pLenti-Scarlet-Giantin, pLenti-FBXW8–FLAG or pLenti-FBXW8 mutant–FLAG plasmids were transfected into HEK293T cells together with pMD2.G (Addgene, 12259) and psPAX2 (Addgene, 12260) by polyethylenimine in OptiMEM medium. The virus-containing medium was collected and concentrated in PEG-it Virus Precipitation Solution (SBI, LV825A-1) according to the manufacturer’s instructions. The concentrated lentiviruses and polybrene (Sigma, H9268) were added into HEK cells for later selection by 10 μg ml^−1^ blasticidin (ThermoFisher, A1113903).

For AAV8 packaging, the AAV8 serotype helper plasmid (PF0478, PlasmidFactory) and various AAV plasmids were transfected into HEK293-AAV cells via polyethylenimine in OptiMEM medium. The medium was collected for concentration via AAVanced Concentration Reagent (SBI, AAV110A-1) according to the manufacturer’s instructions. The titre of concentrated viruses was determined by qPCR based on titre determination protocol from Addgene.

### Golgi apparatus and LD extraction

Minute Golgi Apparatus Enrichment Kit (GO-037, Invent Biotechnologies) was used to fractionate Golgi and Golgi-related vesicles according to the manufacturer’s instructions. LDs isolation was performed as described previously^59^. Briefly, HepG2 cells were collected in cold phosphate-buffered saline (PBS) and centrifuged at 500*g* for 10 min to pellet cells that were incubated in cold hypotonic lysis medium (20 mM Tris–HCl pH 7.4 and 1 mM ethylenediaminetetraacetic acid (EDTA)) protease inhibitors (Complete, Roche). After being homogenized in a Dounce, the homogenate was centrifuged at 1,000*g* for 10 min to collect the supernatant for further ultracentrifugation via hypotonic lysis medium at different sucrose concentrations. The buoyant fractions were collected and finally suspended in 1% sodium dodecyl sulfate containing RIPA buffer for immunoblotting analyses.

### Immunoblotting, immunoprecipitation, co-IP and immunofluorescence

Tissues and cell cultures were homogenized in RIPA buffer (50 mM Tris–HCl pH 7.4, 150 mM NaCl, 2 mM EDTA, 1.0% Triton X-100 and 0.5% sodium deoxycholate) with protease inhibitors and phosphatase inhibitors (Halt phosphatase inhibitor cocktail, ThermoFisher). The homogenates were centrifuged at 12,000*g* for 10 min at 4 °C to collect WCE. The concentration of WCE was determined by DC Protein Assay (Bio-Rad). After boiling the sample with Lämmeli buffer at 95 °C for 5 min, equal amount of proteins was loaded and separated on 12% sodium dodecyl sulfate polyacrylamide gel electrophoresis. The proteins were transferred to nitrocellulose membrane and blotted by different antibodies. The primay antibody signal was visualized by horseradish peroxidase-conjugated secondary antibodies (1:5,000, Millipore) and the ImageQuant system (GE Healthcare Life Sciences).

To immunoprecipitate FLAG-tagged ATGL or FBXW8, HEK293T cells were homogenized in RIPA buffer (50 mM Tris–HCl pH 7.4, 150 mM NaCl, 2 mM EDTA and 1.0% Triton X-100) with protease inhibitors and phosphatase inhibitors. The homogenates were centrifuged at 12,000*g* for 10 min at 4 °C to collect WCE. After three washes with cold RIPA buffer, 20 μl Anti-FLAG beads (A2220, Sigma) was added to WCE for overnight incubation with rotation at 4 °C. The beads were then washed six times by cold RIPA buffer. The proteins were eluted by boiling the beads at 95 °C for 5 min with 2× Lämmeli buffer. The eluted protein was then analysed by immunoblotting.

To immunostain ATGL, GM130, CUL7, FBXW8 and FBXW8–FLAG in HEK293-AAV cells or a stable cell line expressing Scarlet-Giantin to visualize Golgi after various treatments, HEK293-AAV cells were fixed by 4% PFA for 20 min and permeabilized by 0.25% PBST (Triton X-100 in PBS) or 20 μM digitonin in PBS (ATGL immunostaining) for 20 min. After three washes with cold PBS, cells were blocked by 1% BSA for 1 h and incubated with primary antibodies overnight at 4 °C. After three washes with cold PBS, cells were incubated with Alexa Fluor 488 conjugated secondary antibody (1:200, ThermoFisher) for 1 h at room temperature. Finally, cells were stained by Hoechst 33342 to label nuclei after three washes with PBS to remove residual secondary antibody. We determined the ATGL intensity/Golgi area ratio or FBXW8 intensity/Golgi area ratio to quantify the Golgi ATGL or FBXW8 levels, respectively. Likewise, we used the same procedures to immunostain GM130 in HepG2 cells except for using Alexa Fluor 568 conjugated secondary antibody (1:200, ThermoFisher).

To immunostain ATGL in the iBAT, the tissues were fixed by 4% PFA overnight 4 °C after dissection. Then 10% sucrose and 30% sucrose solution were used to dehydrate samples before cryosection at 15 μm. The slides were washed once in PBS for 5 min and 3 washes in 0.1% PBST before blocking with 10% normal donkey serum diluted in PBST for 1 h. Afterwards, ATGL antibody (1:100, Cell Signaling) was diluted in blocking solution for overnight incubation at 4 °C. The residual antibody was washed with PBST three times before application of Alexa Fluor 568 conjugated secondary antibody (1:200 in 5% normal donkey serum, ThermoFisher) for 1 h. At last, the slides were treated with mounting solution after three washes with PBST.

The pictures were all obtained by Olympus FluoView 3000 confocal microscope and processed by ImageJ.

### PtdIns4P determination

HEK293-AAV cells were transfected with Scarlet-Giantin and EGFP–P4M and glucose-deprived. The cells were live-imaged, and the Golgi PtdIns4P levels were determined as Golgi GFP intensity/cytosolic GFP intensity ratio in the individual cells. To immunostain cellular PtdIns4P at different conditions, Scarlet-Giantin expressing HEK293-AAV cells were fixed by 4% PFA for 15 min and washed three times with PBS containing 50 mM NH_4_Cl, followed by permeabilization for 5 min with 20 μM digitonin in PBS. After being washed three times with PBS, cells were blocked with 5% normal goat serum (NGS) in PBS with 50 mM NH_4_Cl for 1 h at room temperature. Primary anti-PtdIns4P antibody (1:100 in 5% NGS; Echelon Biosciences) was applied to cells overnight at 4 °C. Alexa Fluor 488 conjugated secondary antibody (1:200 in 5% NGS, ThermoFisher) was replaced with three washes before capturing images under microscope. We determined the PtdIns4P intensity/Golgi area ratio to quantify the Golgi PtdIns4P. PtdIns4P Mass ELISA Assay Kit (K-4000E, Echelon Biosciences) was used to determine the total mass of PtdIns4P from HEK293T cells according to the manufacturer’s instructions.

### Membrane Lipid Strip assay, PIP Strip assay and pull-down by PtdIns4P agarose beads

FLAG Purification Kit (CELLMM2, Sigma) was used to purify wild-type and mutant FBXW8 from established HEK293T stable cell lines according to the manufacturer’s instructions. The eluted proteins were filtered and concentrated using a 10-kDa cut-off concentrator (Pierce, 88513).

Membrane Lipid Strip (P-6002, Echelon Biosciences) and PIP Strip (P-6001, Echelon Biosciences) were used to perform binding assays between recombinant proteins and lipids as described previously^23^. PtdIns4P-conjugated beads (P-B004, Echelon Biosciences), phosphatidylcholine-conjugated beads (P-B0PC, Echelon Biosciences) and control beads (P-B000, Echelon Biosciences) were used to pull down purified recombinant proteins according to the manufacturer’s instructions. Briefly, 100 μl slurry was pelleted by centrifugation at low speed and then resuspended in 300 μl wash-binding buffer (10 mM HEPES, pH 7.4, 150 mM NaCl and 0.25% Igepal). Ten micrograms recombinant wild-type and mutant FBXW8 were added for 3 h incubation. The beads were pelleted and washed with wash-binding buffer six times. At last, the proteins were eluted by boiling the beads at 95 °C for 5 min with 2× Lämmeli buffer and analysed by immunoblotting.

### Lipolysis measurement

Day 9 iBAs were washed with pre-warmed PBS and then starved by phenol-red-free DMEM (low glucose) with 1% FA-free BSA for 2 h. After medium collection, NEFA release was measured with an NEFA assay kit (Wako NEFA kit) and normalized to total protein mass. Similarly, the dialysates from liver graft during ex vivo perfusion were measured to determine NEFA levels. All the photometric and fluorometric measurements were performed via Synergy Gen5 Platereader.

### Measurement of insulin, glucose, FFA, TG and ALT and AST activity in the plasma

Plasma insulin levels were determined via Ultra-sensitive Mouse Insulin ELISA Kit (Crystal Chem, 90080). Plasma glucose levels were determined via glucose assay kit (Sigma, MAK263). Plasma FFA levels were determined via NEFA assay kit (Wako NEFA kit). Plasma TG levels were determined via TG assay kit (Roche Trig/GB reagent). Alanine transaminase (ALT) (MAK052, Sigma) and aspartate aminotransferase (AST) (MAK055, Sigma) assay kits were used to determine ALT and AST activity. All the assays were performed according to the manufacturers’ instructions.

### Determination of hepatic glucose, glucose-6-phosphate and glyceraldehyde 3-phosphate levels

Liver pieces were homogenized in two volumes of ice-cold PBS. The homogenates were centrifuged at 13,000*g* for 10 min to collect supernatant, which was deproteinized with a 10 kDa molecular weight cut-off spin filter (Pierce, 88513). One microlitre sample was used to measure glucose levels via Glucose Assay Kit (Sigma, MAK263). Ten microlitres sample was used to measure glucose-6-phosphate levels via Glucose-6-Phosphate Assay Kit (Sigma, MAK014) or to measure glyceraldehyde 3-phosphate levels via Glyceraldehyde 3-Phosphate Assay Kit (Abcam, ab273344). All the concentrations were normalized to the protein concentration.

### Liver FA oxidation and TG determination

We measured liver FAO as described previously^20,60^. To measure TG levels, weight of liver pieces (50–100 mg) was recorded before homogenization by 1 ml isopropanol per 50 mg tissue. The homogenates were incubated with rotating before centrifugation at 2,000*g* for 10 min to collect supernatant. The TG levels were determined via Roche Trig/GB reagent.

### Histologic staining

Livers were fixed in 4% PFA for 24 h directly after tissue collection. To perform haematoxylin and eosin (H&E) staining, liver pieces were transferred to 65% ethanol and embedded in paraffin in tissue processing machine and embedding machine, followed by section at 10 μm and staining using the standard protocol. To perform Oil Red O (ORO) staining, the fixed livers were dehydrated before cryosection at 20 μm and staining via ORO solution (O1516, Sigma).

For MASH cohort, livers were fixed with 4% PFA for 24 h at 4 °C. The samples were sent to the University of Zurich for histologic analyses. Briefly, liver tissues were dehydrated and embedded in paraffin before section at 3 μm, followed by staining with H&E and Gomori Green Trichrome or anti-cleaved Caspase-3 after antigen retrieval. The images were captured by digital slide scanners, and the grade of steatosis, ballooning, inflammation and fibrosis were quantified via the MASH Clinical Research Network Scoring System^61^. For ORO staining, the fixed samples were cryosectioned, and the slides were stained via ORO solution and eosin.

### Statistics and reproducibility

For in vivo studies, littermates were randomly assigned to treatment groups for all the experiments, but no randomization was performed for in vitro experiments. Sample size was determined on the basis of previous experiments, and the animal numbers used in the experiments are all indicated in the corresponding figure legends. No data were excluded from the analyses, and the investigators were not blinded to allocation during experiments and outcome assessment. For cell culture experiments, biological replicates were no less than three (shown as *n* in the figure legend), and all the cell culture experiments were performed with two to three technical replicates for RNA and protein analysis. Results are represented as mean ± s.e.m. when it is not indicated specifically. Two-tailed unpaired or paired Student’s *t*-test was applied on comparison of two groups when the data are in normal distribution, otherwise Mann–Whitney test or Wilcoxon test was adopted. Analysis of variance (ANOVA) was applied on comparisons of multiple groups when the data are in normal distribution, otherwise Kruskal–Wallis test was adopted. All statistical analyses were performed using GraphPad Prism 9. Statistical differences are indicated as exact *P* value or non-significant (n.s.).

### Reporting summary

Further information on research design is available in the Nature Portfolio Reporting Summary linked to this article.

## Online content

Any methods, additional references, Nature Portfolio reporting summaries, source data, extended data, supplementary information, acknowledgements, peer review information; details of author contributions and competing interests; and statements of data and code availability are available at 10.1038/s41556-024-01386-y.

### Supplementary information

Reporting Summary

Supplementary Table 1 It contains the sequences of siRNAs and primers used in this work.

### Source data

Source Data Fig. 1 Statistical source data.

Source Data Fig. 2 Unprocessed western blot.

Source Data Fig. 2 Statistical source data.

Source Data Fig. 3 Unprocessed western blot.

Source Data Fig. 3 Statistical source data.

Source Data Fig. 4 Unprocessed western blot.

Source Data Fig. 4 Statistical source data.

Source Data Fig. 5 Unprocessed western blot.

Source Data Fig. 5 Statistical source data.

Source Data Fig. 6 Unprocessed western blot.

Source Data Fig. 6 Statistical source data.

Source Data Fig. 7 Unprocessed western blot.

Source Data Fig. 7 Statistical source data.

Source Data Extended Data Fig./Table 1 Unprocessed western blot.

Source Data Extended Data Fig./Table 1 Statistical source data.

Source Data Extended Data Fig./Table 2 Unprocessed western blot.

Source Data Extended Data Fig./Table 2 Statistical source data.

Source Data Extended Data Fig./Table 3 Unprocessed western blot.

Source Data Extended Data Fig./Table 3 Statistical source data.

Source Data Extended Data Fig./Table 4 Unprocessed western blot.

Source Data Extended Data Fig./Table 4 Statistical source data.

Source Data Extended Data Fig./Table 5 Unprocessed western blot.

Source Data Extended Data Fig./Table 5 Statistical source data.

Source Data Extended Data Fig./Table 6 Unprocessed western blot.

Source Data Extended Data Fig./Table 6 Statistical source data.

Source Data Extended Data Fig./Table 7 Unprocessed western blot.

Source Data Extended Data Fig./Table 7 Statistical source data.

Source Data Extended Data Fig./Table 8 Unprocessed western blot.

Source Data Extended Data Fig./Table 8 Statistical source data.

Source Data Extended Data Fig./Table 9 Unprocessed western blot.

Source Data Extended Data Fig./Table 9 Statistical source data.

## Data Availability

Source data are provided with this paper. All other data supporting the findings of this study are available from the corresponding author on reasonable request.
