# Supplementary material for: Glucose controls lipolysis through Golgi PtdIns4P-mediated regulation of ATGL
Source: Nat Cell Biol. 2024 Apr 1;26(4):552–66. doi: 10.1038/s41556-024-01386-y (PMC11021197; doi:10.1038/s41556-024-01386-y)
Supplement: Supplementary file 1 — Reporting Summary [file 41556_2024_1386_MOESM1_ESM.pdf]

nature research

Corresponding author(s): Christian Wolfrum

Last updated by author(s): Feb 6, 2024

Reporting Summary

Nature Research wishes to improve the reproducibility of the work that we publish. This form provides structure for consistency and transparency in reporting. For further information on Nature Research policies, see our Editorial Policies and the Editorial Policy Checklist.

Statistics

For all statistical analyses, confirm that the following items are present in the figure legend, table legend, main text, or Methods section.

n/a	Confirmed	
☐	☒	The exact sample size (*n*) for each experimental group/condition, given as a discrete number and unit of measurement
☐	☒	A statement on whether measurements were taken from distinct samples or whether the same sample was measured repeatedly
☐	☒	The statistical test(s) used AND whether they are one- or two-sided<br>*Only common tests should be described solely by name; describe more complex techniques in the Methods section.*
☐	☒	A description of all covariates tested
☐	☒	A description of any assumptions or corrections, such as tests of normality and adjustment for multiple comparisons
☐	☒	A full description of the statistical parameters including central tendency (e.g. means) or other basic estimates (e.g. regression coefficient) AND variation (e.g. standard deviation) or associated estimates of uncertainty (e.g. confidence intervals)
☐	☒	For null hypothesis testing, the test statistic (e.g. *F*, *t*, *r*) with confidence intervals, effect sizes, degrees of freedom and *P* value noted<br>*Give P values as exact values whenever suitable.*
☒	☐	For Bayesian analysis, information on the choice of priors and Markov chain Monte Carlo settings
☒	☐	For hierarchical and complex designs, identification of the appropriate level for tests and full reporting of outcomes
☒	☐	Estimates of effect sizes (e.g. Cohen's *d*, Pearson's *r*), indicating how they were calculated

Our web collection on statistics for biologists contains articles on many of the points above.

Software and code

Policy information about availability of computer code

Data collection	No specific software was used
Data analysis	ImageJ (version 1.53e) was used to analyze western blots and images. Graphpad Prism 9 was used to perform statistical analyses.

For manuscripts utilizing custom algorithms or software that are central to the research but not yet described in published literature, software must be made available to editors and reviewers. We strongly encourage code deposition in a community repository (e.g. GitHub). See the Nature Research guidelines for submitting code & software for further information.

Data

Policy information about availability of data

All manuscripts must include a data availability statement. This statement should provide the following information, where applicable:

- Accession codes, unique identifiers, or web links for publicly available datasets
- A list of figures that have associated raw data
- A description of any restrictions on data availability

All raw data are available

April 2020

Field-specific reporting

Please select the one below that is the best fit for your research. If you are not sure, read the appropriate sections before making your selection.

☒ Life sciences ☐ Behavioural & social sciences ☐ Ecological, evolutionary & environmental sciences

For a reference copy of the document with all sections, see nature.com/documents/nr-reporting-summary-flat.pdf

Life sciences study design

All studies must disclose on these points even when the disclosure is negative.

Sample size	Sample sizes were determined on the basis of previous experiments in our lab and common standard in the metabolism field using similar methods (ref 18, 20).
Data exclusions	No samples were excluded from any analyses.
Replication	Experimental findings were verified by biological replicates and technical replicates, each experiment was performed multiple times as indicated in the figure legends.
Randomization	All experiments were randomized
Blinding	The investigators were not blinded to the mice as they themselves were treating and sacrificing the mice. However, the investigators were blinded for sample processing.

Reporting for specific materials, systems and methods

We require information from authors about some types of materials, experimental systems and methods used in many studies. Here, indicate whether each material, system or method listed is relevant to your study. If you are not sure if a list item applies to your research, read the appropriate section before selecting a response.

Materials & experimental systems

n/a	Involved in the study	
☐	☒	Antibodies
☐	☒	Eukaryotic cell lines
☒	☐	Palaeontology and archaeology
☐	☒	Animals and other organisms
☐	☒	Human research participants
☒	☐	Clinical data
☒	☐	Dual use research of concern

Methods

n/a	Involved in the study	
☒	☐	ChIP-seq
☒	☐	Flow cytometry
☒	☐	MRI-based neuroimaging

Antibodies

Antibodies used	ATGL (1:1000, Cell Signaling:2138S), CGI-58 (1:1000, Proteintech:12201-1-AP), phosphor-HSL(Ser660) (1:1000, Cell Signaling:45804S), PLIN1 (1:1000, Cell Signaling:9349S), HSL (1:1000, Cell Signaling:4107S), γ-tubulin (1:10000, Sigma:T6557), FLAG (1:10000, Sigma:F3165; 1:1000, Cell Signaling:2368S and 8146S), MGL (1:1000, Abcam:ab77398), AKT (1:1000, Cell signaling:9272), Phospho-AKT(1:1000, Cell Signaling:4060), CUL7 (1:1000, Santa Cruz:sc-53810), HSP90 (1:1000, Cell Signaling:4877S), K48 linkage specific ubiquitin antibody (1:1000, Cell Signaling:33959), HA (1:1000, Cell signaling:3724S), FBXW8 (1:1000, Santa Cruz:sc-514385; 1:1000, Sigma:HPA038851), PI4KB antibody (1;1000, Sigma:HPA006280), SACM1L(1:1000, Sigma:HPA069869), PtdIns4P (1:100, Echelon Biosciences:Z-P004), TGN46 (1:1000, Abcam:ab2809), PLIN2 (1:1000, Abcam:ab108323), GP73 (1:1000, Sigma:HPA011929), MAOA (1:1000, Abcam:ab126751), PMP70 (1:10000, Sigma:SAB4200181), Calnexin (1:1000, Cell signaling:2433S), ARF1 (1:1000, Invitrogen:PA1-127), RAB7 (1:1000, Cell Signaling:9367T), Na+/K+ ATPase (1:1000, Abcam:ab7671), GAPDH (1:10000, Cell Signaling:2118S), cleaved Caspase-3 (1:1000, Cell Signaling:9661), HRP-conjugated anti-rabbit IgG (1:10000, Cell signaling: 7074), HRP-conjugated anti-mouse IgG (1:10000, Cell signaling:7076),Anti-Rabbit IgG (H+L) Cross-Adsorbed Secondary Antibody, Alexa Fluor 488 (1:200,Thermo:A21206), Anti-Rabbit IgG (H+L) Cross-Adsorbed Secondary Antibody, Alexa Fluor 568 (1:200, Thermo:A11042),Anti-Mouse IgG (H+L) Highly Cross-Adsorbed Secondary Antibody, Alexa Fluor 488 (1:200, Thermo:A11029)
Validation	We validated ATGL,PI4KB, SACM1L, CUL7, FBXW8 and GP73 antibodies in both mouse and human cell lines for WB application via siRNA knockdown.<br>The following antibodies were validated by previous publication or commercial companies:<br>Anti-CGI-58 for WB of mouse sample (DOI: 10.1074/jbc.RA120.014682), anti-pHSL for WB of mouse sample (DOI: 10.1038/s42255-021-00489-2), anti-PLIN1 for WB of mouse sample (10.1172/jci.insight.139160), anti-HSL for WB of mouse sample (https://www.cellsignal.com/products/primary-antibodies/hsl-antibody/4107), anti-γ-tubulin for WB of mouse and human sample (https://www.sigmaaldrich.com/CH/de/product/sigma/t6557), anti-FLAG for WB (DOI: 10.1038/s42255-021-00489-2), anti-FLAG for immunostaining (https://www.cellsignal.com/products/primary-antibodies/dykddddk-tag-9a3-mouse-mab-binds-to-same-epitope-

as-sigma-aldrich-anti-flag-m2-antibody/8146), anti-MGL for WB of mouse sample (DOI: 10.3892/etm.2021.11004), anti-AKT for WB of mouse sample (https://www.nature.com/articles/s41467-023-39715-8), anti-phospho-AKT for WB of mouse sample (https://www.nature.com/articles/s41467-023-39715-8), anti-HSP90 for WB of human and mouse sample (https://www.nature.com/articles/s41467-023-39715-8), anti-K48 ubi for WB of human sample (DOI: 10.1038/s42255-021-00489-2), anti-HA for WB (10.1038/s41467-021-24097-6), anti-PtdIns4P for IF of human cells (https://doi.org/10.1083/jcb.201905162), anti-PLIN2 for WB of human samples (https://www.abcam.com/products/primary-antibodies/rabbit-monoclonal-epr3713-to-perilipin-2-ab108323.html),anti-MAOA for WB of human samples (https://www.abcam.com/products/primary-antibodies/monoamine-oxidase-amao-a-antibody-epr7101-ab126751.html), anti-PMP70 for WB of human sample (DOI: 10.1038/s42255-021-00489-2), anti-Calnexin for WB of human sample (https://www.cellsignal.com/products/primary-antibodies/calnexin-antibody/2433), anti-ARF1 for WB of human sample (https://www.thermofisher.com/antibody/product/ARF1-Antibody-Polyclonal/PA1-127), anti-RAB7 for WB of human sample (https://www.cellsignal.com/products/primary-antibodies/rab7-d95f2-xp-rabbit-mab/9367), anti-Na+/K+ ATPase for WB of human sample (https://www.abcam.com/products/primary-antibodies/alpha-1-sodium-potassium-atpase-antibody-4646-ab7671.html), anti-GAPDH for WB of human sample (https://www.cellsignal.com/products/primary-antibodies/gapdh-14c10-rabbit-mab/2118), anti-cleaved Caspase-3 for WB of mouse sample (https://www.cellsignal.com/products/primary-antibodies/cleaved-caspase-3-asp175-antibody/9661)

Eukaryotic cell lines

Policy information about cell lines

Cell line source(s)

293-AAV and 293-LTV cell lines were purchased from Cell Biolabs Inc. (cat. AAV-100; LTV-100); HEK293T cell line was purchased from abcam. iBAs cells were previously established by Ronald Kahn lab as indicated in manuscript. HepG2 cells were purchased from ATCC.

Authentication

293-AAV and 293-LTV cells were not authenticated, however the AAV and LV produced from these cells were titred by PCR. iBA cells were previously validate by gene expression and functional assay (OCR). HepG2 cells and HEK293Tcells were validated via PCR.

Mycoplasma contamination

The cells were regularly tested, all cell lines tested were negative for mycoplasma contamination.

Commonly misidentified lines
(See ICLAC register)

No commonly misidentified cell lines were used in the study.

Animals and other organisms

Policy information about studies involving animals; ARRIVE guidelines recommended for reporting animal research

Laboratory animals

C57Bl/6 male mice were used. Both male and female mice of B6J.129(B6N)-Gt(ROSA)26Sortm1(CAG-cas9*,-EGFP)Fezh/J strain and male mice of Atgl floxed strain were used. All the experiments were started when mice were 8 to 10 weeks of age. Mice were kept on an inverted 12-h:12-h dark:light cycle and fed ad libitum with a chow diet or high fat diet, at 40% humidity and 22 degree.

Wild animals

No wild animals were used in the study.

Field-collected samples

No field-collected samples were used in the study.

Ethics oversight

All animal experiments were approved by the Veterinary office of the Canton of Zurich and Institutional Animal Care and Use Committee of Sun Yat-sen University

Note that full information on the approval of the study protocol must also be provided in the manuscript.

Human research participants

Policy information about studies involving human research participants

Population characteristics

24 middled-aged lean-to-obese sedentary men (age 35.5 ± 1.5 years, BMI 28.9 ± 1.1 kg.m-2, adiposity 25.7 ± 1.6%) with different degree of insulin sensitivity.

Recruitment

The participants included in the study were recruited as part of a larger clinical study previously described in Balaz et al., 2014 (https://doi.org/10.1002/oby.20764). Patients with chronic disease or regular use of pharmacotherapy were not eligible to participate. All patients underwent complex metabolic phenotyping, including assessment of insulin sensitivity by Euglycemic hyperinsulinemic clamp (EHC) and abdominal subcutaneous adipose tissue biopsy both at fasted state, as well as during the steady state of EHC. 24 individuals with sufficient amount of adipose tissue samples were randomly selected for this study without any selection bias, and based on their insulin sensitivity index (M value) assigned to insulin sensitive (n=16, M value 0.14 ± 0.02 mg/kg BW/min/insulin µU/ml) and insulin resistant (n=8, M value 0.04 ± 0.01 mg/kg BW/min/insulin µU/ml) subgroups. All study participants provided witnessed written informed consent before entering the study.

Ethics oversight

The EHC study was approved by the by the Local Ethics Committee (Bratislava, Slovakia) and conforms to the ethical guidelines of the 2000 Helsinki declaration.
The human liver grafts were perfused after being discarded from all Swiss transplant centers. Use of discarded grafts was approved by local authorities (Kantonale Ethik Kommission Zürich KEK Nr. 2017-000412).

Note that full information on the approval of the study protocol must also be provided in the manuscript.
