# Supplementary figures and images for: Glucose controls lipolysis through Golgi PtdIns4P-mediated regulation of ATGL
Source: Nat Cell Biol. 2024 Apr 1;26(4):552–66. doi: 10.1038/s41556-024-01386-y (PMC11021197; doi:10.1038/s41556-024-01386-y)

Fig.2

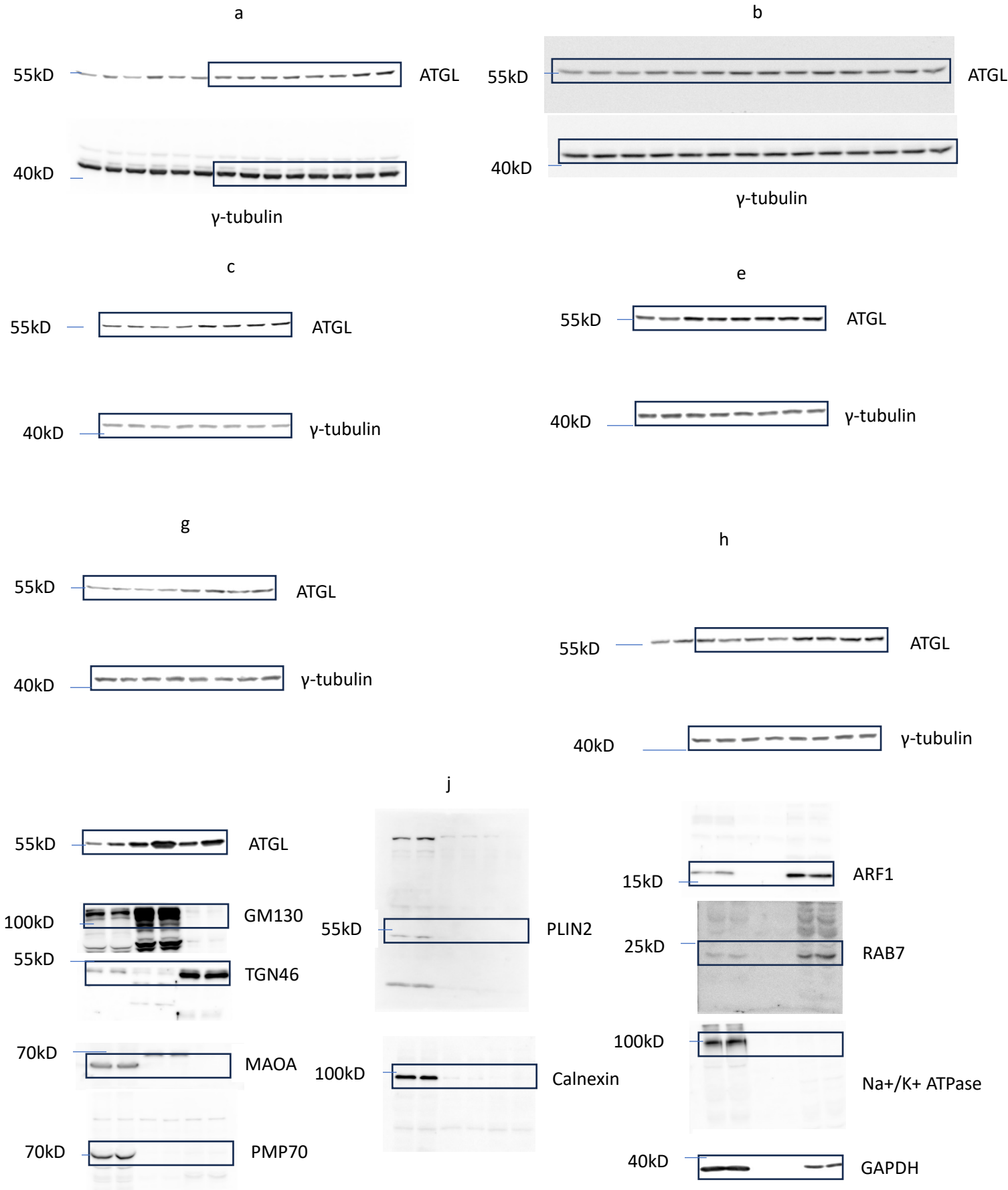

Supplement: Supplementary file 4 — Unprocessed western blot. [file 41556_2024_1386_MOESM4_ESM.pdf]

Fig.3

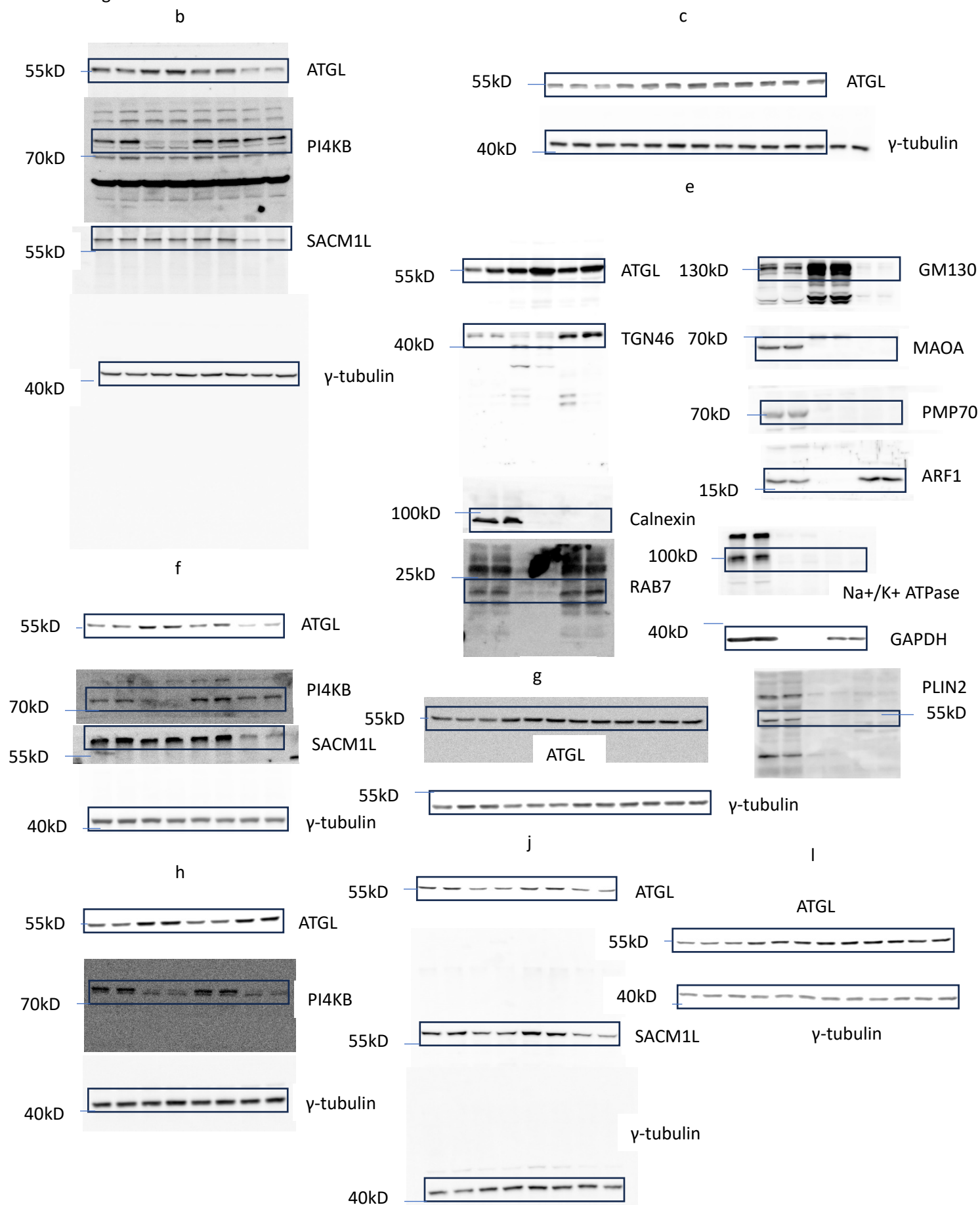

Supplement: Supplementary file 6 — Unprocessed western blot. [file 41556_2024_1386_MOESM6_ESM.pdf]

Fig. 4

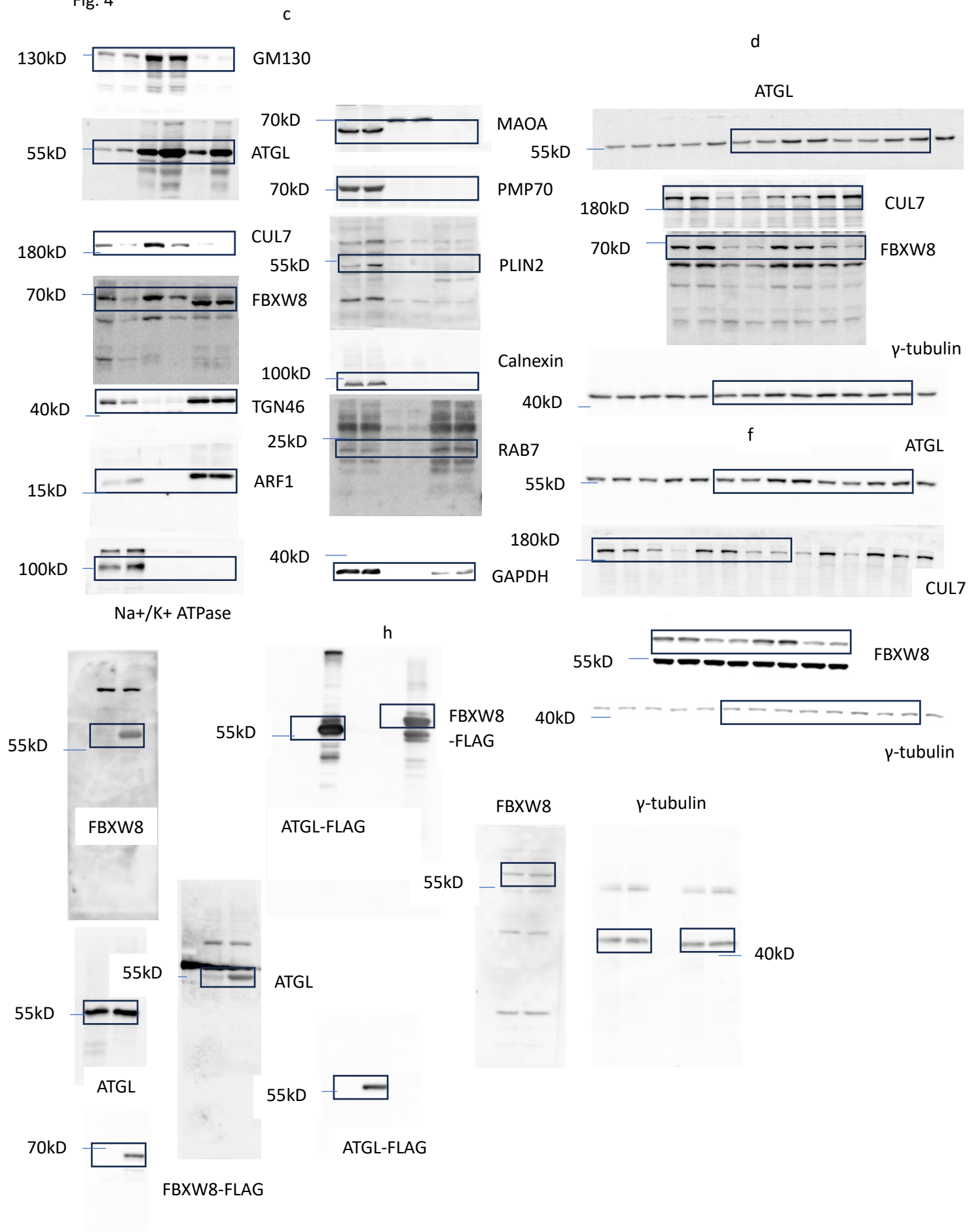

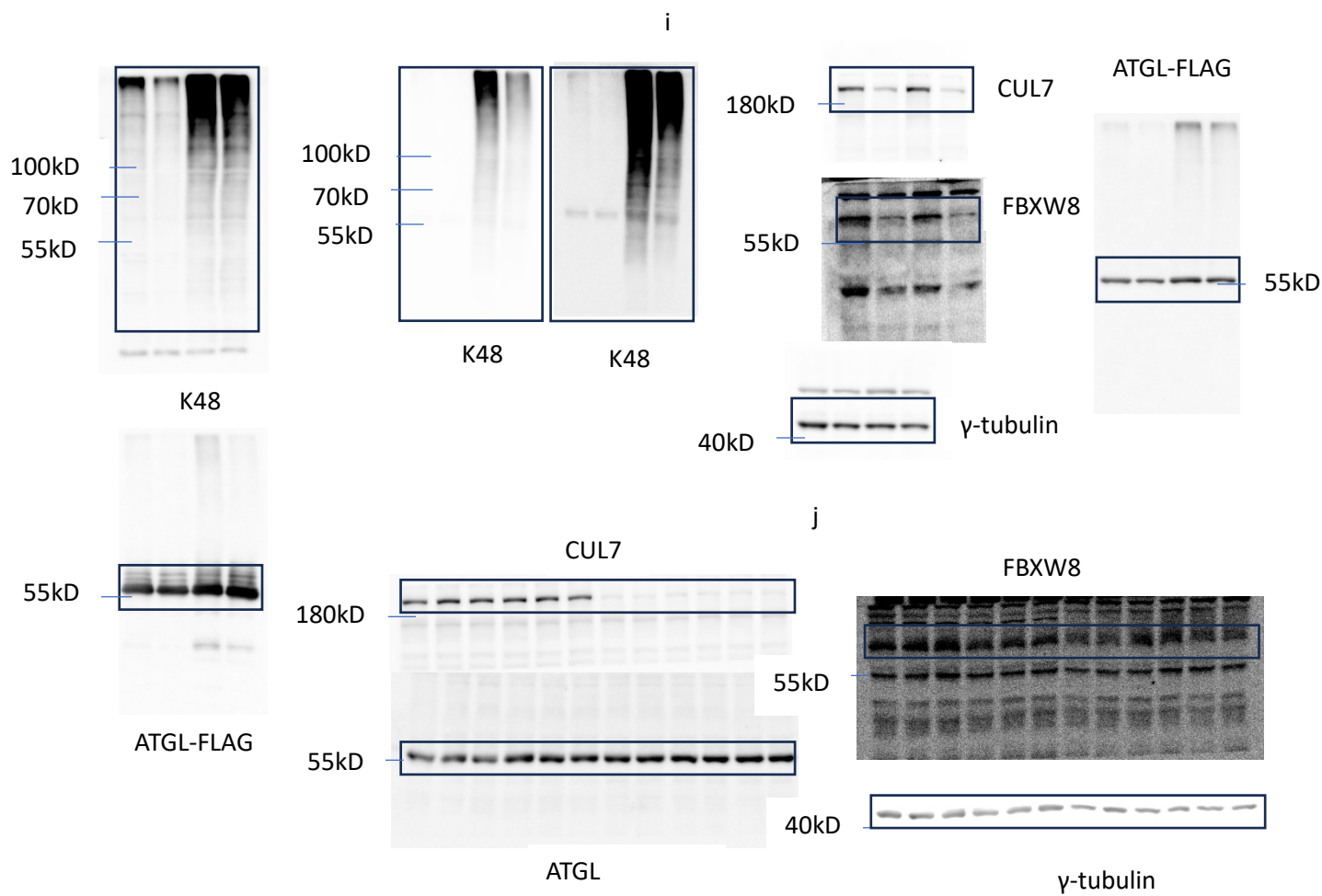

Supplement: Supplementary file 8 — Unprocessed western blot. [file 41556_2024_1386_MOESM8_ESM.pdf]

Fig 5

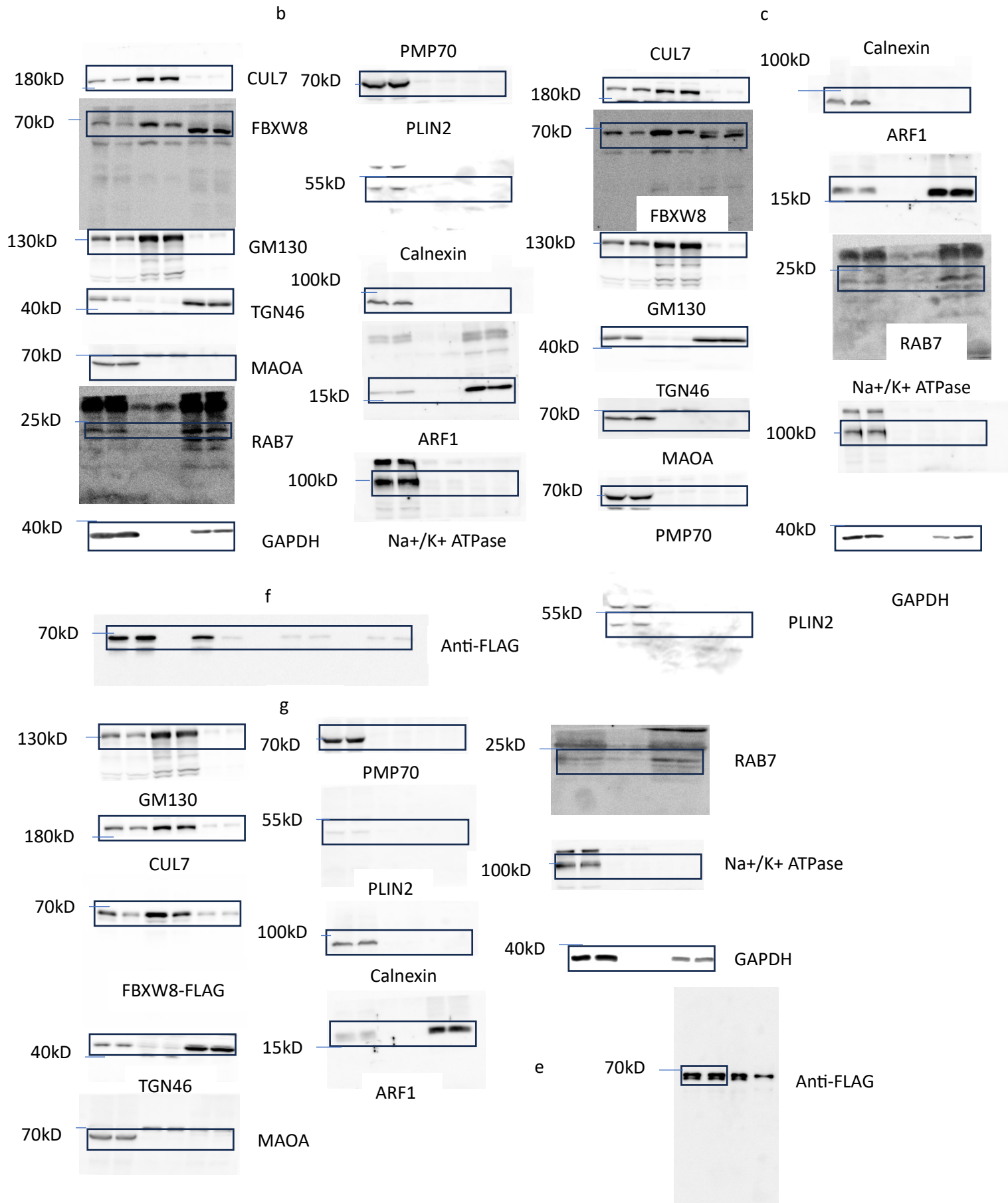

h

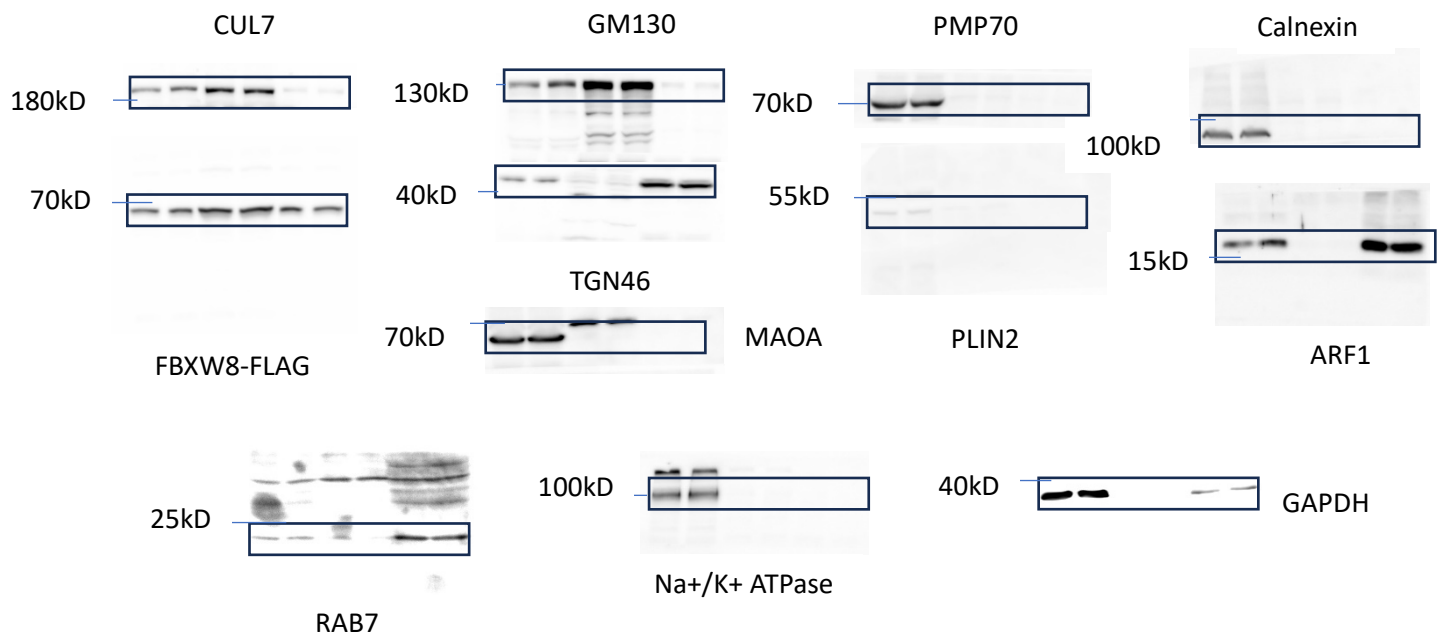

Supplement: Supplementary file 10 — Unprocessed western blot. [file 41556_2024_1386_MOESM10_ESM.pdf]

Fig 6

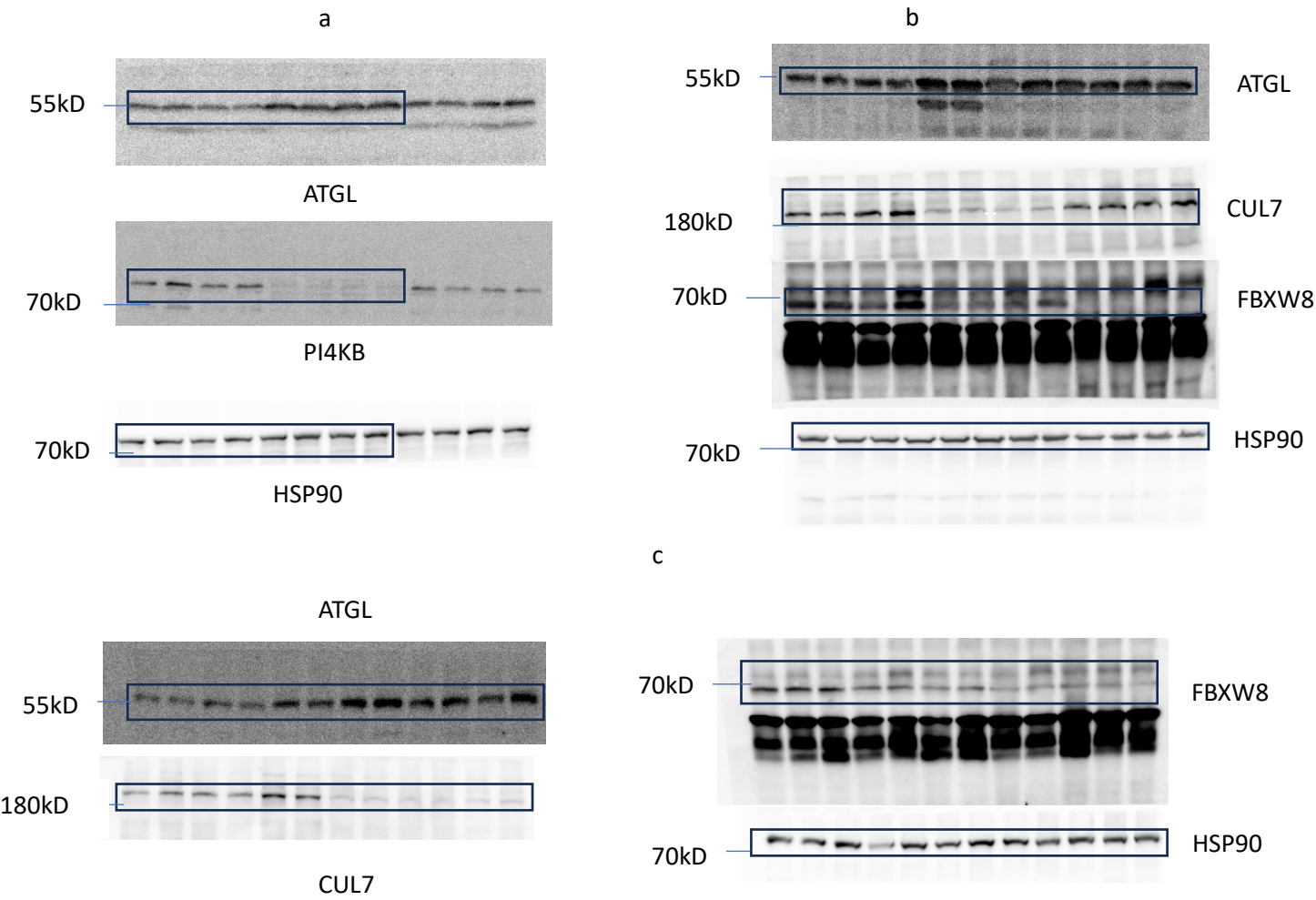

Supplement: Supplementary file 12 — Unprocessed western blot. [file 41556_2024_1386_MOESM12_ESM.pdf]

Fig.7

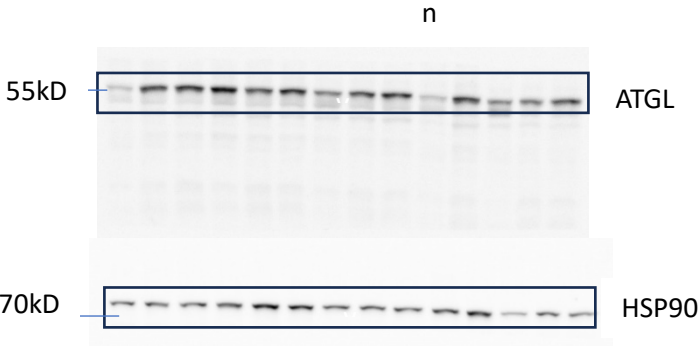

Supplement: Supplementary file 14 — Unprocessed western blot. [file 41556_2024_1386_MOESM14_ESM.pdf]

Extended Data Fig.2

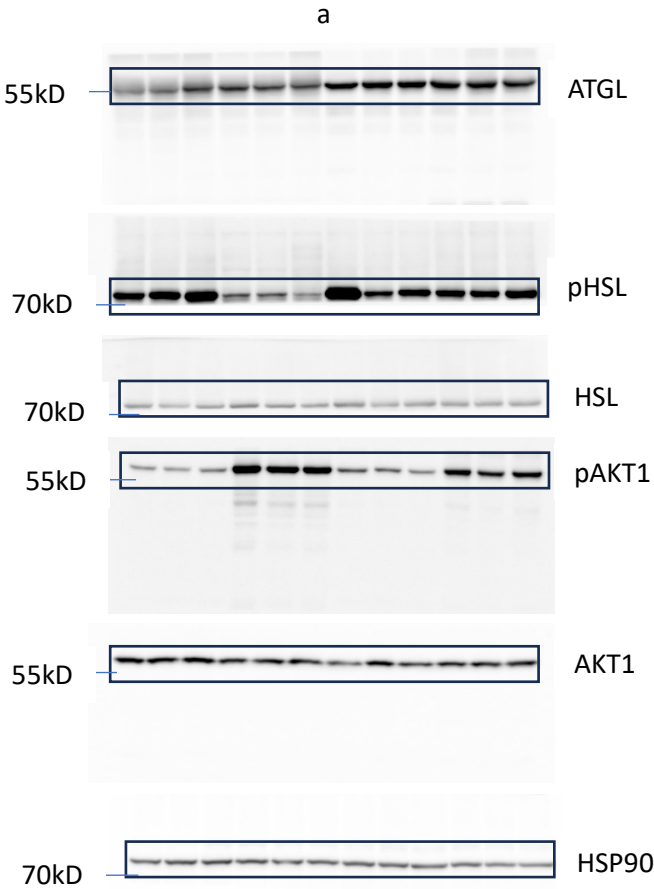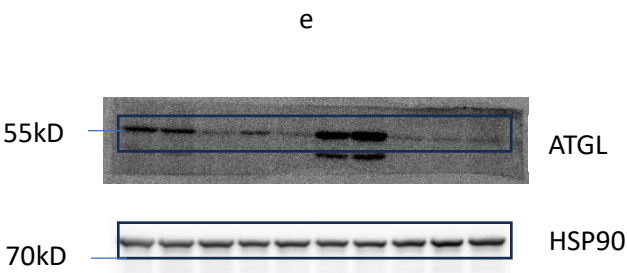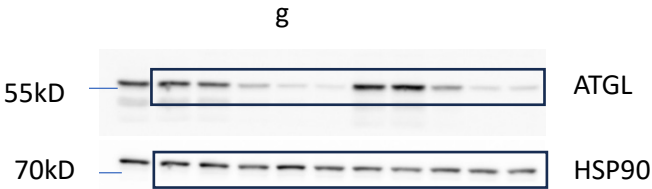

Supplement: Supplementary file 18 — Unprocessed western blot. [file 41556_2024_1386_MOESM18_ESM.pdf]

Extended Data Fig.3

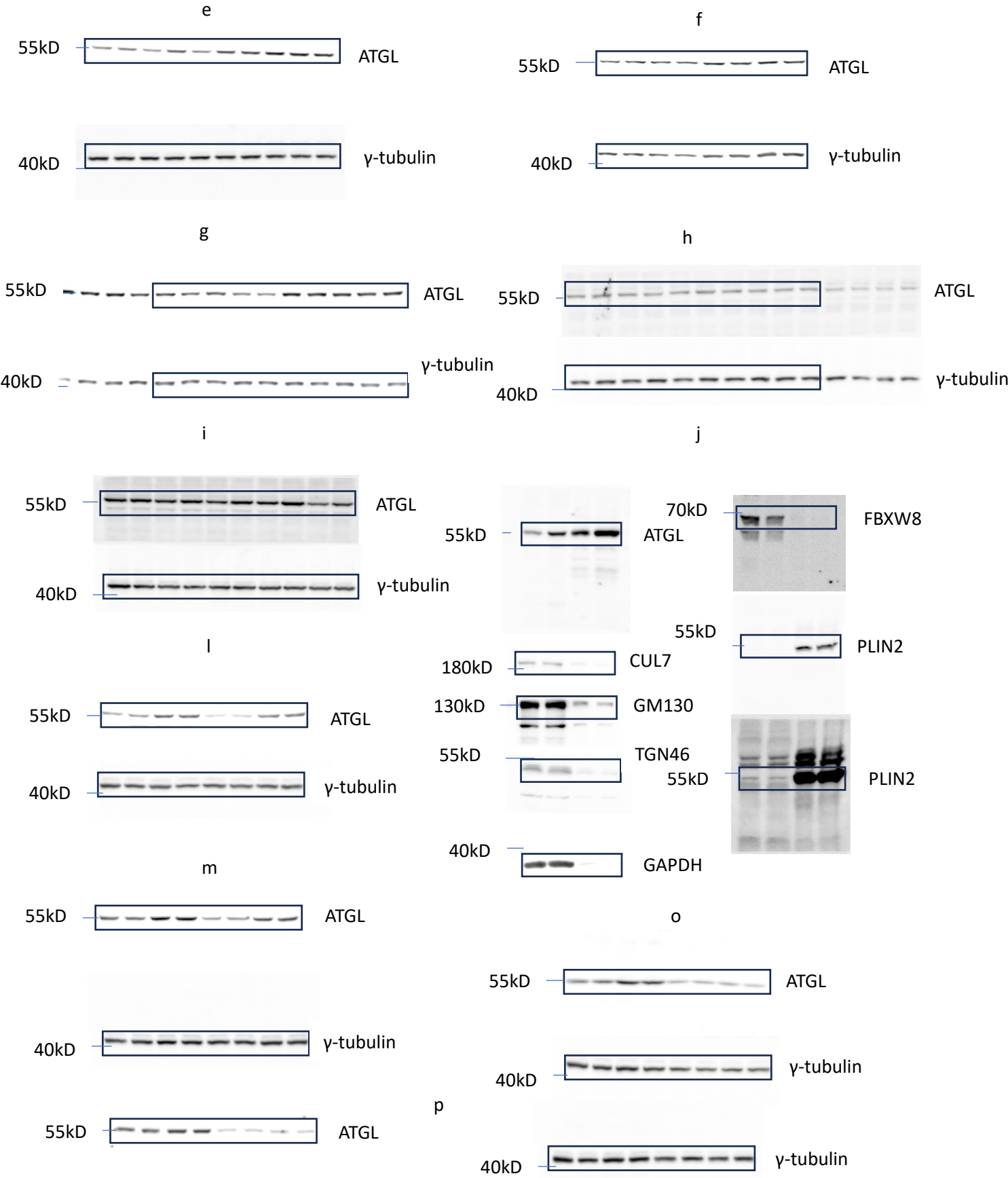

Supplement: Supplementary file 20 — Unprocessed western blot. [file 41556_2024_1386_MOESM20_ESM.pdf]

Extended Data Fig. 4

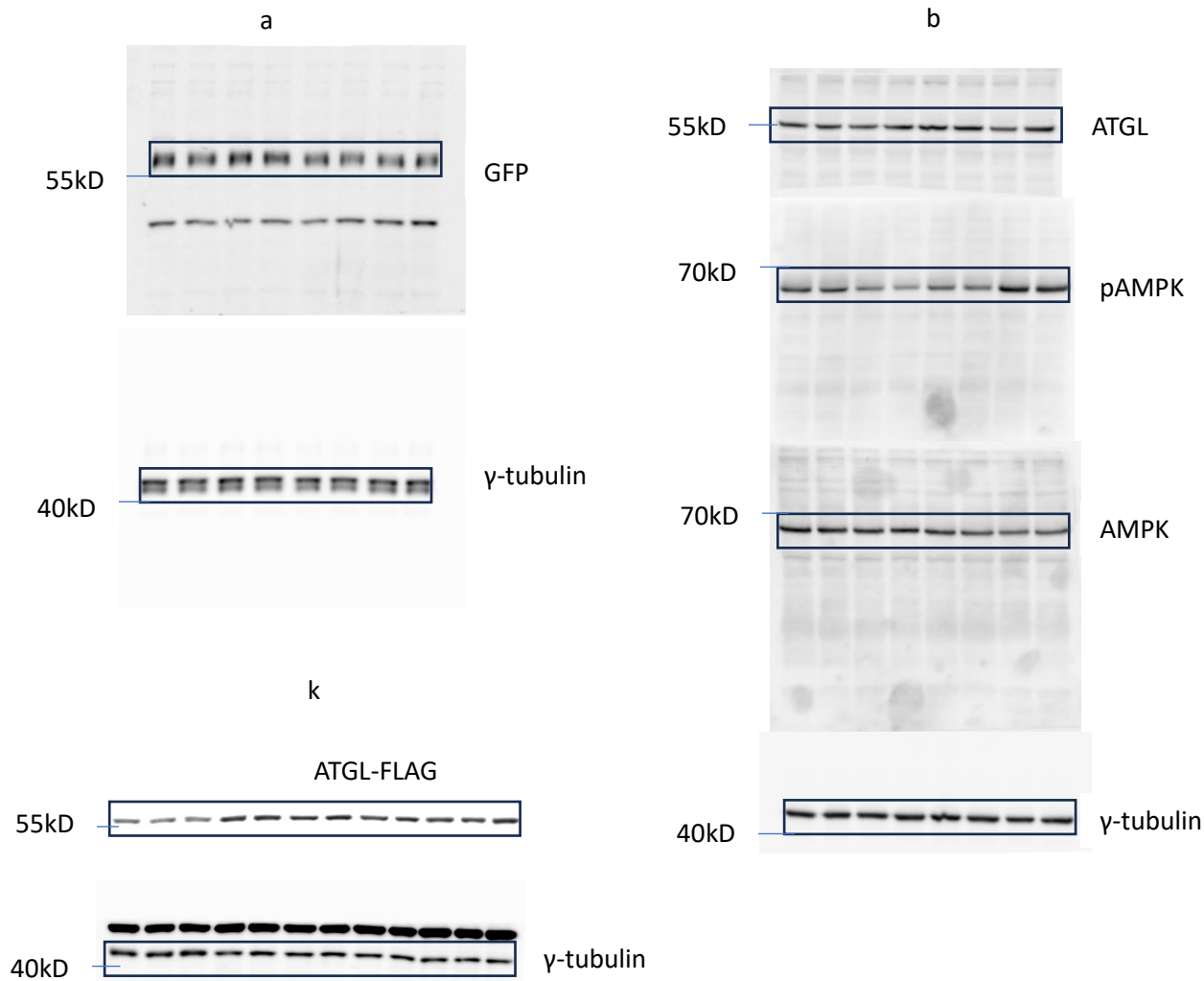

Supplement: Supplementary file 22 — Unprocessed western blot. [file 41556_2024_1386_MOESM22_ESM.pdf]

Extended Data Fig. 5

a

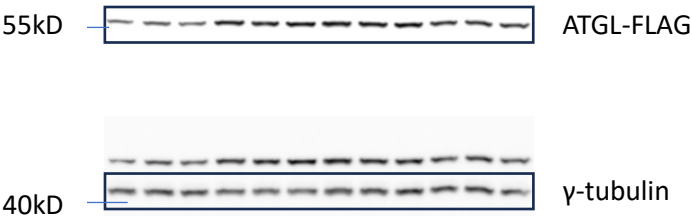

e and f

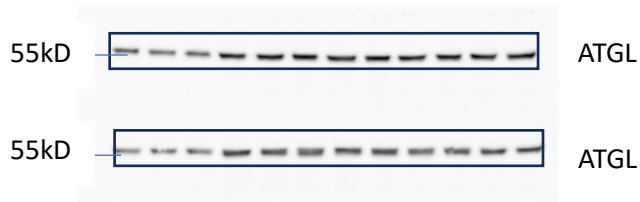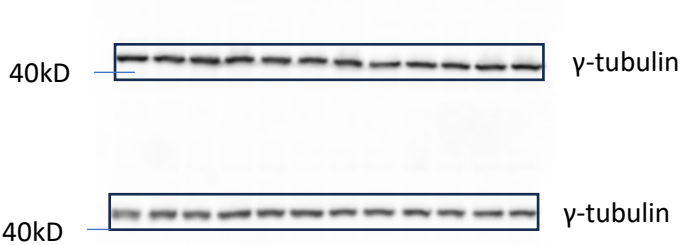

Supplement: Supplementary file 24 — Unprocessed western blot. [file 41556_2024_1386_MOESM24_ESM.pdf]

Extended Data Fig. 6

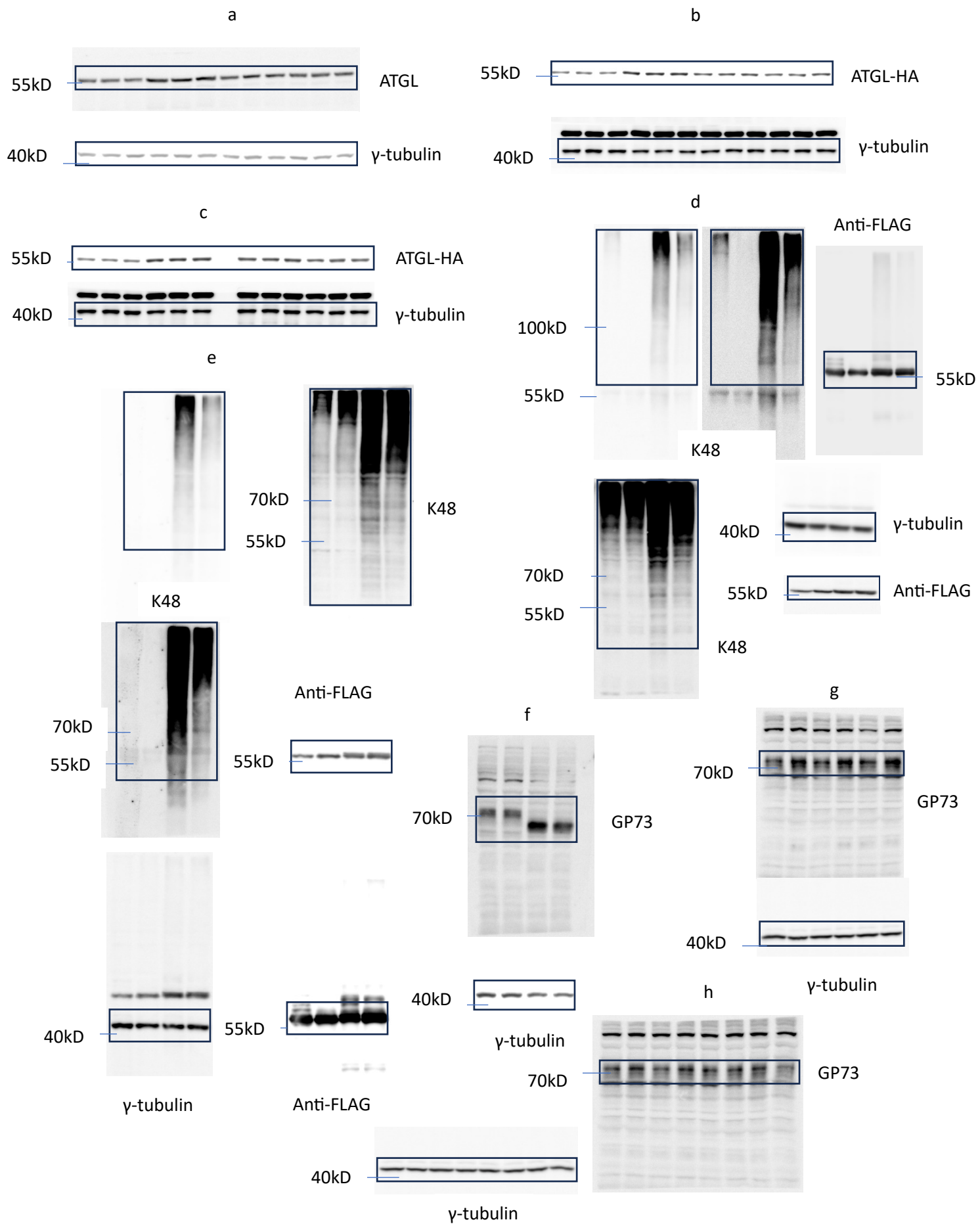

i

GP73

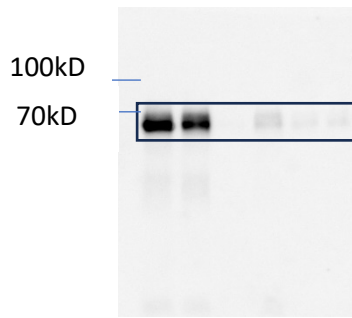

70kD

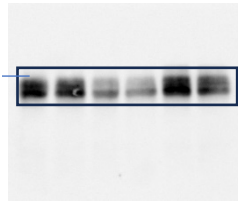

GP73

40kD

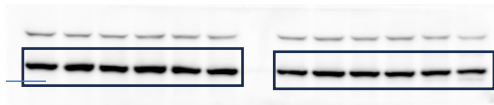

$\gamma$ -tubulin

k

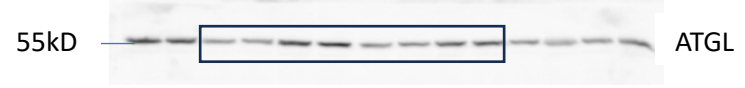

ATGL

180kD

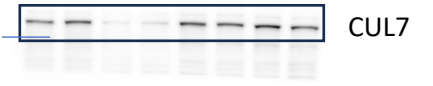

CUL7

55kD

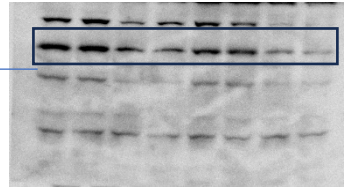

FBXW8

40kD

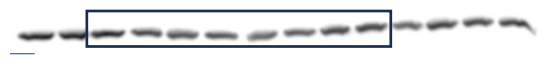

$\gamma$ -tubulin

Supplement: Supplementary file 26 — Unprocessed western blot. [file 41556_2024_1386_MOESM26_ESM.pdf]

Extended Data Fig. 7

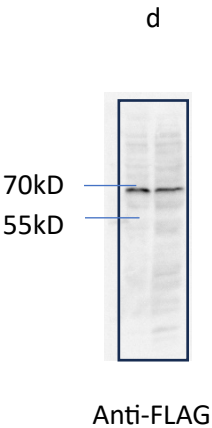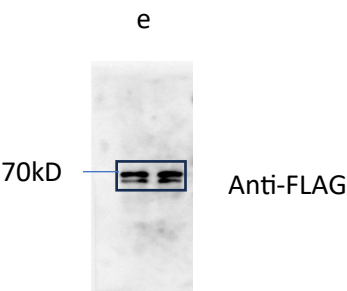

Supplement: Supplementary file 28 — Unprocessed western blot. [file 41556_2024_1386_MOESM28_ESM.pdf]

Extended Data Fig. 8

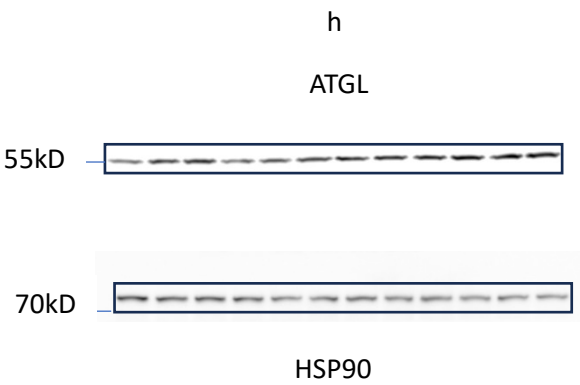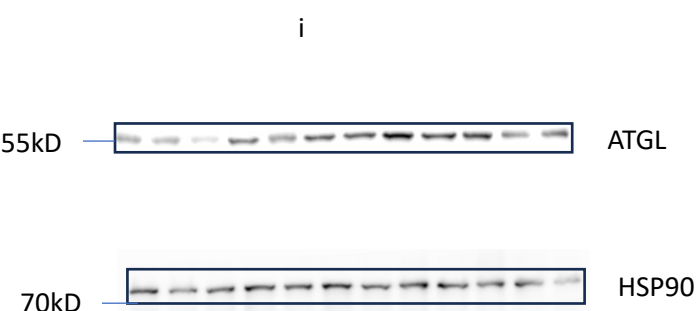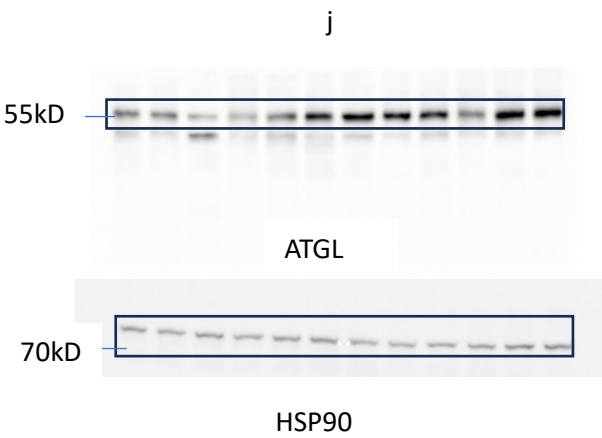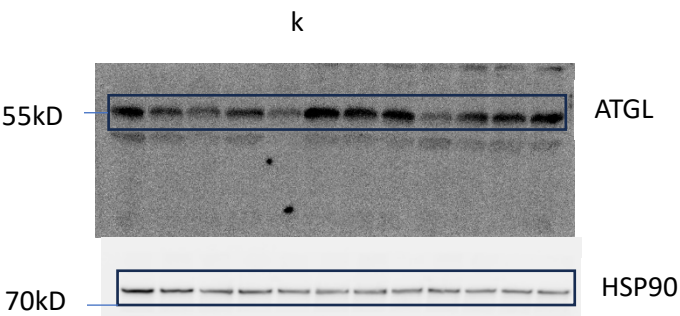

Supplement: Supplementary file 30 — Unprocessed western blot. [file 41556_2024_1386_MOESM30_ESM.pdf]

Extended Data Fig.9

c

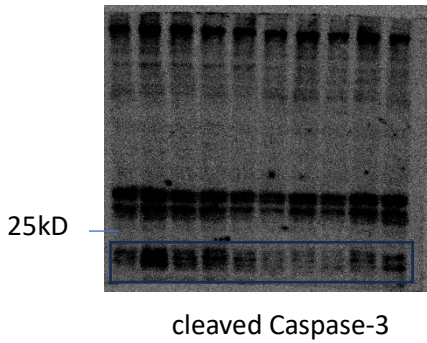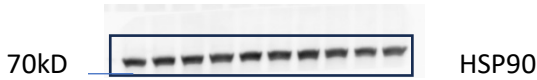

h

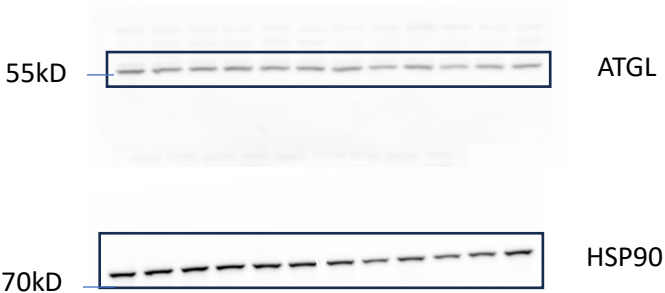

Supplement: Supplementary file 32 — Unprocessed western blot. [file 41556_2024_1386_MOESM32_ESM.pdf]
