# Supplementary material for: Glucose controls lipolysis through Golgi PtdIns4P-mediated regulation of ATGL
Source: Nat Cell Biol. 2024 Apr 1;26(4):552–66. doi: 10.1038/s41556-024-01386-y (PMC11021197; doi:10.1038/s41556-024-01386-y)

Extended Data Fig.1

a: Liver

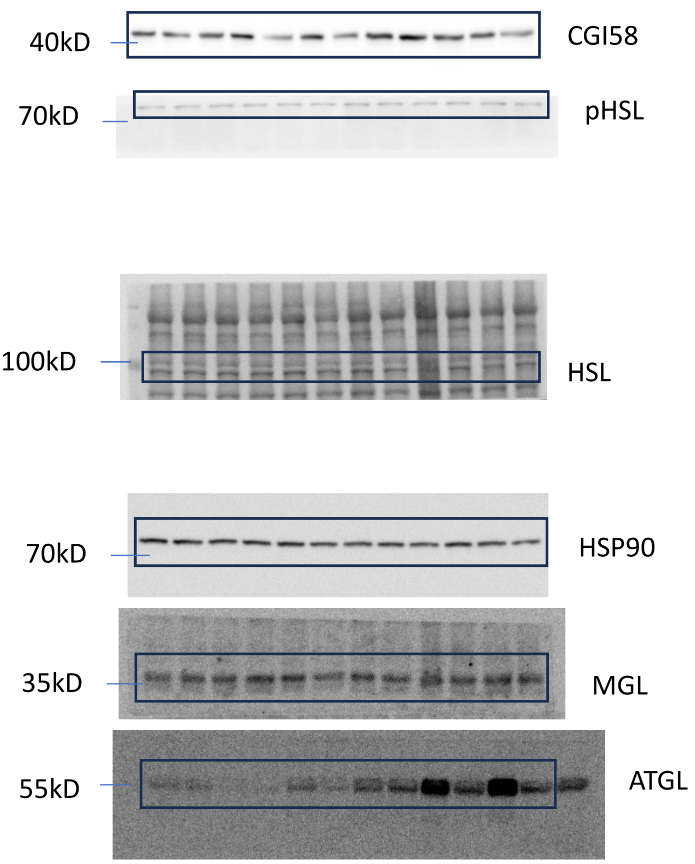

a: iBAT

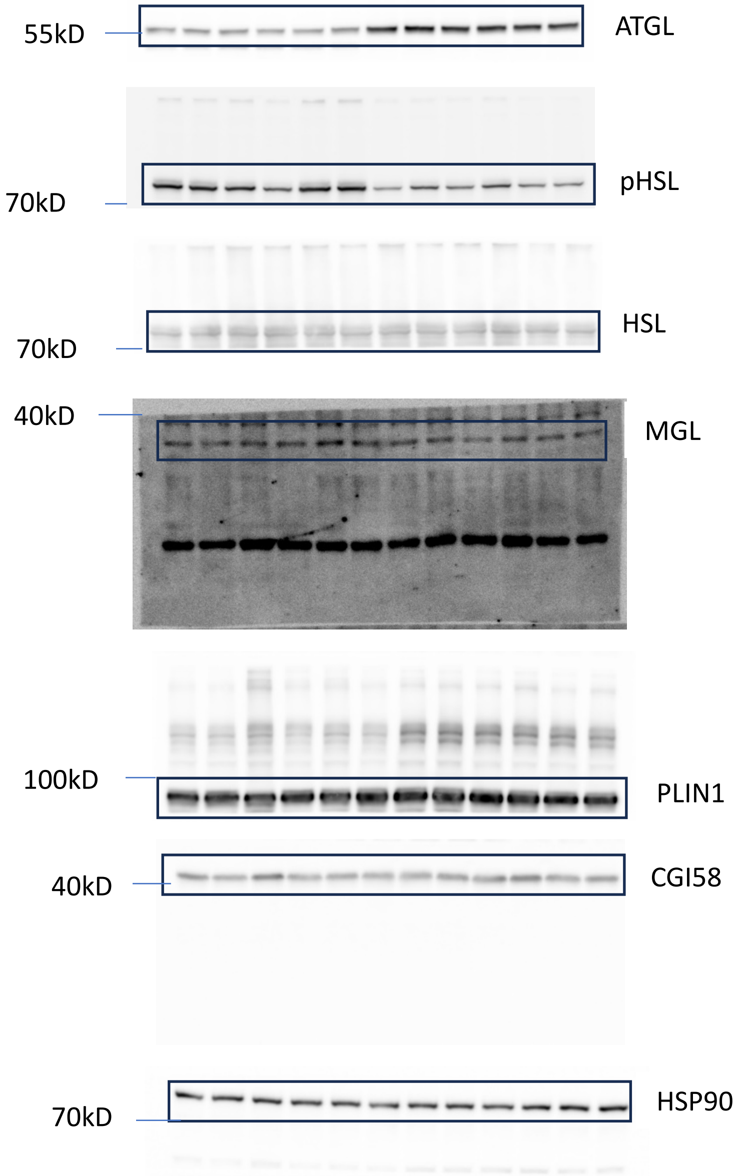

a: iWAT

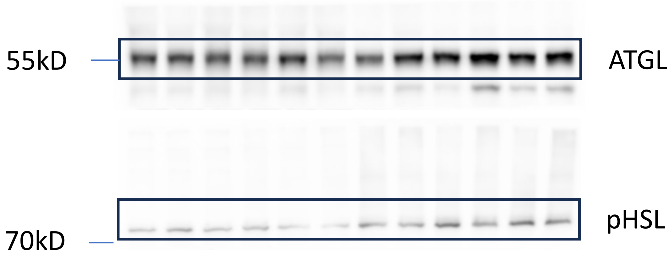

a: gWAT

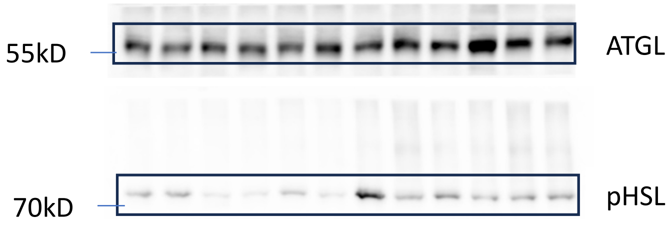

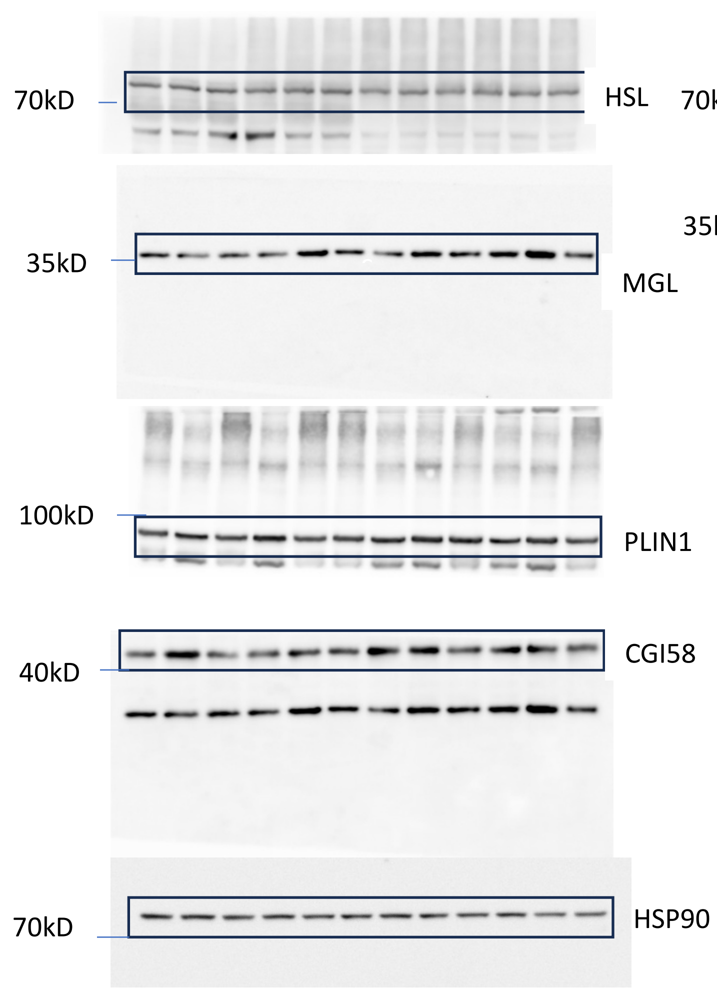

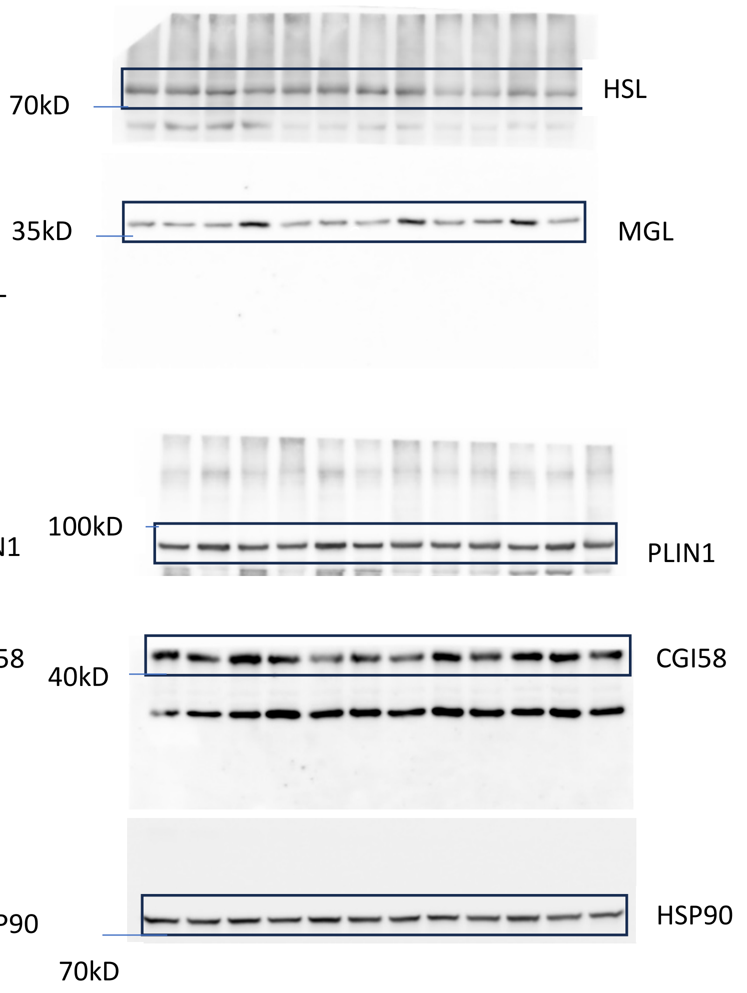

g: Liver 1h

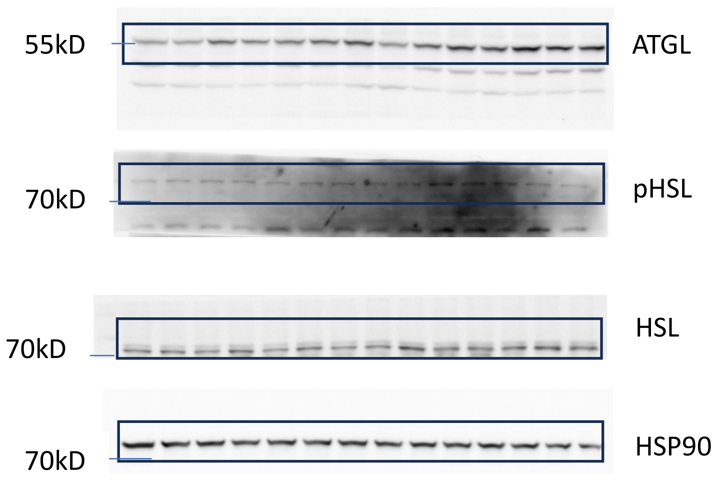

g: iBAT 1h

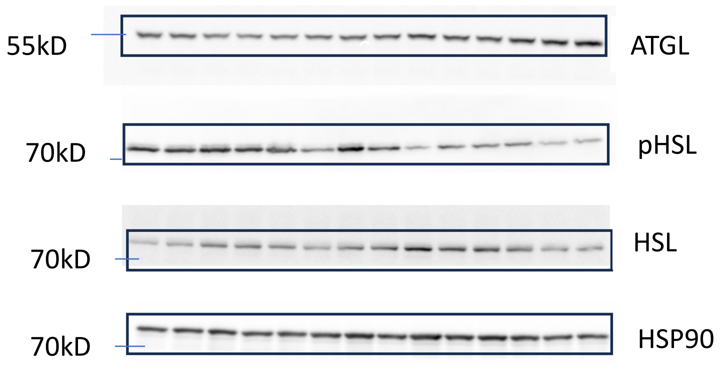

g: iWAT 1h

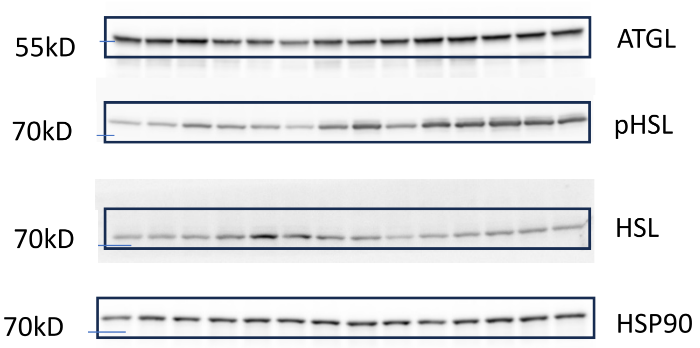

g: gWAT 1h

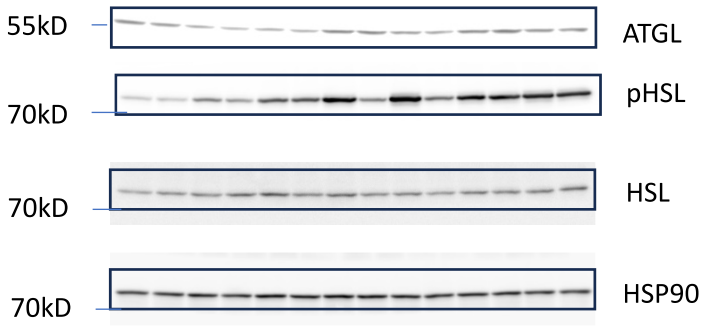

g: Liver 6h

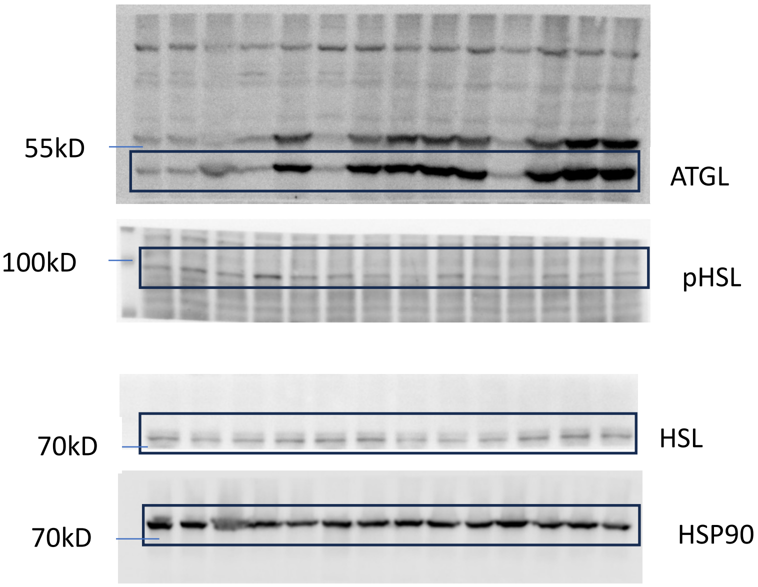

g: iBAT 6h

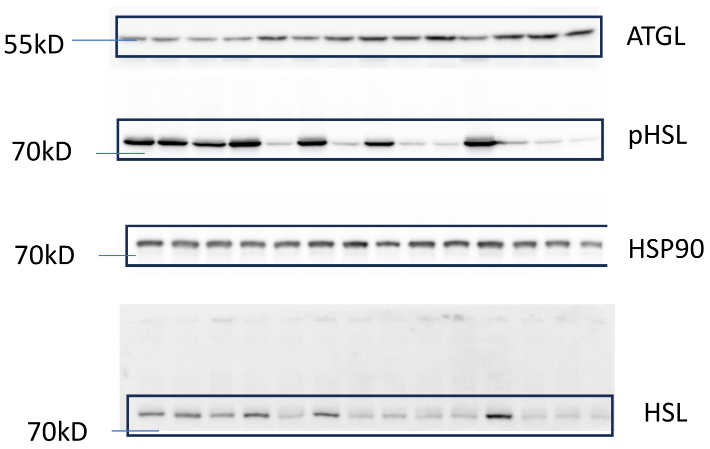

g: iWAT 6h

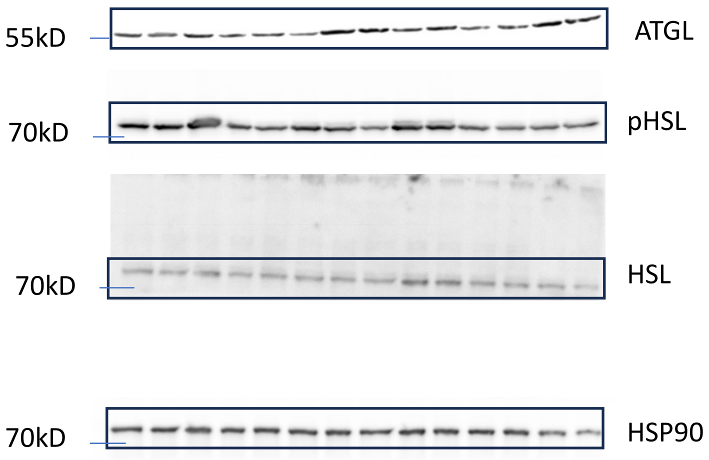

g: gWAT 6h

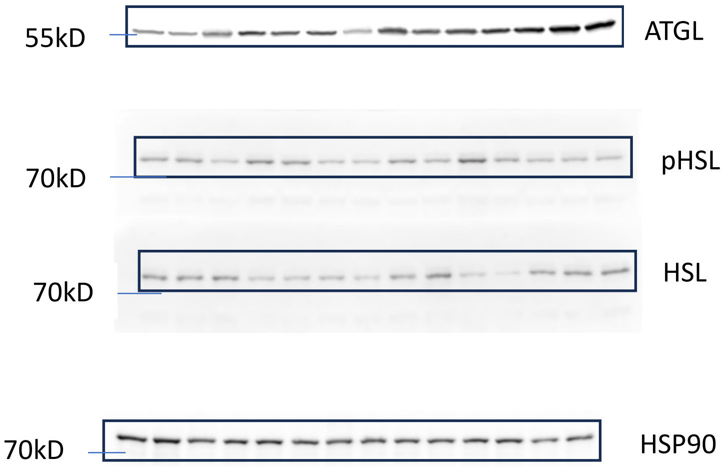

g: Liver 12h

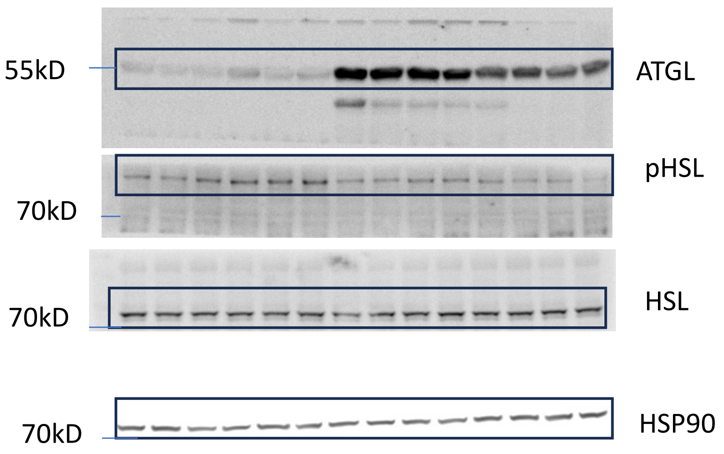

g: iBAT 12h

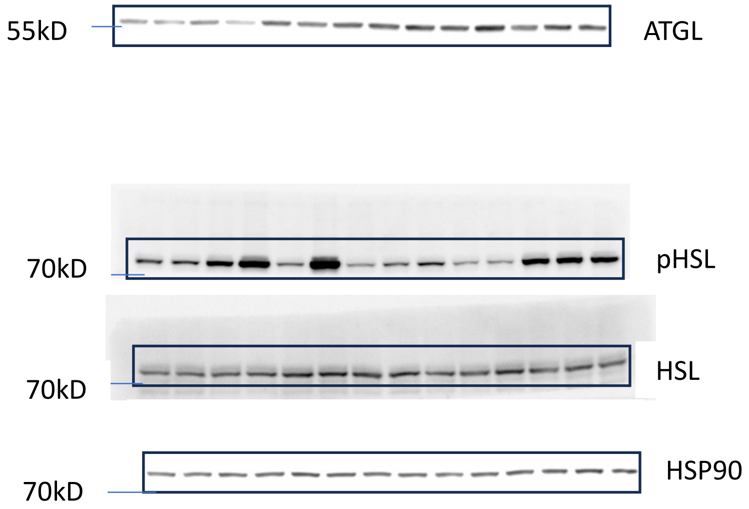

g: iWAT 12h

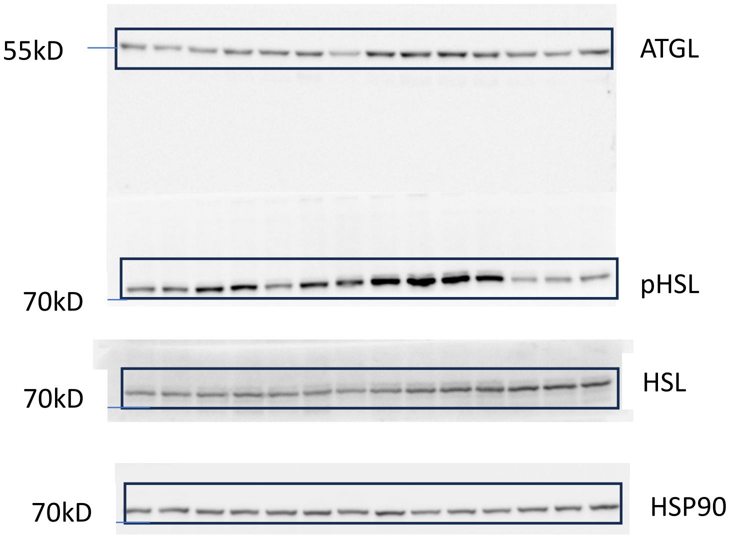

g: gWAT 12h

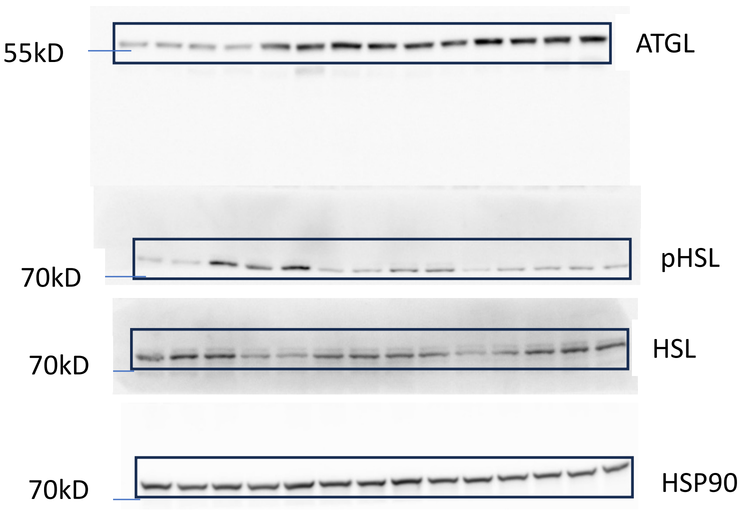

Supplement: Supplementary file 16 — Unprocessed western blot. [file 41556_2024_1386_MOESM16_ESM.pdf]
